# Countering vaccine hesitancy: a systematic review of interventions to strengthen healthcare professionals’ action

**DOI:** 10.1093/eurpub/ckad134

**Published:** 2023-08-15

**Authors:** Giuseppina Lo Moro, Maria Ferrara, Elisa Langiano, Davide Accortanzo, Toni Cappelletti, Aldo De Angelis, Maurizio Esposito, Alessandro Prinzivalli, Alessandra Sannella, Sara Sbaragli, Pia Vuolanto, Roberta Siliquini, Elisabetta De Vito

**Affiliations:** Department of Public Health Sciences and Paediatrics, University of Turin, Turin, Italy; Department of Human, Social and Health Sciences, University of Cassino and Southern Lazio, Cassino, Italy; Department of Human, Social and Health Sciences, University of Cassino and Southern Lazio, Cassino, Italy; Department of Public Health Sciences and Paediatrics, University of Turin, Turin, Italy; Department of Public Health Sciences and Paediatrics, University of Turin, Turin, Italy; Department of Public Health Sciences and Paediatrics, University of Turin, Turin, Italy; Department of Human, Social and Health Sciences, University of Cassino and Southern Lazio, Cassino, Italy; Department of Public Health Sciences and Paediatrics, University of Turin, Turin, Italy; Department of Human, Social and Health Sciences, University of Cassino and Southern Lazio, Cassino, Italy; Department of Human, Social and Health Sciences, University of Cassino and Southern Lazio, Cassino, Italy; Research Centre for Knowledge, Science, Technology and Innovation Studies of Tampere University, Tampere, Finland; Department of Public Health Sciences and Paediatrics, University of Turin, Turin, Italy; AOU City of Health and Science of Turin, Turin, Italy; Department of Human, Social and Health Sciences, University of Cassino and Southern Lazio, Cassino, Italy

## Abstract

**Background:**

Vaccine hesitancy is relevant for healthcare professionals (HCPs) who face challenges in building trusting relationships with patients. Accordingly, the VAX-TRUST project has been developed to improve experiences of HCPs and patients dealing with vaccinations. To support VAX-TRUST, this work aimed to identify latest interventions targeted at HCPs to address hesitancy and increase vaccine uptake.

**Methods:**

A systematic review was conducted according to PRISMA by searching PubMed, Scopus and Embase. The protocol was registered on PROSPERO. Articles were eligible if evaluated interventions directly targeted at HCPs/healthcare students. The search was run on 26 January 2022. Articles published in 2016 or after were included.

**Results:**

A total of 17 492 records were identified; 139 articles were selected. Most articles were set in USA (*n* = 110). Over half had a pre–post design without a control group (*n* = 78). A total of 41 articles focused on single-component interventions, 60 on multi-component interventions involving only HCPs and/or students and 38 on multi-component interventions involving also other professionals. Main components were in-person education (*n* = 76), synchronous (*n* = 10) and asynchronous (*n* = 23) online learning, educational materials (*n* = 26), performance assessment and feedback (*n* = 33), electronic record changes (*n* = 30), role play/simulation (*n* = 21) and online games/apps (*n* = 5). Educational sessions were mainly about scientific update or communication. Outcomes of interventions were grouped in: vaccination rates (*n* = 69), knowledge (*n* = 32), attitudes (*n* = 26), confidence in counselling (*n* = 30) and acceptability (*n* = 16).

**Conclusions:**

Apps, gaming, role play/simulations could represent innovative interventions. This review highlighted the need of delving into communication strategies and using more robust evaluations, longer follow-up and standardized measurements.

## Introduction

Vaccine hesitancy is a complex phenomenon that encompasses the concepts of indecision, uncertainty, delay and reluctance.^1^ It varies not only in terms of intensity, ranging from hesitant people who accept a vaccination albeit with important doubts to people who completely refuse it, but also by type of vaccine.^2^ Although vaccinations are considered one of the greatest successes of public health, a growing number of people are reluctant towards these measures^2^ and the World Health Organization (WHO) has identified vaccine hesitancy as 1 of the 10 greatest threats to global health.^3^

Vaccine hesitancy is a relevant phenomenon for healthcare professionals (HCPs) who face numerous challenges in building trusting relationships with their patients. The relationship between professionals and patients is fundamental: the way HCPs respond to hesitant patients is crucial, and HCPs who are informed and confident about vaccines are more likely to provide adequate messages.^4^^,^^5^ The crisis of trust towards HCPs is of increasing importance considering the expansion of alternative medicine and the use of ‘doctor Google’.^6^ A well-known hesitancy determinant is the lack of trust^2^^,^^7^ and the level of trust in information from HCPs has been reported to protect against misconceptions about vaccinations.^8^ During the pandemic, this crisis of confidence has increased: along with the distrust in politics and journalism, the pandemic has amplified the distrust in health services, which could lead to potential harm.^9^^,^^10^ As HCPs serve as points of reference, it is essential to support HCPs and students (i.e. future HCPs) by providing them with tools to contrast hesitancy and promote vaccine uptake.

Within this context, the ‘VAX-TRUST—Addressing Vaccine Hesitancy in Europe’ project has been developed to improve the experiences of HCPs and patients dealing with vaccinations. Among the objectives, VAX-TRUST aims to support HCPs in addressing hesitancy through the implementation of tailored interventions. To support the planning of these interventions and understand which methods could be used, researchers participating in VAX-TRUST decided to provide an overview of the multitude of possible interventions that have been recently implemented and evaluated, looking for possible innovative tools.

Therefore, this systematic review aimed primarily to identify the most recent interventions targeting HCPs and healthcare students to address vaccine hesitancy and increase vaccine uptake among their patients.

## Methods

### Search strategy and criteria

A systematic review was conducted according to the PRISMA guidelines^11^ by searching PubMed, Scopus and Embase. The protocol was registered on PROSPERO (CRD42022331459).

The search strategy was structured using the PICOS framework: HCPs or healthcare students (population); intervention to reduce vaccine hesitancy or increase vaccination rates in patients (intervention); any comparison, outcome and study design. A combination of free text and Medical Subject Medical Subject Headings (MeSH) was used. Three themes were linked with AND: vaccine AND hesitancy AND healthcare professional/student. Search terms are provided in Supplementary file S1. The search was run on 26 January 2022, including only articles published in 2016 or after. Duplicates were removed.

Articles were eligible if they evaluated interventions meeting two criteria: being directly targeted at HCPs or students who may interact with patients; aiming to reduce hesitancy or increase vaccine uptake among patients/parents. Articles were eligible even if these outcomes were not measured but represented the broad goal of the interventions, which were evaluated, for instance, measuring vaccine-related knowledge or communication skills. To be included, the intervention could be part of a broader intervention that included multiple actions, not necessarily targeted only at HCPs/students. Only primary studies were eligible.

Commentaries, research protocols and letters were excluded. The reference list of reviews that could potentially include primary articles meeting the inclusion criteria was searched to identify additional articles.

### Study selection and data extraction

Six authors independently screened records for title and abstract (M.F., E.L., A.S., G.L.M., A.P. and D.A.) through the web application Rayyan.^12^ Then, three authors (G.L.M., A.P. and D.A.) applied the inclusion/exclusion criteria to the selected full-texts, documenting the reasons for exclusion. In both phases, each article was screened at least by two researchers who were blinded to each other’s decisions.

Four authors (G.L.M., A.P., T.C. and A.D.A.) performed data extraction using an Excel spreadsheet. For each article, at least one researcher extracted the data and at least another one checked the extracted data. These data were extracted and grouped into categories when possible: author, year of publication, country, study design, participants’ characteristics, setting, year and length of interventions, involved vaccines, intervention characteristics (control group characteristics, if applicable), results of the evaluation of measured outcomes, presence of any declared conflicts of interest and funding and incentives for participation (if available). If articles reported multiple analyses, only those considering actions addressed to HCPs/students were extracted. Due to a great variety in interventions’ characteristics and evaluations, the authors grouped interventions and evaluations in macro-categories and reported the results through a narrative synthesis and summary of findings tables. Since multiple differences among outcome evaluations were found, the authors decided to classify results as follows: ‘significant’ if all outcomes measured within one domain reported significant improvement; ‘non-significant’ if all outcomes measured within one domain reported non-significant results; ‘mixed’ or ‘conflicting’ if the outcomes measured within one domain reported both significant and non-significant results. In some cases, the authors reported that the articles did not evaluate the statistical significance of their findings only providing descriptive or qualitative analysis; in these cases, if improvements in the domain were described, the results were reported as ‘promising’, for instance.

Four authors (G.L.M., A.P., T.C. and A.D.A.) assessed the risk of bias of studies that included at least a pre–post evaluation. Each article was assessed by at least two researchers who were blinded to each other’s decisions. The evaluation was conducted with reference to the primary outcomes of articles. Non-randomized studies were assessed using the Joana Briggs Institute (JBI) Critical Appraisal Checklist for Quasi-Experimental Studies.^13^ Randomized controlled trials (RCTs) were evaluated with the revised tool to assess risk of bias in randomized trials (RoB-2) and cluster RCTs with the RoB-2 for cluster RCTs.^14^

In every phase, disagreements were resolved through consensus-based discussions.

## Results

**Figure 1:**
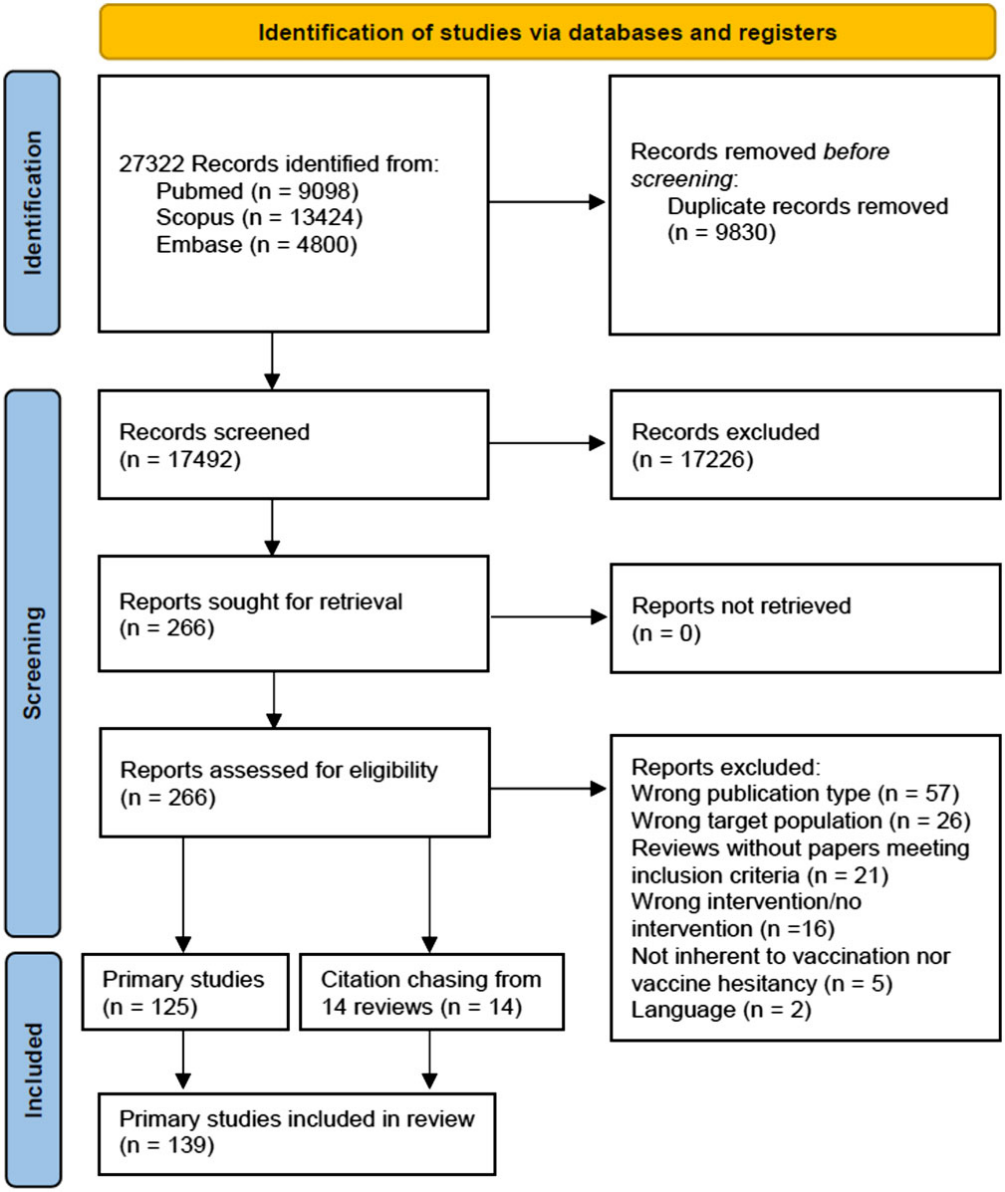
Flow diagram

A total of 17 492 unique records were identified and 139 articles were selected (figure 1). All articles were in English. All the details are reported in Supplementary file S2.

The years when more articles were published were 2020 (*n* = 32) and 2021 (*n* = 32). The studies were conducted between 2010 (*n* = 1) and 2021 (*n* = 3); the years during which more studies began were 2015 (*n* = 22), 2016 (*n* = 20) and 2018 (*n* = 20). Most articles were set in the USA (*n* = 110), followed by Europe and the UK (*n* = 11). Over half had a pre–post design without a control group (*n* = 78), followed by cluster RCTs (*n* = 20), non-randomized controlled trials (*n* = 16), interventional studies with only post-intervention evaluation (*n* = 16), qualitative studies (*n* = 7) and RCTs (*n* = 2). In the case of controlled trials, no intervention/usual care was the most frequent control group (*n* = 18).

Most articles targeted HCPs (*n* = 116), while 20 articles focused on students. Three articles considered both populations. When specified (often, ‘all staff’ of a practice was involved, without details), the most frequently involved professionals were medical doctors (*n* = 37), nurses (*n* = 23) and pharmacists (*n* = 10). Among students, medical (*n* = 14), nursing (*n* = 8) and pharmacy (*n* = 4) students were primarily involved. As incentives to participate in the study, some authors indicated credits (e.g. Continuing Medical Education credits) (*n* = 21), rewards (e.g. small gifts or economic incentives) (*n* = 9) or the inclusion of the intervention in curricular lessons or hospital/centre's usual lectures (*n* = 16). The most frequent primary aims of the interventions were increasing vaccinations rates among patients (*n* = 67), improving confidence/self-efficacy in communication with patients/parents and enhancing the ability to promote vaccinations (*n* = 45), increasing vaccine-related knowledge (*n* = 34) and changing attitudes/perceptions towards vaccines (*n* = 26). Only 33 studies were about vaccines/hesitancy in general, while the others focused on specific vaccines. Articles dedicated to one vaccine were more frequently about HPV (*n* = 57) and influenza vaccine (*n* = 20).

As explained in Supplementary file S2 (‘Extended results’ section), several works referred to the same interventions, thus unique interventions were 127. A total of 41 articles focused on single-component interventions (37 interventions), 60 on multi-component interventions involving only HCPs and/or students (55 interventions) and 38 on multi-component interventions involving other targets too (35 interventions).

The actions composing the interventions were grouped into nine categories: in-person educational sessions (e.g. traditional methods as lectures, PowerPoint slides and Question & Answer sessions) (*n* = 76); online educational sessions, synchronous (e.g. live webinars) (*n* = 10) or asynchronous learning (i.e. forms of education that do not occur in the same place or at the same time, such as video lessons, tutorials, readings, audios and images to engage the participant in interactive learning) (*n* = 23); provision of educational material and reminders containing educational materials (*n* = 26); changes in the electronic health records (EHRs) directly addressed to HCPs (e.g. alerts, prompts and standing orders) (*n* = 30); assessment of vaccination performance, personal or of the entire service and feedback (e.g. immunization report cards) (*n* = 33); role play (i.e. simulations where learners represent different character roles) and scenarios with simulated patients (i.e. involving people dedicated to play the role of the patients, e.g. professional actors, trained facilitators) (*n* = 21); digital tools, e.g. online games and apps (*n* = 5); art-based tools, e.g. video vignettes (*n* = 3); other. The main topics of in-person and online sessions were scientific updates (i.e. information on vaccine-preventable diseases and vaccines) or communication strategies.

The outcomes measured to evaluate the interventions were grouped into seven domains: vaccination rates and missed opportunities (i.e. contact that does not result in the individual getting the vaccines for which he/she is eligible) (*n* = 69), vaccination-related knowledge (*n* = 32), beliefs/attitudes/perceptions towards vaccinations and hesitancy (*n* = 26), self-efficacy to recommend the vaccine and confidence/comfort in vaccination counselling (*n* = 30), perceived usefulness of the intervention, acceptability, satisfaction and reactions of participants (*n* = 16), outcomes measured through qualitative methods (mostly about acceptability, satisfaction, reactions of participants and perceived confidence in addressing vaccine-hesitant individuals) (*n* = 22) and other. Overall, acceptability and satisfaction were always good (*n* = 34).

Most articles disclosed not having conflict of interests (*n* = 96) and received funding (*n* = 86). Considering the risk of bias for non-randomized studies, the items that reported more frequently issues were: absence of control groups (*n* = 78), absence of multiple measurements of the primary outcome both pre- and post-intervention (*n* = 45) and not adequately described completion of follow-up (*n* = 29). For RCT/cluster RCT, the risk of bias was generally low. However, the two RCTs presented some concerns in the randomization process, one cluster RCT presented a high risk of bias in missing outcome data and another cluster RCT evaluation reported some concern in outcome measurement.

A summary of the main findings of each intervention is provided in the section ‘Details on the main results of the intervention evaluation’ of Supplementary file S2.

### Single-component interventions

Details are shown in table 1 and Supplementary file S2 (‘Extended results’). These interventions lasted from 45 min (in-person session) to 3 years (EHR changes).

**Table 1:** Single-component interventions: main characteristics of the interventions

Author and year	In-person educational session		Asynchronous learning		Online game, apps	Interventions on EHR: alerts, prompts, standing orders	Role play or scenario simulation	Training-of-trainers	Educational material	Main results of the evaluation
	**SU**	**CS**	**SU**	**CS**						
Interventions targeted at healthcare professionals										
Srirangan K. 2021^a^	X	X	.	.	.	.	.	.	.	+
Serino L. 2020^b^	X	.	.	.	.	.	.	.	.	+
Abdulla E. 2020^c^	X	.	.	.	.	.	.	.	.	+ −
Torabizadeh C. 2020^c^	X	.	.	.	.	.	.	.	.	+
Gagneur A. 2019^c^	.	X	.	.	.	.	.	.	.	+
Lin J.L. 2018^d^	X	X	.	.	.	.	.	.	.	+
Brewer N.T. 2017^e^	X	X	.	.	.	.	.	.	.	+ −
Dybsand L.L. 2019^a^	X	X	.	.	.	.	.	.	.	+
O’Donnell M. 2018^c^	X	.	.	.	.	.	.	.	.	+
Blake H. 2022^a^	.	.	X	X	.	.	.	.	.	+
Szilagyi P.G. 2021^e^	.	.	X	X	.	.	.	.	.	+ −
McFadden S.M. 2021^c^	.	.	X	X	.	.	.	.	.	+
Cates J.R. 2020^b^	.	.	X	X	.	.	.	.	.	+
Chamberlain A.T. 2019^b^	.	.	.	X	.	.	.	.	.	+
Real F.J. 2017^d^	.	.	.	.	X	.	.	.	.	+
Heaton P.C. 2022^e^	.	.	.	.	.	X	.	.	.	+
Frederick K.D. 2020^a^	.	.	.	.	.	X	.	.	.	+
Cieslowski B. 2020^a^	.	.	.	.	.	X	.	.	.	+ −
Persell S.D. 2020^c^	.	.	.	.	.	X	.	.	.	+
Wilkinson T.A. 2019^e^	.	.	.	.	.	X	.	.	.	−
Kim R.H. 2018^d^; Patel M.S. 2017^d^	.	.	.	.	.	X	.	.	.	+
Krishnaswamy S. 2018^d^	.	.	.	.	.	X	.	.	.	+
Zimet G. 2017^e^	.	.	.	.	.	X	.	.	.	+ −
Amare A.T. 2021^a^	.	.	.	.	.	.	.	X	.	+
Tchoualeu D.D. 2021^c^; Traicoff D. 2021^b^	.	.	.	.	.	.	.	X	.	+
Arogundade L. 2019^a^	.	.	.	.	.	.	.	X	.	+
Jones K.M. 2016^c^	.	.	.	.	.	.	.	.	X	+
Interventions targeted at students										
Visalli G. 2021^c^	X	.	.	.	.	.	.	.	.	+
Bradley C.L. 2021^c^	X	X	.	.	.	.	.	.	.	+
Berenson A.B. 2021^c^; Berenson A.B. 2020^c^	X	.	.	.	.	.	.	.	.	+
Bechini A. 2019^c^	X	X	.	.	.	.	.	.	.	+
Cotter J.C. 2019 ^c^	X	X	.	.	.	.	.	.	.	+
Marotta C. 2017^c^	X	X	.	.	.	.	.	.	.	+ −
Wiley R. 2017^c^	X	X	.	.	.	.	.	.	.	+
Mitchell G. 2021^c^	.	.	.	.	X	.	.	.	.	+
Chang C.Y. 2021^a^	.	.	.	.	X	.	.	.	.	+
Nold L. 2020^a^	.	.	.	.	.	.	X	.	.	+

Notes: References are provided in Supplementary file S2. Torabizadeh C. 2020: targeted at both healthcare professionals and students.

a Qualitative or mixed method design.

b Only post-intervention evaluation.

c Uncontrolled pre–post study.

d Non-randomized controlled trial.

e (Cluster) randomized controlled trial.

CS, communication strategies; EHR, electronic health record; SU, scientific updating; +, encouraging results in favour of the intervention; + −, conflicting results; −, non-significant results.

In-person sessions’ topics were scientific update (*n* = 6), communication strategies (*n* = 1) or both (*n* = 9). Concerning communication, some interventions focused on motivational interviewing (*n* = 1), announcement training (*n* = 1) or both (*n* = 1). Most articles analyzed the impact on knowledge, attitudes and confidence/comfort in managing patients (*n* = 13), showing contrasting results. Vaccination rates were considered for two interventions: results were conflicting.

Asynchronous learning was on both scientific updating and communication (*n* = 4). The most frequent outcome was confidence (*n* = 4), showing promising results. Similar results were reported for knowledge and attitudes (*n* = 3).

Among digital tools, one virtual reality game with scenario simulations showed a significant reduction in vaccination refusal among participants’ patients. One serious game and one chatbot-based learning approach significantly improved knowledge.

Interventions addressed to HCPs through EHR modifications were frequent (*n* = 8). The effect on vaccination rates was mixed.

Lastly, other interventions were training of trainers (*n* = 2), one revision of a toolkit with immunization information and a scenario simulation for students.

### Multiple-component interventions exclusively addressed to HCPs/students

Details are shown in table 2 and Supplementary file S2 (‘Extended results’). These interventions lasted from 20 min (intervention including role play/simulation) to 15 months (intervention involving EHR changes).

**Table 2:** Multiple-component interventions exclusively addressed to HCPs or students: main characteristics of the interventions

Author and year	Interventions on EHR: alerts, prompts, standing orders	Assessment and feedback	Role play or scenario simulation	In-person educational session		Synchronous online learning		Asynchronous learning		Educational materials and reminders	Online game, apps	Arts based tools	Training-of-trainers	Other	Main results of the evaluation
				**SU**	**CS**	**SU**	**CS**	**SU**	**CS**						
Interventions targeted at healthcare professionals															
Gilkey M.B. 2019^a^	X	X	.	X	X	.	.	.	.	X	.	X	.	.	− +
Sandokji I. 2021^b^	X	X	.	X	.	.	.	.	.	X	.	.	.	.	+
Rand C.M. 2018a^b^; Rand C.M. 2018b^b^	X	X	.	.	.	.	X	.	.	.	.	.	.	Telephone calls; practice teams	− +
Rao S. 2020^b^	X	X	.	.	.	.	.	X	.	.	.	.	.	.	+
Werk L.N. 2019^a^	X	X	.	.	.	.	.	X	.	.	.	.	.	.	− +
Bratic J.S. 2019^b^	X	.	.	X	.	.	.	.	.	.	.	.	.	.	− +
Buenger L.E. 2020^b^	X	.	.	X	.	.	.	.	.	.	.	.	.	.	+
Stetson R.C. 2019^b^	X	.	.	.	.	.	.	.	.	X	.	.	.	.	+
Steiner C.R. 2021^b^	X	.	.	.	.	.	.	.	.	X	.	.	X	.	−
Giduthuri J.G. 2019^a^	.	X	.	X	.	.	.	.	.	.	.	.	.	.	++
Bonville C.A. 2019^a^	.	X	.	X	.	.	.	.	.	.	.	.	.	Quality improvement training	+
Irving S.A. 2018^c^	.	X	.	X	X	.	.	.	.	.	.	.	.	.	−
Whitaker J.A. 2018^a^	.	X	.	X	X	.	.	.	.	.	.	.	.	.	− +
Bradley-Ewing A. 2021^a^	.	X	.	.	X	.	.	.	.	.	.	.	.	Behavioural nudges	−
Hastings T.J. 2019^a^	.	X	.	.	.	X	.	X	.	.	.	.	.	.	− +
Oliver K. 2020^b^	.	X	.	.	.	X	X	.	.	.	.	.	.	Quality improvement training	− +
Fiks A.G. 2016^c^	.	X	.	.	.	X	.	.	.	.	.	.	.	.	− +
Wallace-Brodeur R. 2020^b^	.	X	.	.	.	X	X	.	.	.	.	.	.	Quality improvement training	+
Kawczak S. 2020^c^	.	X	.	.	.	.	.	X	.	.	.	.	.	.	− +
Loiacono M.M. 2021^a^	.	X	.	.	.	.	.	.	.	X	.	.	.	.	− +
Spina C.I. 2020^b^	.	X	.	.	.	.	.	.	.	.	.	.	.	Incentives	+
Malo T.L. 2018^a^	.	.	X	X	X	.	.	.	.	.	.	.	.	.	+
Dawson R. 2018^b^	.	.	X	X	X	.	.	.	.	.	.	.	.	.	− +
Rosen B.L. 2021^d^	.	.	X	X	X	.	.	.	.	.	.	.	.	.	+
Morhardt T. 2016^d^	.	.	X	X	X	.	.	.	.	.	.	.	.	.	+
Evans L. 2019^b^	.	.	X	X	X	.	.	.	.	.	.	.	.	.	+
Fiorito T.M. 2021^b^	.	.	X	X	X	.	.	.	.	.	.	.	.	.	+
Austin J.D. 2020^d^	.	.	X	X	X	.	.	X	X	X	.	.	.	.	+
Chen H. 2020^b^	.	.	X	X	.	.	.	.	.	.	.	.	.	.	+
Glanternik J.R. 2020^b^	.	.	X	.	X	.	.	.	.	.	.	.	.	.	− +
Brewer N.T. 2021^c^	.	.	X	.	X	.	.	.	.	.	.	.	X	.	− +
Maurici M. 2019^b^	.	.	X	.	X	.	.	.	.	.	.	.	.	.	+
Kumar M.M. 2019^b^	.	.	X	.	.	.	.	X	X	.	.	.	.	.	− +
Gatwood J. 2021^a^	.	.	X	.	.	.	.	X	X	.	.	.	.	.	−
Pahud B. 2020^a^	.	.	X	.	.	.	.	X	X	.	.	.	.	.	− +
Salous M.H. 2020^b^	.	.	.	X	X	.	.	.	.	X	.	.	.	.	− +
Shukla A. 2018^e^; Pampena E. 2019^b^	.	.	.	X	X	.	.	.	.	X	.	.	.	.	+
Wermers R. 2021^b^	.	.	.	X	X	.	.	.	.	X	.	.	.	.	−+
Brodie N. 2018^b^	.	.	.	X	X	.	.	.	.	X	.	.	.	.	+
Barton S.M. 2022^c^	.	.	.	.	X	X	.	.	.	.	.	.	.	.	− +
Abdalla A. 2021^b^	.	.	.	X	.	.	.	.	.	X	.	.	.	.	− +
Percy J.N 2019^d^	.	.	.	.	.	X	.	.	.	.	.	.	X	.	−
Skoy E. 2020^b^	.	.	.	.	.	.	.	X	.	X	.	.	.	.	+
Williams S.E. 2021^a^	.	.	.	.	.	.	.	X	X	X	.	.	.	Quality improvement training	− +
Ciemins E.L. 2020^d^	.	.	.	X	.	X	.	.	.	.	.	.	.	Learning collaborative model	+
Bishop J.M. 2021^b^; Real F.J. 2021^d^	.	.	.	.	.	.	.	X	.	.	X	.	.	.	+
Interventions targeted at students															
Schnaith A.M. 2018^b^	.	.	X	X	.	.	.	.	.	.	.	X	.	.	+
Onello E. 2020^b^	.	.	X	X	X	.	.	.	.	.	.	.	.	.	+
Vyas D. 2018^b^	.	.	X	X	X	.	.	X	.	.	.	.	.	.	+
Chase A.J. 2020^d^	.	.	X	.	X	.	.	.	.	.	.	.	.	.	+
Coleman A. 2017 ^e^	.	.	X	.	.	.	.	X	X	.	.	.	.	Podcast	+
Chidume T. 2020^e^	.	.	X	.	.	.	.	X	X	.	.	.	.	.	+
Chen G. 2021^b^	.	.	.	X	.	.	.	.	.	.	.	.	.	Learning and practicing intramuscular injections	+
Lepiller Q. 2020^b^	.	.	.	.	.	.	.	X	.	.	.	.	.	Preparing and managing a primary prevention intervention	+
Koski K. 2018^d^	.	.	.	.	.	.	.	.	.	.	.	X	.	Writing exercise	+

Notes: References are provided in Supplementary file S2.

a (Cluster) randomized controlled trial.

b Uncontrolled pre–post study.

c Non-randomized controlled trial.

d Qualitative or mixed method design.

e Only post-intervention evaluation.

CS, communication strategies; her, electronic health record; SU, scientific updating; +, encouraging results in favour of the intervention; + −, conflicting results; −, non-significant results.

Most interventions (*n* = 41) included at least one action among EHR changes, performance assessment and feedback and role play/simulation combined with educational session (in-person or online) or material.

Among EHR interventions (*n* = 9), five were combined with performance assessment and feedback. These actions were accompanied by in-person sessions and educational material (*n* = 2), synchronous (*n* = 2) or asynchronous learning (*n* = 2). Overall, these interventions were evaluated through vaccination rate changes, showing mixed results.

The remaining four EHR interventions were combined with in-person sessions (*n* = 2) or educational materials (*n* = 2). Vaccination rate improvements were not consistent.

Beyond EHR interventions, 12 interventions included assessment and feedback, matched with in-person sessions (*n* = 5), online learning (*n* = 5), educational material (*n* = 1) or incentives (*n* = 1). One intervention focused on motivational interviewing. Four evaluations reported significant improvements in vaccination rates, while most results were mixed or non-significant (*n* = 6).

Considering role play/simulation (*n* = 20), 15 interventions were combined with in-person sessions (generally including communication as main topic). Some interventions focused on announcement approach (*n* = 2), conversational approach (*n* = 1) or both (*n* = 1). Few works analyzed vaccination rates, showing mixed results (*n* = 3). Evaluations reported significant improvement in knowledge, attitudes/perceptions or confidence (*n* = 11). The other role play/simulation interventions were combined with asynchronous learning (*n* = 5). In this case, results in knowledge, attitudes/perceptions and confidence were mainly conflicting or non-significant.

Overall, interventions not including EHR changes, assessment and feedback or role play/simulation were a combination of in-person sessions, synchronous or asynchronous learning and sharing of materials (*n* = 11). Results on vaccination rates, knowledge, attitudes and confidence were not consistent across these studies.

Considering other combinations, asynchronous learning was paired with smartphone app based on evidence-based recommendations or with an exercise in which students managed a primary prevention intervention, showing significant improvement in knowledge, attitudes or self-efficacy. In-person session was utilized alongside practicing muscular injections, reporting significant improvement in knowledge, attitudes and confidence. Lastly, one intervention integrated videos showcasing interactions with patients and a writing exercise in which students responded as physicians.

### Multiple-component interventions not exclusively addressed to HCPs

Details are presented in table 3 and Supplementary file S2 (‘Extended results’). The main components not addressed to HCPs targeted patients or parents (e.g. education, posters, brochures, reminders, recalls, implementation of more convenient services and communication campaigns) or included changes of the workflow or organizational interventions to make vaccines available. Most interventions had long duration, ranging from 3 months to 5 years.

**Table 3:** Multiple-component interventions not exclusively addressed to HCPs: main characteristics of the interventions

Author and date	Interventions on EHR: alerts, prompts, standing orders	Assessment and feedback	In-person educational session		Synchronous online learning		Asynchronous learning		Educational material and reminders	Online game, apps	Other	Target of other components	Main results of the evaluation
			**SU**	**CS**	**SU**	**CS**	**SU**	**CS**					
Nissen M. 2019^a^	X	X	X	.	.	.	.	.	.	.	.	Patients/parents	+
Marchand-Ciriello L. 2020^a^	X	X	X	X	.	.	.	.	.	.	.	Patients/parents	−
Olshefski R.S. 2018^a^	X	X	X	X	.	.	.	.	X	.	.	Patients/parents	+
Vinci D.M. 2021^a^	X	X	X	.	.	.	.	.	.	.	.	Patients/parents	− +
Mazzoni S.E. 2016^a^	X	X	X	.	.	.	.	.	.	.	Vaccination champions	Patients/parents	+
Lin C. 2016^b^; Nowalk M.P. 2017^a^; Zimmerman R.K. 2017^b^	X	X	.	X	.	.	.	.	.	.	.	Patients/parents	− +
McGaffey A. 2019^a^	X	X	X	X	.	.	.	.	X	.	.	Patients/parents Change of the workflow	− +
Farmar A.M. 2016^c^	X	X	X	X	.	.	.	.	.	.	.	Change of the workflow Organizational intervention on the availability of vaccines	+
Deshmukh U. 2018^a^	X	.	X	.	.	.	.	.	.	.	Vaccination champions	Patients/parents	+
Orefice R. 2019^a^	X	.	X	.	.	.	.	.	.	.	Vaccination champions	Patients/parents	+
Dehlinger C. 2021^a^	X	.	X	X	.	.	.	.	X	.	.	Patients/parents	+
Jina a. 2019^a^	X	.	X	.	.	.	.	.	X	.	.	Parents/patients Organizational intervention on the availability of vaccines	+
O’Leary S.T. 2019^b^	X	.	X	.	.	.	.	.	.	.	.	Organizational intervention on the availability of vaccines	−
Perkins R.B. 2020a^b^; Perkins R.B. 2020 b^d^; Drainoni M.L. 2021^d^	.	X	X	X	.	.	.	.	.	.	.	Patients/parents	+
McLean H.Q. 2017^e^	.	X	X	X	.	.	.	.	.	.	.	Patients/parents	− +
Boey L. 2021^a^	.	X	X	.	.	.	.	.	X	.	.	Patients/parents	− +
Choi N. 2017^a^	.	X	X	.	X	.	.	.	.	.	Incentives	Patients/parents	+
Jacobs-Wingo J.L. 2017^a^	.	X	X	.	.	.	.	.	.	.	.	Patients/parents	+
Cates J.R. 2018^e^	.	X	X	.	.	.	.	.	.	.	Sharing practices between participants	Patients/parents	+
Malone K. 2016^a^	.	X	.	.	.	.	.	.	X	.	.	Patients/parents	+
Gingold J.A. 2016^d^	.	X	.	.	.	.	X	.	.	.	Quality improvement training	Patients/parents	+
Fisher-Borne M. 2018^b^	.	.	X	X	.	.	.	.	.	.	.	Patients/parents	− +
Sanderson M. 2017^e^	.	.	X	.	.	.	.	.	.	.	.	Patients/parents	− −
Spelman J.F. 2022^c^	.	.	X	.	.	.	.	.	.	.	Vaccination champions	Patients/parents	+
Giles M.L. 2021^a^	.	.	X	.	.	.	.	.	X	.	.	Patients/parents	+
Suryadevara M. 2019^a^	.	.	X	X	.	.	.	.	.	.	Vaccination champions	Patients/parents	+
Leila R.A. 2021^a^	.	.	.	X	.	.	.	.	.	.	.	Patients/parents	+
Dempsey A.F. 2018^b^; Reno J.E. 2018^a^; Reno J.E. 2018^a^	.	.	.	X	.	.	.	X	.	.	.	Patients/parents	+
Chin J. 2021^a^	.	.	X	.	.	.	.	.	.	.	.	Patients/parents Change of the workflow	+
Casalino E. 2018^a^	.	.	X	X	.	.	.	.	X	.	.	Organizational intervention on the availability of vaccines	+
Kepka D. 2021^a^	.	.	.	.	X	X	.	.	X	.	.	Patients/parents	+
Costello J. 2019^a^	.	.	.	.	.	.	.	.	X	.	.	Organizational intervention on the availability of vaccines	+
Garbutt J.M 2018^d^	.	.	.	.	.	.	.	.	X	.	.	Patients/parents	+
Kaufman J. 2020^b^	.	.	.	.	.	.	.	X	X	.	Vaccination champions	Patients/parents	+
Zaidi S. 2020^d^	.	.	.	.	.	.	.	.	.	X	.	Patients/parents	+

Notes: References are provided in Supplementary file S2. No intervention was addressed to students.

a Uncontrolled pre–post study.

b (Cluster) randomized controlled trial.

c Only post-intervention evaluation.

d Qualitative or mixed method design.

e Non-randomized controlled trial.

CS, communication strategies; HER, electronic health record; SU, scientific updating; +, encouraging results in favour of the intervention; + −, conflicting results; −, non-significant results.

Six interventions combined EHR changes, performance assessment and feedback, in-person HCP education and actions addressed to patients/parents. Similarly, 2 interventions matched these actions addressed to HCPs with workflow changes or organizational interventions. One intervention focused on presumptive approach. The impact on vaccination rates was mixed.

Five interventions were mainly composed of EHR changes and in-person HCP education, combined with patients/parents’ actions or organizational interventions. All interventions reported a significant vaccination rate increase, except for an intervention not including a component addressed to patients.

Eight interventions included assessment and feedback along with in-person HCP education, educational material or asynchronous learning, all combined with actions for patients/parents. One intervention focused on motivational interviewing. Overall, the vaccination rates improved. One intervention reported significant improvement in attitudes and comfort in counselling.

The remaining interventions were a combination of educational sessions (in-person or online) or materials with actions addressed to patients/parents, changes in the workflow or organizational interventions. One intervention focused on motivational interviewing. Overall, results on vaccination rates were positive, except in two cases.

Lastly, one intervention combined an app supporting HCPs in identification of children who missed vaccinations and organization of scheduling with actions addressed to parents, finding encouraging qualitative results.

## Discussion

This systematic review aimed to map the most recent interventions targeted at HCPs/students to increase vaccine uptake and/or reduce vaccine hesitancy. The final goal was obtaining an overview of available tools to implement an intervention addressed to HCPs within the VAX-TRUST project.

Overall, the intervention components were educational sessions (in-person or online), educational materials, performance assessment and feedback, EHR changes, role play/simulation and online games/apps. Previous reviews highlighted the same categories, except for new technologies, gaming and role play/simulations: apps and new media were used within interventions addressed to patients and works on gaming or simulations were not reported.^15–18^ Also the reviews considering only interventions targeted at HCPs—but focusing on specific vaccines—mainly referred to educational sessions/materials and prompts.^19^^,^^20^ Furthermore, a scattered implementation of digital-based interventions (not only for HCPs) for vaccinations and an inadequate impact assessment are reported across Europe.^21^ Thus, this review showed some potential innovative strategies that should be further evaluated.

Frequently, interventions were multi-component strategies and/or combined with actions for patients. Many works highlighted the most effective interventions to increase vaccination rates were those directly targeted at the population who should get vaccinated^22^ and those consisting of multiple strategies.^17^^,^^18^ This review showed most intervention categories had mixed findings, showing both significant and non-significant results, especially if more outcomes were measured within one domain. No categories showed completely non-significant results across all outcomes, but very few had consistently significant positive results.

Among the multiple-component interventions exclusively addressed to HCPs/students, all interventions combining role play/simulation with in-person education showed significant improvement in knowledge, attitudes/perceptions or confidence, while the same was not true for role play/simulation plus asynchronous learning. This highlights the importance of simulation as tool for supporting HCPs and suggests a possible superiority of the in-person education that should be investigated. However, the studies did not evaluate the long-term effect on the outcomes nor the effect on vaccination rate. Efficacy and effectiveness of these promising strategies should be explored through more adequate evaluations. Conversely, multiple-component interventions not exclusively addressed to HCPs were mainly evaluated through vaccination rates. Interventions with EHR changes and in-person education always reported significant improvement in vaccination rates when combined with actions addressed to patients, as suggested in previous research,^22^ confirming the importance of multilevel interventions directly involving the target population. Interestingly, multi-component interventions with actions not addressed to HCPs did not include role play/simulation elements. Given the encouraging results of interventions only targeted at HCPs, future research should try to integrate these actions within multilevel strategies. Additionally, our review highlighted relevant issues related to evaluation methods, such as study design (often uncontrolled), short follow-up (most studies did not assess long-term effect on vaccination rates nor other outcomes), conflicting results among different outcomes measured within the same domain and impossibility to evaluate the efficacy of each action in multi-component interventions.

Within educational sessions, the topic of communication was almost as frequent as scientific update. Communication importance was also highlighted by the simulation components of many interventions, which were mainly aimed to test communication skills. The SAGE Working Group on Vaccine Hesitancy found that the provision of communication training for HCPs had positive effects on vaccine uptake, while information-based training had generally poor effect on vaccine uptake although increasing the HCPs confidence.^23^ Thus, including communication training in interventions to increase vaccine uptake is essential. However, the effectiveness of communication approaches depends on the message-framing techniques used.^24^ Unfortunately, most articles did not provide details on the contents of the communication training, so future research should delve into the characteristics of the programs to understand if the non-significant results could be partially due to the taught communication methods. When specified, the most frequent communication styles were presumptive and conversational approach, especially motivational interviewing. These approaches are known to positively affect vaccine acceptance.^25^^,^^26^ Braun et al. reported the presumptive approach is the most well documented technique for boosting vaccine uptake, while motivational interviewing is promising but needs additional research.^27^ There is not enough evidence to definitively declare the communication type that should be used.^28^ Nonetheless, keeping examining which methods could be the most effective is crucial as there is an association between recommendation by HCPs and vaccination^29^ and the doctor–patient relationship represents a key for building trust.^5^

This review had some limitations. The great heterogeneity and methodological discrepancies in interventions and outcome measurements precluded a more precise summary through a meta-analysis. The narrative approach employed may limit the insights that can be derived from the results of the interventions. Most interventions were evaluated through uncontrolled studies, thus limiting available evidence and, concerning multi-component interventions, it was not possible to state the weight of each action. Even in the controlled trials, the ‘usual care’ often used in control groups varied between practices so making difficult comparisons across different studies. Nevertheless, this work provided an overview of the interventions targeted at HCPs and students, summarizing the latest data.

## Conclusions

Although there was a gap in innovation, this review found that apps, gaming, role play/simulations could represent potential new categories of interventions to support HCPs and students in increasing vaccinations among patients and improving their knowledge and confidence. Additionally, multi-component interventions including actions for both HCPs and patients had positive results but did not consider role play/simulations, which should be integrated in multilevel interventions in future research. To achieve stronger evidence, this article highlighted the substantial necessity of more robust evaluations with longer follow-up, the use of standardized measurements across studies and the need of studying in depth which communication strategies could be more effective.

## Supplementary Material

ckad134_Supplementary_Data

## Data Availability

All relevant data are within the article and Supplementary files. Apps, gaming, role play and simulations could represent innovative interventions to support healthcare professionals in increasing vaccination rates. Multilevel interventions did not include role play or simulations, which should be integrated in future research. There is the need of more robust evaluations, longer follow-up and standardized measurements across studies.
