# Supplementary material for: Countering vaccine hesitancy: a systematic review of interventions to strengthen healthcare professionals’ action
Source: Eur J Public Health. 2023 Aug 15;33(5):905–15. doi: 10.1093/eurpub/ckad134 (PMC10567238; doi:10.1093/eurpub/ckad134)
Supplement: ckad134_Supplementary_Data [file ckad134_supplementary_data.zip › ckad134_Supplementary_Data/ejph-2023-03-om-0103-File003.pdf]

Additional file 1: List of search terms

Search performed on: 26.01.2022

PUBMED

(Vaccine*[tiab] OR Vaccination*[tiab] OR immuniz*[tiab] OR immunis*[tiab] OR shot*[tiab] OR jab[tiab] OR jabs[tiab] OR “Vaccines”[Mesh] OR “Immunization”[Mesh] OR “Vaccination”[Mesh]) AND (Intent*[tiab] OR willing*[tiab] OR attitud*[tiab] OR hesitanc*[tiab] OR accept*[tiab] OR hesitant[tiab] OR concern*[tiab] OR doubt*[tiab] OR criticis*[tiab] OR sceptic*[tiab] OR fear*[tiab] OR refus*[tiab] OR reject*[tiab] OR consen*[tiab] OR confidence[tiab] OR adherence[tiab] OR complian*[tiab] OR uptake[tiab] OR engagement[tiab] OR perception*[tiab] OR opinion*[tiab] OR belief*[tiab] OR behaviour*[tiab] OR behavior*[tiab] OR choice*[tiab] OR barrier*[tiab] OR religio*[tiab] OR delay[tiab] OR reluctant[tiab] OR comply[tiab] OR “Vaccination Refusal”[Mesh] OR “health communication”[tiab] OR “miscommunication”[tiab] OR "Health Communication"[Mesh]) AND (((health[tiab] OR healthcare[tiab] OR “health care”[tiab] OR medical[tiab] OR clinical[tiab]) AND (personnel [tiab] OR worker*[tiab] OR provider*[tiab] OR workforce*[tiab] OR work-force*[tiab] OR staff[tiab] OR practitioner[tiab] OR practitioners[tiab] OR labor[tiab] OR labour[tiab] OR professional[tiab] OR professionals[tiab] OR trainee[tiab] OR trainees[tiab] OR intern[tiab] OR interns[tiab] OR specialist[tiab] OR “specialists”[tiab] OR student*[tiab])) OR doctor[tiab] OR doctors[tiab] OR clinician[tiab] OR clinicians[tiab] OR physician[tiab] OR physicians[tiab] OR resident[tiab] OR residents[tiab] OR residency[tiab] OR intern[tiab] OR interns[tiab] OR “house officer”[tiab] OR “house officers”[tiab] OR registrar[tiab] OR registrars[tiab] OR “general practitioner*”[tiab] OR pharmacist [tiab] OR pharmacists [tiab] OR nurse [tiab] OR nurses [tiab] OR nursing [tiab] OR nursings [tiab] OR midwife [tiab] OR midwives [tiab] OR midwifery [tiab] OR gynaecologist [tiab] OR gynaecologists [tiab] OR gynecologist [tiab] OR gynecologists [tiab] OR obstetrician [tiab] OR obstetricians [tiab] OR paediatrician [tiab] OR paediatricians [tiab] OR pediatrician [tiab] OR pediatricians [tiab] OR “social worker*”[tiab] OR "Health Personnel"[Mesh] OR “Health Workforce”[Mesh])

Filter: from 2016 (included)

SCOPUS

TITLE-ABS-KEY((Vaccine* OR Vaccination* OR immuniz* OR immunis* OR shot* OR jab OR jabs) AND (Intent* OR willing* OR attitud* OR hesitanc* OR accept* OR hesitant OR concern* OR doubt* OR criticis* OR sceptic* OR fear* OR refus* OR reject* OR consen* OR confidence OR adherence OR complian* OR uptake OR engagement OR perception* OR opinion* OR belief* OR behaviour* OR behavior* OR choice* OR barrier* OR religio* OR delay OR reluctant OR comply OR "health communication" OR "miscommunication") AND (((health OR healthcare OR "health care" OR medical OR clinical) AND (personnel OR worker* OR provider* OR workforce* OR work-force* OR staff OR practitioner OR practitioners OR labor OR labour OR professional OR professionals OR trainee OR trainees OR intern OR interns OR specialist OR "specialists" OR student*)) OR doctor OR doctors OR clinician OR clinicians OR physician OR physicians OR resident OR residents OR residency OR intern OR interns OR "house officer" OR "house officers" OR registrar OR registrars OR "general practitioner*" OR pharmacist OR pharmacists OR nurse OR nurses OR nursing OR nursings OR midwife OR midwives OR midwifery OR gynaecologist OR gynaecologists OR gynecologist OR gynecologists OR obstetrician OR obstetricians OR paediatrician OR paediatricians OR pediatrician OR pediatricians OR "social worker*")) AND (LIMIT-TO (PUBYEAR,2022) OR LIMIT-TO (PUBYEAR,2021) OR LIMIT-TO (PUBYEAR,2020) OR LIMIT-TO (PUBYEAR,2019) OR LIMIT-TO (PUBYEAR,2018) OR LIMIT-TO (PUBYEAR,2017) OR LIMIT-TO (PUBYEAR,2016)

EMBASE

# 1

vaccine*:ab,ti OR vaccination*:ab,ti OR immuniz*:ab,ti OR immunis*:ab,ti OR shot*:ab,ti OR jab:ab,ti OR jabs:ab,ti

#2

intent*:ab,ti OR willing*:ab,ti OR attitud*:ab,ti OR hesitanc*:ab,ti OR accept*:ab,ti OR hesitant:ab,ti OR concern*:ab,ti OR doubt*:ab,ti OR criticis*:ab,ti OR sceptic*:ab,ti OR fear*:ab,ti OR refus*:ab,ti OR reject*:ab,ti OR consen*:ab,ti OR confidence:ab,ti OR adherence:ab,ti OR complian*:ab,ti OR uptake:ab,ti OR engagement:ab,ti OR perception*:ab,ti OR opinion*:ab,ti OR belief*:ab,ti OR behaviour*:ab,ti OR behavior*:ab,ti OR choice*:ab,ti OR barrier*:ab,ti OR religio*:ab,ti OR delay:ab,ti OR reluctant:ab,ti OR comply:ab,ti OR 'health communication':ab,ti OR 'miscommunication':ab,ti

#3

(health:ab,ti OR healthcare:ab,ti OR 'health care':ab,ti OR medical:ab,ti OR clinical:ab,ti) AND (personnel:ab,ti OR worker*:ab,ti OR provider*:ab,ti OR workforce*:ab,ti OR 'work force*':ab,ti OR staff:ab,ti OR practitioner:ab,ti OR practitioners:ab,ti OR labor:ab,ti OR labour:ab,ti OR professional:ab,ti OR professionals:ab,ti OR trainee:ab,ti OR trainees:ab,ti OR intern:ab,ti OR interns:ab,ti OR specialist:ab,ti OR 'specialists':ab,ti OR student*:ab,ti) OR doctor:ab,ti OR doctors:ab,ti OR clinician:ab,ti OR clinicians:ab,ti OR physician:ab,ti OR physicians:ab,ti OR resident:ab,ti OR residents:ab,ti OR residency:ab,ti OR intern:ab,ti OR interns:ab,ti OR 'house officer':ab,ti OR 'house officers':ab,ti OR registrar:ab,ti OR registrars:ab,ti OR 'general practitioner*':ab,ti OR pharmacist:ab,ti OR pharmacists:ab,ti OR nurse:ab,ti OR nurses:ab,ti OR nursing:ab,ti OR nursings:ab,ti OR midwife:ab,ti OR midwives:ab,ti OR midwifery:ab,ti OR gynaecologist:ab,ti OR gynaecologists:ab,ti OR gynecologist:ab,ti OR gynecologists:ab,ti OR obstetrician:ab,ti OR obstetricians:ab,ti OR paediatrician:ab,ti OR paediatricians:ab,ti OR pediatrician:ab,ti OR pediatricians:ab,ti OR 'social worker':ab,ti

#4

#1 AND #2 AND #3

#5

#4 AND (2016:py OR 2017:py OR 2018:py OR 2019:py OR 2020:py OR 2021:py OR 2022:py)

#6

#4 AND (2016:py OR 2017:py OR 2018:py OR 2019:py OR 2020:py OR 2021:py OR 2022:py) AND [embase]/lim NOT ([embase]/lim AND [medline]/lim)
