# Supplementary material for: Countering vaccine hesitancy: a systematic review of interventions to strengthen healthcare professionals’ action
Source: Eur J Public Health. 2023 Aug 15;33(5):905–15. doi: 10.1093/eurpub/ckad134 (PMC10567238; doi:10.1093/eurpub/ckad134)
Supplement: ckad134_Supplementary_Data [file ckad134_supplementary_data.zip › ckad134_Supplementary_Data/ejph-2023-03-om-0103-File004.pdf]

Additional file 2: Supplementary Material

Countering vaccine hesitancy: a systematic review of interventions to strengthen health care professionals' action

Authors
Giuseppina Lo Moro[1]*, Maria Ferrara[2], Elisa Langiano[2], Davide Accortanzo[1], Toni Cappelletti[1], Aldo De Angelis[1], Maurizio Esposito[2], Alessandro Prinzivalli[1], Alessandra Sannella[2], Sara Sbaragli[2], Pia Vuolanto[3], Roberta Siliquini[1,4], Elisabetta De Vito[2]
Affiliations
[1]Department of Public Health Sciences and Paediatrics, University of Turin, Turin, Italy
[2]Department of Human, Social and Health Sciences, University of Cassino and Southern Lazio, Cassino, Italy
[3]Research Centre for Knowledge, Science, Technology and Innovation Studies of Tampere University, Finland
[4]AOU City of Health and Science of Turin, Turin, Italy

***Corresponding author: Giuseppina Lo Moro**
Department of Public Health Sciences and Paediatrics, University of Turin, Turin, Italy
Email: giuseppina.lomoro@unito.it

Table of contents

Table S1. Characteristics of the studies: year of publication, country, study design, and setting 2
Table S2. Characteristics of the studies: participants and incentives to participation 8
Table S3. Characteristics of the studies: primary aims of the intervention 15
Table S4. Characteristics of the studies: year of study, involved vaccination, single or multiple components, length of intervention, control group features 21
Table S5. Characteristics of the studies: funding and conflict of interest 29
Table S6. Risk of bias for non-randomized studies: Critical appraisal Checklist for Quasi-Experimental Studies, Joanna Briggs Institute (JBI) 34
Table S7. Risk of bias for cluster randomized controlled studies: RoB 2 assessment 38
Table S8. Risk of bias for randomized controlled studies: RoB 2 assessment 40
Extended results 41
Details on the main results of the intervention evaluation 57
References 88

Table S1. Characteristics of the studies: year of publication, country, study design, and setting

First Author	Year of publication	Country	Study design (only considering the part of the intervention involving HCPs/students)	Setting
Abdalla A.	2021	United Arab Emirates	pre-post study	Pharmacy
Abdulla E.	2020	Qatar	pre-post study	Primary care practice
Amare A.T.	2021	Ethiopia	mixed method design (non-randomized controlled trial)	Health center
Arogundade L.	2019	Nigeria	mixed method design (only post intervention evaluation)	Routine immunization services
Austin J. D.	2020	USA	mixed method design (only post intervention evaluation)	Federally Qualified Health Center
Barton S.M.	2022	USA	non-randomized controlled trial	University
Bechini A.	2019	Italy	pre-post study	University
Berenson A.B.	2020	USA	pre-post study	University
Berenson A.B.	2020	USA	pre-post study	University
Bishop J.M.	2021	USA	pre-post study	Hospital (pediatric department)
Blake H.	2022	UK (but participants from 26 countries: Algeria, Australia, England, Finland, Ghana, Greece, Guernsey, France, Ireland, India, Indonesia, Italy, Jordan, Lebanon, Malawi, Nigeria, Pakistan, Philippines, Poland, Romania, Scotland, South Africa, Thailand, Uganda,	mixed method design (pre-post study)	Online, no specific setting

		United States of America, Wales.)		
Boey L.	2021	Belgium	pre-post study	Long-term care facility
Bonville C.A.	2019	USA	pre-post study	Pediatric practice
Bradley C.L.	2021	USA	pre-post study	University
Bradley-Ewing A.	2021	USA	cluster RCT	Community-based practices
Bratic J. S.	2019	USA	pre-post study	Pediatric primary care practice
Brewer N.T.	2017	USA	cluster RCT	Primary care practice
Brewer N.T.	2021	USA	non-randomized controlled trial	Primary care practice
Brodie N.	2018	USA	pre-post study	Primary care practice
Buenger L.E.	2020	USA	pre-post study	Emergency department
Casalino E.	2018	France	pre-post study	Emergency department
Cates J.R.	2020	USA	interventional study with only post intervention evaluation	Pediatric and family medicine practice
Cates J.R.	2018	USA	non-randomized controlled trial	Health center
Chamberlain A.T.	2019	USA	interventional study with only post intervention evaluation	Obstetric/maternity care
Chang C.Y.	2021	Taiwan	mixed method design (non-randomized controlled trial)	University
Chase A.J.	2020	USA	qualitative study (only post intervention evaluation)	University
Chen G.	2021	USA	pre-post study	University
Chen H.	2020	China	pre-post study	Hospital
Chidume T.	2020	USA	interventional study with only post intervention evaluation	University
Chin J.	2021	USA	pre-post study	Outpatient allergy and clinical immunology practice
Choi N.	2017	USA	pre-post study	Clinics that participate in the federal Vaccines for Children Program (VFC), which provides vaccines at no purchase cost to clinicians serving children who might not otherwise have access to vaccines
Ciemins E.L.	2020	USA	mixed method design (non-randomized controlled trial)	Health care organization
Cieslowski B.	2020	USA	mixed method design (pre-post study)	Health center
Coleman A.	2017	USA	interventional study with only post intervention evaluation	University
Costello J.	2019	USA	pre-post study	Outpatient pediatric nephrology clinic
Cotter J.C.	2019	USA	pre-post study	University
Dawson R.	2018	USA	pre-post study	Military primary care practice
Dehlinger C.	2021	USA	pre-post study	Women's health ambulatory clinics

Dempsey A.F.	2018	USA	cluster RCT	Family medicine and pediatric primary care practice
Deshmukh U.	2018	USA	pre-post study	hospital-based OB/GYN clinic serving a diverse, low-income patient population
Drainoni M.L.	2021	USA	qualitative study (only post intervention evaluation)	Pediatric and family medicine practice
Dybsand L.L.	2019	USA	mixed method pilot project (interventional study only with post intervention evaluation)	Hospital (pediatric department)
Evans L.	2019	USA	pre-post study	University
Farmar A.M.	2016	USA	interventional study with only post intervention evaluation	Safety-net clinics
Fiks A.G.	2016	USA	non-randomized controlled trial	Pediatric primary care practice
Fiorito T.M.	2021	USA	pre-post study	Hospital (pediatric department)
Fisher-Borne M.	2018	USA	cluster RCT	Safety-net clinics
Frederick K.D.	2020	USA	mixed method design (only post intervention evaluation)	Pharmacy
Gagneur A.	2019	Canada	pre-post study	Public health clinics
Garbutt J.M	2018	USA	mixed method design (only post intervention evaluation)	Primary care practice
Gatwood J.	2021	USA	cluster RCT	Pharmacy
Giduthuri J.G.	2019	India	pilot RCT	Private clinics (antenatal care)
Giles M. L.	2021	Australia	pre-post study	Obstetric/maternity care
Gilkey M.B.	2019	USA	cluster RCT	Children’s Health Care System (not-for-profit integrated delivery system)
Gingold J.A.	2016	USA	mixed method design (pre-post study)	Pediatric primary care practice
Glanternik J.R.	2020	USA	pre-post study	Family and pediatric practice
Hastings T.J.	2019	USA	cluster RCT	Pharmacy
Heaton P.C.	2022	USA	cluster RCT	Pharmacy
Irving S.A.	2018	USA	non-randomized controlled trial	Health center
Jacobs-Wingo J.L.	2017	USA	pre-post study	Indian Health Service
Jina a.	2019	USA	pre-post study	Women’s hospital
Jones K.M.	2016	USA	pre-post study	Online, no specific setting
Kaufman J.	2020	Australia	feasibility and acceptability pilot study (no pre-post assessment nor control groups)	Women’s hospital
Kawczak S.	2020	USA	non-randomized controlled trial	Primary care practice
Kepka D.	2021	USA	pre-post study	Rural medical clinic
Kim R.H.	2018	USA	non-randomized controlled trial	Primary care practice
Koski K.	2018	Finland	qualitative study (only post intervention evaluation)	University

Krishnaswamy S.	2018	Australia	non-randomized controlled trial	Obstetric/maternity care
Kumar M.M.	2019	USA	pre-post study	Pediatric practice
Leila R.A.	2021	Bahrain	pre-post study	Primary care practice
Lepiller Q.	2020	France	pre-post study	University
Lin C.	2016	USA	cluster RCT	Primary care practice
Lin J.L	2018	USA	non-randomized controlled trial	Pharmacy
Loiacono M.M.	2021	USA	cluster RCT	Pharmacy
Malo T.L.	2018	USA	cluster RCT	Primary care practice
Malone K.	2016	USA	pre-post study	Division of Nephrology
Marchand-Ciriello L.	2020	USA	pre-post study	Pediatric practice
Marotta C.	2017	Italy	pre-post study	University
Maurici M.	2019	Italy	pre-post study	Centers of the Immunization Service
Mazzoni S.E.	2016	USA	pre-post study	Underserved outpatient ob-gyn clinics
McFadden S.M.	2021	USA	pre-post study	Online, no specific setting
McGaffey A.	2019	USA	pre-post study	health center
McLean H.Q.	2017	USA	non-randomized controlled trial	Regional health care system
Mitchell G.	2021	UK (Northern Ireland)	pre-post study	University
Morhardt T.	2016	USA	mixed method design (pre-post study)	Residency program in pediatrics
Nissen M.	2019	USA	pre-post study	Primary care practice
Nold L.	2020	USA	qualitative study (only post intervention evaluation)	University
Nowalk M.P.	2017	USA	pre-post study (secondary analysis of a cluster RCT)	Primary care practice
O’Donnell M.	2018	USA	pre-post study	Primary care practice
O’Leary S.T.	2019	USA	cluster RCT	Children Hospital
Oliver K.	2020	USA	pre-post study	Pediatric practice
Olshefski R.S.	2018	USA	pre-post study	Children Hospital
Onello E.	2020	USA	pre-post study	University
Orefice R.	2019	Australia	pre-post study	Hospital for Women and Children
Pahud B.	2020	USA	cluster RCT	Residency programs (pediatric, family medicine, and medicine-pediatric)
Pampena E.	2019	USA	pre-post study	Oral health practice

Patel M.S.	2017	USA	non-randomized controlled trial	Internal medicine departments
Percy J.N	2019	USA	mixed method design (pre-post study)	Pharmacy
Perkins R.B.	2020	USA	mixed method design (pre-post study)	Family medicine and pediatric primary care practice
Perkins R.B.	2020	USA	stepped-wedge cluster randomized trial	Clinical sites serving primarily low-income and minority patients
Persell S.D.	2020	USA	pre-post study	Primary care practice
Rand C.M.	2018	USA	pre-post study	Pediatric primary care practice
Rand C.M.	2018	USA	pre-post study	Community practices and pediatric continuity clinics
Rao S.	2020	USA	pre-post study	Children Hospital
Real F.J.	2021	USA	qualitative study (only post intervention evaluation)	Hospital (pediatric department)
Real F.J.	2017	USA	non-randomized controlled trial	Pediatric primary care practice
Reno J.E.	2018	USA	pre-post study (secondary analysis of a cluster RCT)	Family medicine and pediatric primary care practice
Reno J.E.	2018	USA	pre-post study (secondary analysis of a cluster RCT)	Pediatric and family medicine practice
Rosen B.L.	2021	USA	qualitative study (only post intervention evaluation)	Pediatric Hospital
Salous M.H.	2020	USA	pre-post study	Indian Health Service
Sanderson M.	2017	USA	non-randomized controlled trial	Safety-net clinics
Sandokji I.	2021	USA	pre-post study	Nephrology clinic
Schnaith A.M.	2018	USA	pre-post study	University
Serino L.	2020	Italy	interventional study with only post intervention evaluation	Local Health Department
Shukla A.	2018	USA	interventional study with only post intervention evaluation	Oral health practice
Skoy E.	2020	USA	pre-post study	Pharmacy
Spelman J. F.	2022	USA	interventional study with only post intervention evaluation	Veteran care
Spina C.I.	2020	USA	pre-post study	Obstetric/maternity care
Srirangan K.	2021	Canada	mixed method design (only post intervention evaluation)	Pharmacy
Steiner C.R.	2021	USA	pre-post study	Nurse-run walk-in immunization clinic
Stetson R.C.	2019	USA	pre-post study	Pediatric Hospital
Suryadevara M.	2019	USA	pre-post study	Pediatric practice
Szilagyi P.G.	2021	USA	cluster RCT	Pediatric primary care practice
Tchoualeu D.D.	2021	Ghana	pre-post study	Health district
Torabizadeh C.	2020	Iran	pre-post study	University
Traicoff D.	2021	Ghana	interventional study with only post intervention evaluation	Health district

Vinci D.M.	2021	USA	pre-post study	Pediatric practice
Visalli G.	2021	Italy	pre-post study	University
Vyas D.	2018	USA	pre-post study	University
Wallace-Brodeur R.	2020	USA	pre-post study	Local Health Department
Werk L.N.	2019	USA	cluster RCT	Primary care practice
Wermers R.	2021	USA	pre-post study	University
Whitaker J.A.	2018	USA	cluster RCT	Hospital (internal medicine department) and primary care services of internal medicine
Wiley R.	2019	USA	pre-post study	University
Wilkinson T.A.	2019	USA	RCT	Primary care practice (underserved and minority population)
Williams S.E.	2021	USA	stepped-wedge cluster randomized trial	Pediatric practice
Zaidi S.	2020	Pakistan	qualitative study (only post intervention evaluation)	Underserved rural district
Zimet G.	2017	USA	cluster RCT	Pediatric practice
Zimmerman R.K.	2017	USA	cluster RCT	Family medicine and pediatric primary care practice

Table S2. Characteristics of the studies: participants and incentives to participation

First Author	Year of publication	Students or Healthcare Professionals (HCPs) or Residents	Participants & sample size (only considering the part of the intervention involving HCPs/students)	Incentives to participation
Abdalla A.	2021	HCPs	16 hospital and community pharmacists.	credits
Abdulla E.	2020	HCPS	120 nurses	.
Amare A.T.	2021	HCPs	Intervention: 15 sites (40 HCPs)<br>Control: 15 sites (50 HCPs)	.
Arogundade L.	2019	HCPs	117 HCPs providing routine immunization services and tutors serving in pre-service health training institutions (90 HCPs and 27 tutors)	.
Austin J. D.	2020	HCPs	57 HCPs(physicians, medical assistants, nurses, and various administrative staff)	.
Barton S.M.	2022	residents	Intervention: 27 pediatric residents<br>Control: 26 pediatric residents	credits
Bechini A.	2019	students	100 medical students	.
Berenson A.B.	2020	students	512 medical students<br>388 nursing students	small gift + curricular lessons/part of hospital's educational lecture series-meetings
Berenson A.B.	2020	students	256 medical students	curricular lessons/part of hospital's educational lecture series-meetings
Bishop J.M.	2021	residents	13 pediatric residents	economic incentive (personal level) + credits
Blake H.	2022	HCPs	survey, n = 162 HCPs (health and social care professionals and trainees, or public health specialists.)<br>qualitative interviews, n = 17 (nurses (n = 12), social scientists (n = 2), occupational health specialists (n = 1) and COVID-19 vaccinators (n = 2))	.
Boey L.	2021	HCPs	340 HCPs	.
Bonville C.A.	2019	HCPs	Pilot: 5 pediatric practices (each employing between 4 and 10 providers)<br>Study: 8 pediatric practices (each employing between 1 and 10 providers)<br>Each practice designated one physician provider and one staff member to serve in the role of QI champion	.
Bradley C.L.	2021	students	Fall 2018: 49 pharmacy students<br>Fall 2019: 46 pharmacy students	curricular lessons/part of hospital's educational lecture series-meetings
Bradley-Ewing A.	2021	HCPs	Combined intervention: 2 practices<br>Single intervention (described as "control"): 2 practices	.

Bratic J. S.	2019	HCPs	Clinical staff (including registered nurses, licensed vocational nurses, and medical assistants) and Providers (including physicians, fellows, residents, nurse practitioners, and physician assistants)	.
Brewer N.T.	2017	HCPs	Intervention 1 (announcement training): 10 practices<br>Intervention 2 (conversation training): 10 practices<br>Control: 10 practices	.
Brewer N.T.	2021	HCPs	Intervention: one health system (reached 234 physicians, nurses, and other clinic staff)<br>Control: one health system	credits
Brodie N.	2018	HCPs	132 HCPs	.
Buenger L.E.	2020	HCPs	One emergency department (ED)	.
Casalino E.	2018	HCPs	One emergency department (ED)	.
Cates J.R.	2020	HCPs	113 HCPs (69 completed an evaluation)	credits
Cates J.R.	2018	HCPs	Intervention: 14 practices<br>Control: 161 practices	economic incentive (practice and personal level)
Chamberlain A.T.	2019	HCPs	62 obstetric care providers	credits
Chang C.Y.	2021	students	Intervention: 18 nursing students<br>Control: 18 nursing students	curricular lessons/part of hospital's educational lecture series-meetings
Chase A.J.	2020	students	160 medical students	.
Chen G.	2021	students	55 medical students	.
Chen H.	2020	HCPs	1354 HCPs	.
Chidume T.	2020	students	49 nursing students	curricular lessons/part of hospital's educational lecture series-meetings
Chin J.	2021	HCPs	4 providers of 1 site	.
Choi N.	2017	HCPs	80 sites	.
Ciemins E.L.	2020	HCPs	Intervention: 7 health care organizations<br>Control: 27 health care organizations	.
Cieslowski B.	2020	HCPs	12 nursing staff	.
Coleman A.	2017	students	approximately 120 medical students per year	curricular lessons/part of hospital's educational lecture series-meetings
Costello J.	2019	HCPs	Providers of one outpatient pediatric nephrology clinic	.
Cotter J.C.	2019	students	37 dental hygiene students	.
Dawson R.	2018	HCPs	200 HCPs	curricular lessons/part of hospital's educational lecture series-meetings
Dehlinger C.	2021	HCPs	3 clinics	.

Dempsey A.F.	2018	HCPs	Intervention: 8 sites<br>Control: 8 sites<br>(total: 188 medical professionals)	.
Deshmukh U.	2018	HCPs	Staff of one ob-gyn hospital-based practice	.
Drainoni M.L.	2021	HCPs	12 primary care providers (PCP), 5 nurses, and 9 individuals with dual leadership and PCP roles	curricular lessons/part of hospital's educational lecture series-meetings
Dybsand L.L.	2019	HCPs	5 pediatric providers	.
Evans L.	2019	students+residents	333 students, physician assistant students, graduate students in public health, and family medicine residents	.
Farmar A.M.	2016	HCPs	safety net–integrated health system	.
Fiks A.G.	2016	HCPs	Intervention: 27 pediatricians<br>Control: 200 pediatricians and nurses	credits
Fiorito T.M.	2021	students+residents	25 pediatric residents<br>3 medical students	curricular lessons/part of hospital's educational lecture series-meetings
Fisher-Borne M.	2018	HCPs	Intervention 1 (Grant funded $90000): 10 systems (57 sites)<br>Intervention 2 (Grant funded $10000): 10 systems (37 sites)<br>Intervention 3 (Technical assistance): 10 systems (36 sites)	.
Frederick K.D.	2020	HCPs	1128 pharmacists	.
Gagneur A.	2019	HCPs	34 immunization nurses	credits
Garbutt J.M	2018	HCPs	Practice-based research network of community pediatricians and pediatric nurses	economic incentive (personal level)
Gatwood J.	2021	HCPs	2 sites with different interventions:<br>At Nashville:<br>Control (no training): 26 pharmacists<br>Online training only (partial training): 20 pharmacists<br>At Memphis:<br>Control (no training): 25 pharmacists<br>Online plus in-person simulation training (full training): 25 pharmacists	.
Giduthuri J.G.	2019	HCPs	Slum communities:<br>Intervention: 11 clinicians<br>Control: 5 clinicians<br>Middle-class communities:<br>Intervention: 5 clinicians<br>Control: 9 clinicians	.
Giles M. L.	2021	HCPs	6 maternity services	.
Gilkey M.B.	2019	HCPs	Intervention: 13 clinics (42 physicians)<br>Control: 12 clinics (35 physicians)	credits
Gingold J.A.	2016	HCPs	16 primary care pediatric practices	credits
Glanternik J.R.	2020	HCPs	249 pediatricians and family practitioners	.

Hastings T.J.	2019	HCPs	Intervention: 37 pharmacists/pharmacy technicians<br>Control: 30 pharmacists/pharmacy technicians	credits
Heaton P.C.	2022	HCPs	Intervention: 244 pharmacy stores<br>Control: 257 pharmacy stores	.
Irving S.A.	2018	HCPs	Intervention: 9 clinics<br>Control: 3 clinics	.
Jacobs-Wingo J.L.	2017	HCPs	10 facilities	.
Jina a.	2019	HCPs	>50 obstetricians	.
Jones K.M.	2016	HCPs	151 American College of Obstetricians and Gynecologists (ACOG) members	.
Kaufman J.	2020	HCPs	25 midwives	.
Kawczak S.	2020	HCPs	Intervention: 100 physicians<br>Control: 198 physicians	credits
Kepka D.	2021	HCPs	One clinic	economic incentive (personal level)
Kim R.H.	2018	HCPs	Participants: primary care physicians (PCPs)-> medical assistants<br>Intervention: 3 internal medicine sites<br>Control: 8 internal medicine and family medicine sites	.
Koski K.	2018	students	9 medical students	curricular lessons/part of hospital's educational lecture series-meetings
Krishnaswamy S.	2018	HCPs	3 arms: one hospital per intervention	.
Kumar M.M.	2019	HCPs	96 HCPs	.
Leila R.A.	2021	HCPs	11 doctors and 14 nurses	.
Lepiller Q.	2020	students	530 students (nursing, medicine, physiotherapy, pharmacy, midwifery)	curricular lessons/part of hospital's educational lecture series-meetings
Lin C.	2016	HCPs	Intervention: 13 practices<br>Control: 12 practices	.
Lin J.L	2018	HCPs	Intervention (wholestaff training): 4 pharmacies<br>Control (train-the-trainer approach): 4 pharmacies	.
Loiacono M.M.	2021	HCPs	Intervention: 2292 pharmacies<br>Control: 2297 pharmacies	.
Malo T.L.	2018	HCPs	83 vaccine-prescribing and 59 non-vaccine prescribing clinicians	economic incentive (practice and personal level) + credits
Malone K.	2016	HCPs	One Division of nephrology	.
Marchand-Ciriello L.	2020	HCPs	13 pediatric providers (pediatricians and nurses)	.
Marotta C.	2017	students	118 students (medicine, biology, pharmacy, nursing, health assistance)	curricular lessons/part of hospital's educational lecture series-meetings

Maurici M.	2019	HCPs	9 medical doctors and 11 nurses	.
Mazzoni S.E.	2016	HCPs	2 community-based ob-gyn clinics	.
McFadden S.M.	2021	HCPs	202 providers (physicians, nurses, physician assistants, and medical assistants)	economic incentive (personal level) + credits
McGaffey A.	2019	HCPs	Entire health center medical and non-medical staff	.
McLean H.Q.	2017	HCPs	Intervention: 6 pediatric departments and 3 family practice/other<br>Control: 34 departments (all other departments that provide primary care and vaccinations to adolescents)	credits
Mitchell G.	2021	students	356 nursing students	.
Morhardt T.	2016	residents	26 residents in pediatrics	.
Nissen M.	2019	HCPs	7 family medicine clinics in year one, 32 primary care clinics in year two	.
Nold L.	2020	students	>200 nursing students	curricular lessons/part of hospital's educational lecture series-meetings
Nowalk M.P.	2017	HCPs	24 sites	.
O’Donnell M.	2018	HCPs	Professional nurses	.
O’Leary S.T.	2019	HCPs	Intervention: 4 practices (median number of providers 4, min-max: 2–19.4)<br>Control: 4 practices (median number of providers 6, min-max 5–9.2)	.
Oliver K.	2020	HCPs	42 HCPs (pediatricians, nurses, and office staff)	credits
Olshefski R.S.	2018	HCPs	One pediatric hospital (oncology department)	.
Onello E.	2020	students	178 medical students	.
Orefice R.	2019	HCPs	One hospital	.
Pahud B.	2020	residents	Intervention: 734 residencies (total of 621 participants, only 126 completed both pre and post surveys)<br>Control: 710 residencies (total of 609 participants, only 138 completed both pre and post surveys)	.
Pampena E.	2019	HCPs	263 Oral Health Providers	.
Patel M.S.	2017	HCPs	Intervention: 3 internal medicine practices<br>Control: 2 similar internal medicine practices	.
Percy J.N	2019	HCPs	23 pharmacist-extenders (20 pharmacy technicians and 3 pharmacy interns)	.
Perkins R.B.	2020	HCPs	23 HCPs	.
Perkins R.B.	2020	HCPs	5 clinical sites	curricular lessons/part of hospital's educational lecture series-meetings
Persell S.D.	2020	HCPs	approximately 267–298 eligible clinicians per season.	.
Rand C.M.	2018	HCPs	8 practices	economic incentive (practice level) + credits
Rand C.M.	2018	HCPs	33 community practices and 14 pediatric continuity clinics	economic incentive (practice level) + credits

Rao S.	2020	HCPs	1 site: Medical (including residents) and nursing providers caring for patients aged ≥ 6 months of age on the general and subspecialty medical pediatric inpatient units	.
Real F.J.	2021	residents	15 pediatric residents	.
Real F.J.	2017	residents	Intervention: 24 pediatric residents<br>Control: 21 pediatric residents	.
Reno J.E.	2018	HCPs	108 HCPs (nurses and physicians)	.
Reno J.E.	2018	HCPs	46 HCPs	.
Rosen B.L.	2021	HCPs	16 school nurses and school-based health center staff	.
Salous M.H.	2020	HCPs	122 oral health professionals (dentists, dental hygienists, dental therapists, and dental assistants)	.
Sanderson M.	2017	HCPs	Intervention: 2 sites<br>Control: 2 sites	.
Sandokji I.	2021	HCPs	1 nephrologic clinic	.
Schnaith A.M.	2018	students	101 medical students	.
Serino L.	2020	HCPs	142 HCPs (136 completed the questionnaires)	credits
Shukla A.	2018	HCPs	89 Oral health providers (dentists and dental hygienists)	.
Skoy E.	2020	HCPs	282 pharmacists	.
Spelman J. F.	2022	HCPs	8 primary care sites (80 providers)	.
Spina C.I.	2020	HCPs	11 practices	credits
Srirangan K.	2021	HCPs	57 pharmacists	credits
Steiner C.R.	2021	HCPs	All clinic staff (n = 8)	.
Stetson R.C.	2019	HCPs	139 HCPs	.
Suryadevara M.	2019	HCPs	6 pediatric practices (46 providers and nurses)	.
Szilagyi P.G.	2021	HCPs	Intervention: 24 practices (188 clinicians)<br>Control: 24 practices (177 clinicians)	.
Tchoualeu D.D.	2021	HCPs	102 HCPs	.
Torabizadeh C.	2020	HCPs+students	155 HCPs<br>95 nursing students	curricular lessons/part of hospital's educational lecture series-meetings
Traicoff D.	2021	HCPs	74 HCPs	.
Vinci D.M.	2021	HCPs	9 pediatric clinics (199 medical assistants and office staff members)	.
Visalli G.	2021	students	35 obstetrics students	.
Vyas D.	2018	students	180 pharmacy students	curricular lessons/part of hospital's educational lecture series-meetings
Wallace-Brodeur R.	2020	HCPs	264 clinics	.

Werk L.N.	2019	HCPs	4 arms:<br>Control: 4 clinicians (physicians and advanced practice nurses)<br>Computerized clinical decision support system (CCDSS): 4 clinicians (physicians and advanced practice nurses)<br>Web-based training (WBT): 2 clinicians (physicians and advanced practice nurses)<br>CCDSS and WBT (BOTH): 3 clinicians (physicians and advanced practice nurses)	.
Wermers R.	2021	HCPS	19 HCPs (nurses and physicians)	.
Whitaker J.A.	2018	residents	Intervention: 47 internal medicine residents<br>Control: 52 internal medicine residents	.
Wiley R.	2019	students	61 medical students	.
Wilkinson T.A.	2019	HCPs	Intervention: 15 pediatric clinicians<br>Control: 14 pediatric clinicians	.
Williams S.E.	2021	HCPs	8 practices	.
Zaidi S.	2020	HCPs	26 vaccinators	.
Zimet G.	2017	HCPs	Control: 10 pediatric HCPs (pediatricians, family physicians, and nurse practitioners)<br>Intervention 1 (simple prompt): 8 pediatric HCPs (pediatricians, family physicians, and nurse practitioners)<br>Intervention 2 (elaborated prompt): 11 pediatric HCPs (pediatricians, family physicians, and nurse practitioners)	.
Zimmerman R.K.	2017	HCPs	Intervention: 9 practices<br>Control: 11 practices	.

Table S3. Characteristics of the studies: primary aims of the intervention

First Author	Year of publication	Increasing knowledge of HCPs/ students	Improving attitudes/ behaviors / perceptions/ awareness of HCPs/students	Improving confidence/ self-efficacy in communication/ counselling	Improving communication skills- ability to promote vaccination	Reducing missed opportunities	Increasing vaccination rates/uptake in patients	Evaluating acceptability/ satisfaction/ perception that the intervention is helpful	Developing and testing the intervention	Confidence in making recommendations/decision making or comfort with immunization technique or following recommendations	Other
Abdalla A.	2021	X	X	.	.	.	.	.	.	.	.
Abdulla E.	2020	.	.	.	.	.	.	.	.	X	.
Amare A.T.	2021	.	.	.	.	.	X	.	.	.	.
Arogundade L.	2019	X	.	.	.	.	.	.	.	.	.
Austin J. D.	2020	.	.	.	X	.	.	.	.	.	.
Barton S.M.	2022	.	.	.	X	.	.	.	.	.	.
Bechini A.	2019	X	.	.	.	.	.	.	.	.	.
Berenson A.B.	2020	X	X	.	.	.	.	.	.	.	.
Berenson A.B.	2020	.	X	X	.	.	.	.	.	.	.
Bishop J.M.	2021	X	X	X	.	.	.	.	.	.	.
Blake H.	2022	.	.	.	.	.	.	.	X	.	.
Boey L.	2021	.	X	.	.	.	X	.	.	.	.
Bonville C.A.	2019	.	.	.	.	.	X	.	.	.	.
Bradley C.L.	2021	X	.	.	.	.	.	.	.	X	.
Bradley-Ewing A.	2021	.	.	.	.	.	X	X	.	.	.
Bratic J. S.	2019	.	.	.	.	.	X	.	.	.	.
Brewer N.T.	2017	.	.	.	.	.	X	.	.	.	.
Brewer N.T.	2021	.	.	.	X	.	.	.	.	.	.

Brodie N.	2018	.	.	.	X	X	X	.	.	.	.
Buenger L.E.	2020	.	.	.	.	.	X	.	.	.	.
Casalino E.	2018	.	.	.	.	.	X	.	.	.	.
Cates J.R.	2020	X	.	X	.	.	.	.	.	.	offer systems level strategies
Cates J.R.	2018	.	.	.	X	.	X	.	.	.	.
Chamberlain A.T.	2019	.	.	.	X	.	.	.	.	.	.
Chang C.Y.	2021	X	.	.	.	.	.	.	.	.	.
Chase A.J.	2020	.	.	.	X	.	.	.	.	.	.
Chen G.	2021	X	.	.	X	.	.	.	.	.	.
Chen H.	2020	X	.	.	.	.	.	.	.	.	.
Chidume T.	2020	.	.	X	.	.	.	.	.	.	.
Chin J.	2021	.	.	.	.	.	X	.	.	.	improve patient education and knowledge
Choi N.	2017	.	.	.	.	.	X	.	.	.	.
Ciemins E.L.	2020	.	.	.	.	.	X	.	.	.	.
Cieslowski B.	2020	.	.	.	.	.	.	.	.	X	.
Coleman A.	2017	X	X	X	.	.	.	.	.	.	.
Costello J.	2019	.	.	.	.	.	.	.	.	X	.
Cotter J.C.	2019	X	X	X	.	.	.	.	.	.	.
Dawson R.	2018	X	X	.	.	.	X	.	.	.	.
Dehlinger C.	2021	.	.	.	.	.	X	.	.	.	.
Dempsey A.F.	2018	.	.	.	X	.	X	.	.	.	.
Deshmukh U.	2018	.	.	.	.	X	X	.	.	.	.
Drainoni M.L.	2021	X	.	.	X	.	X	.	.	.	.
Dybsand L.L.	2019	.	.	X	.	.	.	.	.	.	.
Evans L.	2019	X	X	.	.	.	.	.	.	.	.
Farmar A.M.	2016	.	.	.	.	.	X	.	.	.	.

Fiks A.G.	2016	.	.	.	.	X	.	.	.	.	.
Fiorito T.M.	2021	X	.	X	X	.	.	.	.	.	.
Fisher-Borne M.	2018	.	.	.	.	.	X	.	.	.	.
Frederick K.D.	2020	.	.	.	.	.	X	.	.	.	.
Gagneur A.	2019	.	.	.	X	.	.	.	X	.	.
Garbutt J.M	2018	.	.	.	.	.	.	.	.	X	.
Gatwood J.	2021	.	.	.	X	.	.	.	.	.	.
Giduthuri J.G.	2019	.	.	.	.	X	.	.	.	.	.
Giles M. L.	2021	.	.	.	.	.	X	.	.	.	.
Gilkey M.B.	2019	.	.	.	.	.	X	.	.	.	.
Gingold J.A.	2016	.	.	.	.	.	X	.	.	.	.
Glanternik J.R.	2020	.	.	X	X	.	.	.	.	.	.
Hastings T.J.	2019	.	.	.	.	.	.	.	.	X	.
Heaton P.C.	2022	.	.	.	.	.	.	.	.	.	to allow health care providers to review immunization records from IIS (immunization information systems) and identify a patient’s recommended vaccinations (> increase vaccination rates)
Irving S.A.	2018	.	.	.	.	X	X	.	.	.	.
Jacobs-Wingo J.L.	2017	.	.	.	.	.	X	.	.	.	.
Jina a.	2019	.	.	.	.	.	X	.	.	.	.

Jones K.M.	2016	.	.	.	.	.	.	.	.	X	.
Kaufman J.	2020	.	.	.	.	.	.	X	.	.	.
Kawczak S.	2020	.	.	.	.	.	X	.	.	.	.
Kepka D.	2021	.	.	.	.	X	X	.	.	.	.
Kim R.H.	2018	.	.	.	.	.	X	.	.	.	.
Koski K.	2018	.	X	.	X	.	.	.	.	.	.
Krishnaswamy S.	2018	.	.	.	.	.	X	.	.	.	.
Kumar M.M.	2019	X	X	X	.	.	.	.	.	.	.
Leila R.A.	2021	.	.	.	X	.	.	.	.	.	.
Lepiller Q.	2020	.	X	.	.	.	.	.	.	.	.
Lin C.	2016	.	.	.	.	X	X	.	.	.	.
Lin J.L	2018	.	.	.	.	.	X	.	.	.	.
Loiacono M.M.	2021	.	X	.	.	.	.	.	.	.	.
Malo T.L.	2018	.	X	.	.	.	.	X	.	.	.
Malone K.	2016	.	.	.	.	X	X	.	.	.	.
Marchand-Ciriello L.	2020	.	.	.	.	.	X	.	.	.	.
Marotta C.	2017	X	X	.	.	.	.	.	.	.	.
Maurici M.	2019	.	.	.	X	.	.	.	.	.	increase in empathy
Mazzoni S.E.	2016	.	.	.	.	.	X	.	.	.	.
McFadden S.M.	2021	.	.	X	.	.	.	.	.	.	.
McGaffey A.	2019	.	.	.	.	.	X	.	.	.	.
McLean H.Q.	2017	.	.	.	.	.	X	.	.	.	.
Mitchell G.	2021	X	X	.	.	.	X	.	.	.	.
Morhardt T.	2016	.	.	X	.	.	.	.	.	.	.
Nissen M.	2019	.	.	.	.	.	X	.	.	.	.
Nold L.	2020	.	.	.	.	.	.	.	X	.	.
Nowalk M.P.	2017	.	.	.	.	.	X	.	.	.	.
O’Donnell M.	2018	X	.	.	.	.	.	.	.	.	.
O’Leary S.T.	2019	.	.	.	.	.	X	.	.	.	.

Oliver K.	2020	X	.	X	.	.	X	.	.	.	.
Olshefski R.S.	2018	.	.	.	.	.	X	.	.	.	.
Onello E.	2020	X	X	.	.	.	.	.	.	.	.
Orefice R.	2019	.	.	.	.	.	X	.	.	.	.
Pahud B.	2020	X	X	X	.	.	.	.	.	.	.
Pampena E.	2019	X	X	.	.	.	.	.	.	.	.
Patel M.S.	2017	.	.	.	.	.	X	.	.	.	.
Percy J.N	2019	.	.	.	.	.	X	.	.	.	.
Perkins R.B.	2020	.	.	.	X	.	.	.	.	.	.
Perkins R.B.	2020	.	.	.	.	.	X	.	.	.	.
Persell S.D.	2020	.	.	.	.	.	X	.	.	.	.
Rand C.M.	2018	.	.	.	.	.	X	.	.	.	.
Rand C.M.	2018	.	.	.	.	X	.	.	.	.	.
Rao S.	2020	.	.	.	.	.	X	.	.	.	.
Real F.J.	2021	X	.	.	X	.	.	.	.	.	.
Real F.J.	2017	.	.	.	X	.	.	.	.	.	.
Reno J.E.	2018	.	.	.	.	.	.	X	.	.	.
Reno J.E.	2018	.	.	.	.	.	.	X	.	.	.
Rosen B.L.	2021	.	.	.	X	.	X	.	.	.	.
Salous M.H.	2020	X	.	X	X	.	.	.	.	.	.
Sanderson M.	2017	.	.	.	.	.	X	.	.	.	.
Sandokji I.	2021	.	X	.	X	.	.	.	.	.	.
Schnaith A.M.	2018	.	.	X	.	.	.	.	.	.	.
Serino L.	2020	X	X	.	.	.	.	.	.	.	.
Shukla A.	2018	X	X	X	.	.	.	.	.	.	.
Skoy E.	2020	.	.	.	.	.	X	.	.	.	.
Spelman J. F.	2022	.	.	.	.	.	X	.	.	.	.
Spina C.I.	2020	.	.	.	.	.	X	.	.	.	.
Srirangan K.	2021	.	.	X	.	.	.	.	.	X	.
Steiner C.R.	2021	X	.	.	X	.	X	.	.	.	.

Stetson R.C.	2019	.	.	.	.	.	X	.	.	.	.
Suryadevara M.	2019	.	X	.	.	.	X	.	.	.	.
Szilagyi P.G.	2021	.	.	.	.	X	X	.	.	.	.
Tchoualeu D.D.	2021	.	.	.	.	.	.	.	.	X	.
Torabizadeh C.	2020	X	X	.	.	.	.	.	.	.	.
Traicoff D.	2021	.	.	.	.	.	.	.	.	X	.
Vinci D.M.	2021	.	.	.	.	.	X	.	.	.	.
Visalli G.	2021	X	.	.	.	.	.	.	.	.	.
Vyas D.	2018	X	.	.	X	.	.	.	.	.	.
Wallace-Brodeur R.	2020	.	.	.	.	X	.	.	.	.	.
Werk L.N.	2019	.	.	.	.	.	X	.	.	.	.
Wermers R.	2021	.	.	.	X	.	.	.	.	.	.
Whitaker J.A.	2018	X	X	.	.	.	X	.	.	.	.
Wiley R.	2019	X	X	.	X	.	.	.	.	.	.
Wilkinson T.A.	2019	.	.	.	.	.	X	.	.	.	.
Williams S.E.	2021	.	.	.	.	.	X	.	.	.	.
Zaidi S.	2020	.	.	.	.	.	.	X	.	.	.
Zimet G.	2017	.	.	.	.	.	X	.	.	.	.
Zimmerman R.K.	2017	.	.	.	.	.	X	.	.	.	.

Table S4. Characteristics of the studies: year of study, involved vaccination, single or multiple components, length of intervention, control group features

First Author	Year of publ.	Year of study	Involved vaccinations	Single or multiple components	Length of intervention	Control group (if present)
Abdalla A.	2021	2020	Influenza	Multiple-component interventions addressed exclusively at HCPs or students	half day	.
Abdulla E.	2020	2018	vaccines in general	Single-component interventions	NA	.
Amare A.T.	2021	2018-2019	vaccines in general	Single-component interventions	NA	No intervention
Arogundade L.	2019	NA	vaccines in general	Single-component interventions	NA	.
Austin J. D.	2020	2016-2018	HPV	Multiple-component interventions addressed exclusively at HCPs or students	1 hour	.
Barton S.M.	2022	2019-2020	vaccines in general	Multiple-component interventions addressed exclusively at HCPs or students	2 hours	Only live training (no AIMS)
Bechini A.	2019	2018	vaccines in general	Single-component interventions	8 hours	.
Berenson A.B.	2020	2016-2020	HPV	Single-component interventions	45 minutes	.
Berenson A.B.	2020	2016-2018	HPV	Single-component interventions	45 minutes	.
Bishop J.M.	2021	2019	HPV	Multiple-component interventions addressed exclusively at HCPs or students	approximately 90 minutes	.
Blake H.	2022	2021	COVID-19	Single-component interventions	NA	.
Boey L.	2021	2017-2018	Influenza	Multiple-component interventions addressed not exclusively at HCPs or students	NA	.
Bonville C.A.	2019	2016-2018	HPV	Multiple-component interventions addressed exclusively at HCPs or students	6 months	.
Bradley C.L.	2021	2018-2019	vaccines in general	Single-component interventions	8 hours	.
Bradley-Ewing A.	2021	2018-2019	HPV	Multiple-component interventions addressed exclusively at HCPs or students	NA	Only assessment and feedback

Bratic J. S.	2019	2014-2017	Influenza	Multiple-component interventions addressed exclusively at HCPs or students	approximately 15 months	.
Brewer N.T.	2017	2015	HPV,Tdap, meningococcal conjugate	Single-component interventions	1 hour	Intervention 2: Conversation training Control: Waitlist
Brewer N.T.	2021	2019	HPV	Multiple-component interventions addressed exclusively at HCPs or students	3 hours	Sending reminders to patients overdue for second or third doses of HPV vaccine
Brodie N.	2018	2014-2015	HPV	Multiple-component interventions addressed exclusively at HCPs or students	approximately 1 year	.
Buenger L.E.	2020	2013-2016	Influenza	Multiple-component interventions addressed exclusively at HCPs or students	7 months	.
Casalino E.	2018	2013-2017	Influenza	Multiple-component interventions addressed not exclusively at HCPs or students	NA	.
Cates J.R.	2020	2015-2017	HPV	Single-component interventions	minimum of 2 hours and up to 12 hours	.
Cates J.R.	2018	2015-2016	HPV	Multiple-component interventions addressed not exclusively at HCPs or students	NA	No intervention
Chamberlain A.T.	2019	2017	vaccines in general	Single-component interventions	1 hour	.
Chang C.Y.	2021	NA	vaccines in general	Single-component interventions	410 minutes	Standard vaccine education
Chase A.J.	2020	NA	vaccines in general	Multiple-component interventions addressed exclusively at HCPs or students	240 minutes	.
Chen G.	2021	2017-2019	Influenza	Multiple-component interventions addressed exclusively at HCPs or students	NA	.
Chen H.	2020	2018-2019	HPV	Multiple-component interventions addressed exclusively at HCPs or students	NA	.
Chidume T.	2020	NA	vaccines in general	Multiple-component interventions addressed exclusively at HCPs or students	1 hour	.
Chin J.	2021	2017-2019	Influenza	Multiple-component interventions addressed not exclusively at HCPs or students	NA	.
Choi N.	2017	2013-2015	HPV	Multiple-component interventions addressed not exclusively at HCPs or students	about 18 months	.
Ciemins E.L.	2020	2014-2016	Influenza and pneumococcal	Multiple-component interventions addressed exclusively at HCPs or students	1 year	No intervention
Cieslowski B.	2020	2017-2019	Influenza	Single-component interventions	6 months	.
Coleman A.	2017	NA	vaccines in general	Multiple-component interventions addressed exclusively at HCPs or students	90 minutes	.
Costello J.	2019	2016-2017	Pneumococcal, MMR, varicella (children with Chronic Kidney Disease, Nephrotic Syndrome, and Renal Transplants)	Multiple-component interventions addressed not exclusively at HCPs or students	approximately 6 months	.

Cotter J.C.	2019	2017	HPV	Single-component interventions	1 hour	.
Dawson R.	2018	2014-2015	HPV	Multiple-component interventions addressed exclusively at HCPs or students	1 hour	.
Dehlinger C.	2021	2018-2020	Influenza	Multiple-component interventions addressed not exclusively at HCPs or students	NA	.
Dempsey A.F.	2018	2014-2016	HPV	Multiple-component interventions addressed not exclusively at HCPs or students	approximately 1 year	Usual care
Deshmukh U.	2018	2014-2015	HPV	Multiple-component interventions addressed not exclusively at HCPs or students	approximately 6 months	.
Drainoni M.L.	2021	2016-2018	HPV	Multiple-component interventions addressed exclusively at HCPs or students	approximately 9 months	.
Dybsand L.L.	2019	2016-2017	vaccines in general	Single-component interventions	16 hours	.
Evans L.	2019	2018-2019	HPV	Multiple-component interventions addressed exclusively at HCPs or students	approximately 90 minutes	.
Farmar A.M.	2016	2013	HPV	Multiple-component interventions addressed not exclusively at HCPs or students	NA	.
Fiks A.G.	2016	2012-2013	HPV	Multiple-component interventions addressed exclusively at HCPs or students	11 months	No intervention
Fiorito T.M.	2021	NA	HPV	Multiple-component interventions addressed exclusively at HCPs or students	140 minutes	.
Fisher-Borne M.	2018	2014-2015	HPV	Multiple-component interventions addressed not exclusively at HCPs or students	2 years	only training without funding
Frederick K.D.	2020	2018-2019	vaccines in general	Single-component interventions	NA	.
Gagneur A.	2019	2016-2017	vaccines in general	Single-component interventions	11 hours	.
Garbutt J.M	2018	2014-2016	HPV	Multiple-component interventions addressed not exclusively at HCPs or students	NA	.
Gatwood J.	2021	2019	Pneumococcal	Multiple-component interventions addressed exclusively at HCPs or students	approximately 6 hours	Intervention 2: online training only (partial training)<br>Control: no intervention
Giduthuri J.G.	2019	2015-2016	Influenza (antenatal)	Multiple-component interventions addressed exclusively at HCPs or students	11 months	No intervention
Giles M. L.	2021	2018-2019	Influenza and pertussis	Multiple-component interventions addressed not exclusively at HCPs or students	NA	.
Gilkey M.B.	2019	2017	HPV	Multiple-component interventions addressed exclusively at HCPs or students	NA	Wait-list
Gingold J.A.	2016	2013	vaccines in general	Multiple-component interventions addressed not exclusively at HCPs or students	approximately 1 year	.
Glanternik J.R.	2020	2015	vaccines in general	Multiple-component interventions addressed exclusively at HCPs or students	NA	.
Hastings T.J.	2019	NA	Pneumococcal, HZV	Multiple-component interventions addressed exclusively at HCPs or students	approximately 6 months	Only the Immunization Update CE (wait-list)
Heaton P.C.	2022	2019-2020	Influenza, pneumococcal, herpes zoster, and Td/Tdap	Single-component interventions	1 year	Pharmacists in the control group were unable to access the vaccination history and

						prediction information for a given patient via ImmsLink; however, they were able to use state IIS portals to look up patient vaccination histories if they had access and were inclined to do so (Before this study, all Rite Aid pharmacies were using ImmsLink to report immunizations to IIS, but the IIS query and recommendation features were not available to them.)
Irving S.A.	2018	2015-2016	HPV	Multiple-component interventions addressed exclusively at HCPs or students	10 months	Usual care: standing orders for vaccination if eligible, walk-in vaccination, electronic medical record prompts, vaccination reminder birthday letters, and vaccine coverage reports of Healthcare Effectiveness Data and Information Set measures.
Jacobs-Wingo J.L.	2017	2013-2015	HPV	Multiple-component interventions addressed not exclusively at HCPs or students	NA	.
Jina a.	2019	2015-2016	Tdap (antenatal)	Multiple-component interventions addressed not exclusively at HCPs or students	NA	.
Jones K.M.	2016	2012-2015	influenza and Tdap (pregnancy)	Single-component interventions	1 year	.
Kaufman J.	2020	2018	antenatal and childhood vaccines	Multiple-component interventions addressed not exclusively at HCPs or students	NA	.
Kawczak S.	2020	2014-2015	Influenza and pneumococcal (populations at high-risk or aged ≥ 65)	Multiple-component interventions addressed exclusively at HCPs or students	8 months	No intervention
Kepka D.	2021	2019-2021	HPV	Multiple-component interventions addressed not exclusively at HCPs or students	NA	.
Kim R.H.	2018	2014-2017	Influenza	Single-component interventions	"one influenza season"	No intervention

Koski K.	2018	2016	vaccines in general	Multiple-component interventions addressed exclusively at HCPs or students	89 minutes	.
Krishnaswamy S.	2018	2015-2017	Pertussis (pregnancy)	Single-component interventions	NA	Two other groups:<br>- a dedicated immunisation nurse-led immunisation service (hospital A)<br>-provision by primary care (hospital C).
Kumar M.M.	2019	2016	HPV	Multiple-component interventions addressed exclusively at HCPs or students	20 minutes	.
Leila R.A.	2021	2021	COVID-19	Multiple-component interventions addressed not exclusively at HCPs or students	NA	.
Lepiller Q.	2020	2018-2019	vaccines in general	Multiple-component interventions addressed exclusively at HCPs or students	NA	.
Lin C.	2016	2013-2014	Influenza	Multiple-component interventions addressed not exclusively at HCPs or students	from 9 months to 2 years	Wait-list
Lin J.L	2018	2016	Influenza, pneumococcal, herpes zoster, and pertussis	Single-component interventions	2 hours	Train-the-trainer group: the champions were instructed to return to their pharmacies, train the rest of their pharmacy's staff members
Loiacono M.M.	2021	2019-2020	Influenza	Multiple-component interventions addressed exclusively at HCPs or students	approximately 6 months	Only training materials (usual standard)
Malo T.L.	2018	2015	HPV	Multiple-component interventions addressed exclusively at HCPs or students	1 hour	.
Malone K.	2016	2013-2014	Pneumococcal (children after kidney transplant)	Multiple-component interventions addressed not exclusively at HCPs or students	approximately 1 year	.
Marchand-Ciriello L.	2020	2016-2017	HPV	Multiple-component interventions addressed not exclusively at HCPs or students	3 months	.
Marotta C.	2017	NA	vaccines in general	Single-component interventions	NA	.
Maurici M.	2019	2015	vaccines in general	Multiple-component interventions addressed exclusively at HCPs or students	18 hours	.
Mazzoni S.E.	2016	2012-2014	Influenza, Tdap, and HPV	Multiple-component interventions addressed not exclusively at HCPs or students	NA	.
McFadden S.M.	2021	2018	HPV	Single-component interventions	NA	.
McGaffey A.	2019	2015-2016	HPV	Multiple-component interventions addressed not exclusively at HCPs or students	approximately 1 year	.
McLean H.Q.	2017	2015-2016	HPV	Multiple-component interventions addressed not exclusively at HCPs or students	approximately 1 year	No intervention
Mitchell G.	2021	2018-2019	Influenza	Single-component interventions	90 seconds to play	.

Morhardt T.	2016	2013-2014	vaccines in general	Multiple-component interventions addressed exclusively at HCPs or students	approximately 5 hours	.
Nissen M.	2019	2014-2016	HPV	Multiple-component interventions addressed not exclusively at HCPs or students	2 years	.
Nold L.	2020	NA	vaccines in general	Single-component interventions	NA	.
Nowalk M.P.	2017	2013-2015	Influenza, pneumococcal, Tdap	Multiple-component interventions addressed not exclusively at HCPs or students	from 9 months to 2 years	.
O'Donnell M.	2018	NA	HZV	Single-component interventions	1 hour	.
O'Leary S.T.	2019	2011-2014	Influenza, Tdap, and HPV	Multiple-component interventions addressed not exclusively at HCPs or students	approximately 8 months	Usual care
Oliver K.	2020	2018	HPV	Multiple-component interventions addressed exclusively at HCPs or students	9 months	.
Olshefski R.S.	2018	2012-2017	Influenza	Multiple-component interventions addressed not exclusively at HCPs or students	5 years	.
Onello E.	2020	2013-2017	vaccines in general	Multiple-component interventions addressed exclusively at HCPs or students	2 months	.
Orefice R.	2019	2015-2017	vaccines in general	Multiple-component interventions addressed not exclusively at HCPs or students	2 months	.
Pahud B.	2020	2017-2018	vaccines in general	Multiple-component interventions addressed exclusively at HCPs or students	approximately 4 hours	Standard vaccine education
Pampena E.	2019	2016-2018	HPV	Multiple-component interventions addressed exclusively at HCPs or students	2 hours	.
Patel M.S.	2017	2010-2013	Influenza	Single-component interventions	"one influenza season"	No intervention
Percy J.N	2019	2017-2018	Pneumococcal	Multiple-component interventions addressed exclusively at HCPs or students	35 minutes	.
Perkins R.B.	2020	2016-2018	HPV	Multiple-component interventions addressed exclusively at HCPs or students	approximately 9 months	.
Perkins R.B.	2020	2016-2018	HPV	Multiple-component interventions addressed not exclusively at HCPs or students	approximately 9 months	Pre-implementation group
Persell S.D.	2020	2015-2018	Influenza	Single-component interventions	3 years	.
Rand C.M.	2018	2013-2014	HPV	Multiple-component interventions addressed exclusively at HCPs or students	9-12 months	.
Rand C.M.	2018	NA	HPV	Multiple-component interventions addressed exclusively at HCPs or students	9-12 months	.
Rao S.	2020	2016-2018	influenza (pediatric)	Multiple-component interventions addressed exclusively at HCPs or students	7 months	.
Real F.J.	2021	2018	HPV	Single-component interventions	approximately 90 minutes	.
Real F.J.	2017	2015-2016	Influenza	Single-component interventions	approximately 20 minutes	Standard vaccine education
Reno J.E.	2018	2015-2016	HPV	Multiple-component interventions addressed not exclusively at HCPs or students	approximately 1 year	.
Reno J.E.	2018	2015-2016	HPV	Multiple-component interventions addressed exclusively at HCPs or students	approximately 1 year	.
Rosen B.L.	2021	2019	HPV	Multiple-component interventions addressed exclusively at HCPs or students	1 day	.

Salous M.H.	2020	2019	HPV	Multiple-component interventions addressed exclusively at HCPs or students	2 hours	.
Sanderson M.	2017	2013-2015	HPV	Multiple-component interventions addressed not exclusively at HCPs or students	NA	No intervention
Sandokji I.	2021	2018-19	Pneumococcal (children with nephrotic syndrome)	Multiple-component interventions addressed exclusively at HCPs or students	12 months	.
Schnaith A.M.	2018	NA	HPV	Multiple-component interventions addressed exclusively at HCPs or students	NA	.
Serino L.	2020	2019	HPV, HZV, pneumococcal and vaccines for adolescents and adults	Single-component interventions	4 hours	.
Shukla A.	2018	2016-2017	HPV	Multiple-component interventions addressed exclusively at HCPs or students	2 hours	.
Skoy E.	2020	2015-2018	vaccines in general	Multiple-component interventions addressed exclusively at HCPs or students	3 hours	.
Spelman J. F.	2022	2021	COVID-19	Multiple-component interventions addressed not exclusively at HCPs or students	6 months	.
Spina C.I.	2020	2017-2018	Influenza and Tdap vaccine	Multiple-component interventions addressed exclusively at HCPs or students	6 months	.
Srirangan K.	2021	NA	vaccines in general	Single-component interventions	NA	.
Steiner C.R.	2021	2017-2018	HPV	Multiple-component interventions addressed exclusively at HCPs or students	3 months	.
Stetson R.C.	2019	2017-2019	vaccines in general	Multiple-component interventions addressed exclusively at HCPs or students	6 months	.
Suryadevara M.	2019	2015-2016	HPV	Multiple-component interventions addressed not exclusively at HCPs or students	NA	.
Szilagyi P.G.	2021	2018-2019	HPV	Single-component interventions	approximately 1 h and 30 min	Usual care (i.e. no intervention from the research team except for 1 brief communication during the intervention period reminding lead practice clinicians that they were in the control group).
Tchoualeu D.D.	2021	2017-2018	children vaccines	Single-component interventions	5-6 days	.
Torabizadeh C.	2020	2017-2018	HPV	Single-component interventions	approximately 180 minutes	.
Traicoff D.	2021	2017-2018	children vaccines	Single-component interventions	5-6 days	.
Vinci D.M.	2021	2018-2019	HPV	Multiple-component interventions addressed not exclusively at HCPs or students	NA	.
Visalli G.	2021	2019	vaccines in general	Single-component interventions	4 hours	.
Vyas D.	2018	2016	vaccines in general	Multiple-component interventions addressed exclusively at HCPs or students	2 weeks	.

Wallace-Brodeur R.	2020	2016-2019	HPV	Multiple-component interventions addressed exclusively at HCPs or students	9 months	.
Werk L.N.	2019	2015-2016	Influenza	Multiple-component interventions addressed exclusively at HCPs or students	7 months	3 arms: (1) no intervention (Control), (2) computerized clinical decision support system (CCDSS), (3) web-based training (WBT)
Wermers R.	2021	2018	Influenza-HPV-MenB	Multiple-component interventions addressed exclusively at HCPs or students	approximately 6 months	.
Whitaker J.A.	2018	2014-2015	vaccines in general	Multiple-component interventions addressed exclusively at HCPs or students	NA	Only the fact-based immunization curriculum consisted of a PowerPoint™ presentation administered over 60 min.
Wiley R.	2019	2016	HPV	Single-component interventions	1 week	.
Wilkinson T.A.	2019	2015-2016	HPV	Single-component interventions	NA	No intervention
Williams S.E.	2021	2019-2020	vaccines in general	Multiple-component interventions addressed exclusively at HCPs or students	approximately 1 hour	Pre-implementation group
Zaidi S.	2020	2015-2017	vaccines in general	Multiple-component interventions addressed not exclusively at HCPs or students	2 years	.
Zimet G.	2017	2013-2014	meningococcal,Tdap , HPV	Single-component interventions	approximately one year	2 arms:<br>1) Simple reminder prompt: computer-generated messages reminding providers of MenACWY, HPV, and Tdap vaccination eligibility (simple prompt)<br>2) control: care providers on the basis of their existing methods for determining eligibility
Zimmerman R.K.	2017	2013-2015	HPV	Multiple-component interventions addressed not exclusively at HCPs or students	from 9 months to 2 years	Wait-list

Table S5. Characteristics of the studies: funding and conflict of interest

First Author	Year of publication	Funding	Conflict of interest
Abdalla A.	2021	None to report	None to report
Abdulla E.	2020	N.A.	None to report
Amare A.T.	2021	Disclosed	None to report
Arogundade L.	2019	None to report	None to report
Austin J. D.	2020	Disclosed	None to report
Barton S.M.	2022	Disclosed	Disclosed
Bechini A.	2019	None to report	None to report
Berenson A.B.	2020	Disclosed	None to report
Berenson A.B.	2020	Disclosed	None to report
Bishop J.M.	2021	Disclosed	None to report
Blake H.	2022	Disclosed	None to report
Boey L.	2021	None to report	Disclosed
Bonville C.A.	2019	Disclosed	None to report
Bradley C.L.	2021	N.A.	None to report
Bradley-Ewing A.	2021	Disclosed	None to report
Bratic J. S.	2019	N.A.	None to report
Brewer N.T.	2017	Disclosed	Disclosed
Brewer N.T.	2021	Disclosed	None to report
Brodie N.	2018	None to report	None to report
Buenger L.E.	2020	None to report	None to report
Casalino E.	2018	None to report	None to report
Cates J.R.	2020	Disclosed	None to report
Cates J.R.	2018	Disclosed	None to report
Chamberlain A.T.	2019	Disclosed	None to report
Chang C.Y.	2021	Disclosed	None to report
Chase A.J.	2020	N.A.	None to report
Chen G.	2021	N.A.	None to report

Chen H.	2020	Disclosed	None to report
Chidume T.	2020	None to report	N.A.
Chin J.	2021	None to report	None to report
Choi N.	2017	Disclosed	None to report
Ciemins E.L.	2020	Disclosed	None to report
Cieslowski B.	2020	None to report	None to report
Coleman A.	2017	None to report	None to report
Costello J.	2019	N.A.	None to report
Cotter J.C.	2019	Disclosed	None to report
Dawson R.	2018	Disclosed	None to report
Dehlinger C.	2021	N.A.	None to report
Dempsey A.F.	2018	Disclosed	Disclosed
Deshmukh U.	2018	Disclosed	None to report
Drainoni M.L.	2021	Disclosed	None to report
Dybsand L.L.	2019	Disclosed	None to report
Evans L.	2019	None to report	None to report
Farmar A.M.	2016	None to report	None to report
Fiks A.G.	2016	None to report	None to report
Fiorito T.M.	2021	None to report	None to report
Fisher-Borne M.	2018	Disclosed	None to report
Frederick K.D.	2020	None to report	None to report
Gagneur A.	2019	Disclosed	None to report
Garbutt J.M	2018	Disclosed	None to report
Gatwood J.	2021	Disclosed	None to report
Giduthuri J.G.	2019	Disclosed	None to report
Giles M. L.	2021	Disclosed	None to report
Gilkey M.B.	2019	Disclosed	None to report
Gingold J.A.	2016	Disclosed	None to report
Glanternik J.R.	2020	Disclosed	Disclosed
Hastings T.J.	2019	Disclosed	None to report

Heaton P.C.	2022	Disclosed	Disclosed
Irving S.A.	2018	Disclosed	Disclosed
Jacobs-Wingo J.L.	2017	None to report	N.A.
Jina a.	2019	Disclosed	None to report
Jones K.M.	2016	Disclosed	None to report
Kaufman J.	2020	Disclosed	Disclosed
Kawczak S.	2020	Disclosed	None to report
Kepka D.	2021	Disclosed	Disclosed
Kim R.H.	2018	Disclosed	Disclosed
Koski K.	2018	Disclosed	None to report
Krishnaswamy S.	2018	Disclosed	Disclosed
Kumar M.M.	2019	Disclosed	None to report
Leila R.A.	2021	N.A.	None to report
Lepiller Q.	2020	None to report	None to report
Lin C.	2016	Disclosed	Disclosed
Lin J.L	2018	None to report	None to report
Loiacono M.M.	2021	Disclosed	Disclosed
Malo T.L.	2018	Disclosed	Disclosed
Malone K.	2016	Disclosed	N.A.
Marchand-Ciriello L.	2020	N.A.	None to report
Marotta C.	2017	Disclosed	None to report
Maurici M.	2019	N.A.	None to report
Mazzoni S.E.	2016	Disclosed	Disclosed
McFadden S.M.	2021	Disclosed	N.A.
McGaffey A.	2019	Disclosed	Disclosed
McLean H.Q.	2017	Disclosed	None to report
Mitchell G.	2021	None to report	N.A.
Morhardt T.	2016	None to report	None to report
Nissen M.	2019	Disclosed	N.A.
Nold L.	2020	None to report	None to report

Nowalk M.P.	2017	Disclosed	N.A.
O'Donnell M.	2018	None to report	None to report
O'Leary S.T.	2019	Disclosed	None to report
Oliver K.	2020	N.A.	None to report
Olshefski R.S.	2018	N.A.	None to report
Onello E.	2020	None to report	N.A.
Orefice R.	2019	N.A.	N.A.
Pahud B.	2020	Disclosed	Disclosed
Pampena E.	2019	Disclosed	N.A.
Patel M.S.	2017	Disclosed	Disclosed
Percy J.N	2019	None to report	None to report
Perkins R.B.	2020	Disclosed	None to report
Perkins R.B.	2020	None to report	Disclosed
Persell S.D.	2020	Disclosed	Disclosed
Rand C.M.	2018	Disclosed	Disclosed
Rand C.M.	2018	Disclosed	Disclosed
Rao S.	2020	None to report	Disclosed
Real F.J.	2021	Disclosed	None to report
Real F.J.	2017	Disclosed	None to report
Reno J.E.	2018	Disclosed	None to report
Reno J.E.	2018	N.A.	None to report
Rosen B.L.	2021	Disclosed	Disclosed
Salous M.H.	2020	Disclosed	None to report
Sanderson M.	2017	Disclosed	None to report
Sandokji I.	2021	N.A.	None to report
Schnaith A.M.	2018	Disclosed	None to report
Serino L.	2020	None to report	None to report
Shukla A.	2018	N.A.	N.A.
Skoy E.	2020	Disclosed	None to report
Spelman J. F.	2022	N.A.	None to report

Spina C.I.	2020	Disclosed	None to report
Srirangan K.	2021	None to report	None to report
Steiner C.R.	2021	None to report	None to report
Stetson R.C.	2019	None to report	None to report
Suryadevara M.	2019	Disclosed	None to report
Szilagyi P.G.	2021	Disclosed	Disclosed
Tchoualeu D.D.	2021	Disclosed	None to report
Torabizadeh C.	2020	Disclosed	None to report
Traicoff D.	2021	Disclosed	None to report
Vinci D.M.	2021	Disclosed	None to report
Visalli G.	2021	None to report	None to report
Vyas D.	2018	N.A.	N.A.
Wallace-Brodeur R.	2020	Disclosed	None to report
Werk L.N.	2019	Disclosed	None to report
Wermers R.	2021	N.A.	None to report
Whitaker J.A.	2018	Disclosed	Disclosed
Wiley R.	2019	N.A.	N.A.
Wilkinson T.A.	2019	Disclosed	Disclosed
Williams S.E.	2021	Disclosed	Disclosed
Zaidi S.	2020	N.A.	None to report
Zimet G.	2017	Disclosed	Disclosed
Zimmerman R.K.	2017	Disclosed	Disclosed

Table S6. Risk of bias for non-randomized studies: Critical appraisal Checklist for Quasi-Experimental Studies, Joanna Briggs Institute (JBI)

**Author & Date**	**1. Is it clear in the study what is the “cause” and what is the ‘effect’ (i.e. there is NO confusion about which variable comes first)?**	**2. Were the participants included in any comparisons similar?**	**3. Were the participants included in any comparisons receiving similar treatment/care, other than the exposure or intervention of interest?**	**4. Was there a control group?**	**5. Were there multiple measurements of the outcome both pre and post the intervention/exposure?**	**6. Was follow up complete and if not, were differences between groups in terms of their follow up adequately described and analyzed?**	**7. Were the outcomes of participants included in any comparisons measured in the same way?**	**8. Were outcomes measured in a reliable way?**	**9. Was appropriate statistical analysis used?**
Abdalla A. 2021	YES	YES	YES	NO	NO	YES	YES	YES	YES
Abdulla E. 2020	YES	YES	YES	NO	NO	YES	YES	YES	YES
Bechini A. 2019	YES	YES	YES	NO	NO	YES	YES	YES	YES
Berenson A.B. 2020	YES	YES	YES	NO	NO	NO	YES	YES	YES
Berenson A.B. 2020	YES	YES	YES	NO	NO	YES	YES	YES	YES
Bishop J.M. 2021	YES	YES	YES	NO	YES	NO	YES	YES	YES
Blake H. 2022	YES	NO	NO	NO	NO	YES	YES	YES	YES
Boey L. 2021	YES	YES	YES	NO	YES	NO	YES	YES	YES
Bonville C.A. 2019	YES	YES	YES	NO	YES	YES	YES	YES	YES
Bradley C.L. 2021	YES	YES	YES	NO	NO	NO	YES	YES	YES
Bratic J. S. 2019	YES	YES	YES	NO	YES	YES	YES	YES	YES
Brodie N. 2018	YES	YES	YES	NO	YES	NO	YES	YES	YES
Buenger L.E. 2020	YES	YES	YES	NO	YES	YES	YES	YES	YES
Casalino E. 2018	YES	YES	YES	NO	YES	YES	YES	YES	YES
Chen G. 2021	YES	YES	YES	NO	NO	NO	YES	YES	YES
Chen H. 2020	YES	YES	YES	NO	NO	NO	YES	YES	YES
Chin J. 2021	YES	YES	YES	NO	YES	YES	YES	YES	YES
Choi N. 2017	YES	YES	YES	NO	NO	YES	YES	YES	YES
Cieslowski B. 2020	YES	YES	YES	NO	YES	YES	YES	YES	YES
Costello J. 2019	YES	YES	YES	NO	NO	YES	YES	YES	NO

Cotter J.C. 2019	YES	YES	YES	NO	NO	NO	YES	YES	YES
Dawson R. 2018	YES	YES	YES	NO	YES	YES	YES	YES	YES
Dehlinger C. 2021	YES	YES	YES	NO	NO	YES	YES	YES	YES
Deshmukh U. 2018	YES	YES	YES	NO	YES	YES	YES	YES	YES
Evans L. 2019	YES	YES	YES	NO	NO	NO	YES	YES	YES
Fiorito T.M. 2021	YES	YES	YES	NO	NO	YES	YES	YES	YES
Gagneur A. 2019	YES	YES	YES	NO	YES	NO	YES	YES	YES
Giles M. L. 2021	YES	YES	YES	NO	YES	YES	YES	YES	YES
Gingold J.A. 2016	YES	YES	YES	NO	YES	YES	YES	YES	YES
Glanternik J.R. 2020	YES	YES	YES	NO	NO	NO	YES	NO	NO
Jacobs-Wingo J.L. 2017	YES	YES	YES	NO	NO	YES	YES	YES	YES
Jina a. 2019	YES	YES	YES	NO	YES	YES	YES	YES	YES
Jones K.M. 2016	YES	YES	YES	NO	NO	NO	YES	YES	YES
Kepka D. 2021	YES	YES	YES	NO	YES	YES	YES	YES	YES
Kumar M.M. 2019	YES	YES	YES	NO	NO	NO	YES	YES	YES
Leila R.A. 2021	YES	YES	YES	NO	YES	YES	YES	YES	YES
Lepiller Q. 2020	YES	YES	YES	NO	NO	NO	YES	YES	YES
Malone K. 2016	YES	YES	YES	NO	YES	YES	YES	YES	YES
Marchand-Ciriello L. 2020	YES	YES	YES	NO	YES	YES	YES	YES	YES
Marotta C. 2017	YES	YES	YES	NO	NO	NO	YES	YES	YES
Maurici M. 2019	YES	YES	YES	NO	NO	YES	YES	YES	YES
Mazzoni S.E. 2016	YES	YES	YES	NO	YES	YES	YES	YES	YES
McFadden S.M. 2021	YES	YES	YES	NO	NO	YES	YES	YES	YES
McGaffey A. 2019	YES	YES	YES	NO	NO	YES	YES	YES	YES
Mitchell G. 2021	YES	YES	YES	NO	NO	NO	YES	YES	YES
Morhardt T. 2016	YES	YES	YES	NO	NO	NO	YES	YES	NO
Nissen M. 2019	YES	YES	YES	NO	YES	YES	YES	YES	YES
Nowalk M.P. 2017	YES	YES	YES	NO	NO	YES	YES	YES	YES
O'Donnell M. 2018	YES	YES	YES	NO	YES	YES	YES	YES	YES

Oliver K. 2020	YES	YES	YES	NO	YES	YES	YES	YES	YES
Olshefski R.S. 2018	YES	YES	YES	NO	YES	YES	YES	YES	YES
Onello E. 2020	YES	YES	YES	NO	NO	NO	YES	YES	YES
Orefice R. 2019	YES	YES	YES	NO	NO	YES	YES	YES	YES
Pampena E. 2019	YES	YES	YES	NO	NO	YES	YES	YES	YES
Percy J.N 2019	YES	YES	YES	NO	NO	NO	YES	YES	YES
Perkins R.B. 2020	YES	YES	YES	NO	YES	NO	YES	YES	YES
Persell S.D. 2020	YES	YES	YES	NO	YES	YES	YES	YES	YES
Rand C.M. 2018	YES	YES	YES	NO	YES	YES	YES	YES	YES
Rand C.M. 2018	YES	YES	YES	NO	YES	YES	YES	YES	YES
Rao S. 2020	YES	YES	YES	NO	YES	YES	YES	YES	YES
Reno J.E. 2018	YES	YES	YES	NO	NO	YES	YES	YES	YES
Reno J.E. 2018	YES	YES	YES	NO	NO	YES	YES	NO	YES
Salous M.H. 2020	YES	YES	YES	NO	YES	NO	YES	YES	YES
Sandokji I. 2021	YES	YES	YES	NO	YES	YES	YES	YES	YES
Schnaith A.M. 2018	YES	YES	YES	NO	NO	NO	YES	YES	YES
Skoy E. 2020	YES	YES	YES	NO	YES	UNCLEAR	YES	YES	YES
Spina C.I. 2020	YES	YES	YES	NO	NO	YES	YES	YES	YES
Steiner C.R. 2021	YES	YES	YES	NO	YES	YES	YES	YES	YES
Stetson R.C. 2019	YES	YES	YES	NO	YES	NO	YES	YES	YES
Suryadevara M. 2019	YES	YES	YES	NO	YES	YES	YES	YES	YES
Tchoualeu D.D. 2021	YES	YES	YES	NO	NO	NO	YES	YES	YES
Torabizadeh C. 2020	YES	YES	YES	NO	NO	YES	YES	YES	YES
Vinci D.M. 2021	YES	YES	YES	NO	YES	YES	YES	YES	YES
Visalli G. 2021	YES	YES	YES	NO	NO	YES	YES	YES	YES
Vyas D. 2018	YES	YES	YES	NO	NO	YES	YES	YES	YES
Wallace-Brodeur R. 2020	YES	YES	YES	NO	YES	NO	YES	YES	YES
Wermers R. 2021	YES	YES	YES	NO	YES	NO	YES	YES	YES
Wiley R. 2017	YES	YES	YES	NO	NO	NO	YES	YES	YES

Abdalla A. 2021	YES	YES	YES	NO	NO	YES	YES	YES	YES
Abdulla E. 2020	YES	YES	YES	NO	NO	YES	YES	YES	YES
Bechini A. 2019	YES	YES	YES	NO	NO	YES	YES	YES	YES
Berenson A.B. 2020	YES	YES	YES	NO	NO	NO	YES	YES	YES
Berenson A.B. 2020	YES	YES	YES	NO	NO	YES	YES	YES	YES
Bishop J.M. 2021	YES	YES	YES	NO	YES	NO	YES	YES	YES
Blake H. 2022	YES	NO	NO	NO	NO	YES	YES	YES	YES
Boey L. 2021	YES	YES	YES	NO	YES	NO	YES	YES	YES
Bonville C.A. 2019	YES	YES	YES	NO	YES	YES	YES	YES	YES
Bradley C.L. 2021	YES	YES	YES	NO	NO	NO	YES	YES	YES
Bratic J. S. 2019	YES	YES	YES	NO	YES	YES	YES	YES	YES
Brodie N. 2018	YES	YES	YES	NO	YES	NO	YES	YES	YES
Buenger L.E. 2020	YES	YES	YES	NO	YES	YES	YES	YES	YES
Casalino E. 2018	YES	YES	YES	NO	YES	YES	YES	YES	YES
Chen G. 2021	YES	YES	YES	NO	NO	NO	YES	YES	YES
Chen H. 2020	YES	YES	YES	NO	NO	NO	YES	YES	YES

Table S7. Risk of bias for cluster randomized controlled studies: RoB 2 assessment

First Author & Date	D1a	D1b	D2	D3	D4	D5	Overall
Bradley-Ewinga A. 2021	+	+	+	+	+	+	+
Brewer N.T. 2017	+	+	+	+	+	+	+
Dempsey A.F. 2018	+	+	+	+	+	+	+
Fisher-Borne M. 2018	+	+	+	-	+	+	-
Gatwood J. 2021	+	+	+	+	+	+	+
Gilkey M.B. 2019	+	+	+	+	+	+	+
Hastings T.J. 2019	+	+	+	+	+	+	+
Heaton P.C. 2022	+	+	+	+	+	+	+
Lin C. 2016	+	+	+	+	+	+	+
Loiacono M.M. 2021	+	+	+	+	+	+	+
Malo T.L. 2018	+	+	+	+	+	+	+
O'Leary S.T. 2019	+	+	+	+	+	+	+
Pahud B. 2020	+	+	+	+	+	+	+
Perkins R.B. 2020	+	+	+	+	+	+	+
Szilagyi P.G. 2021	+	+	+	+	+	+	+
Werk L.N. 2019	+	+	+	+	+	+	+

Whitaker J.A. 2018	+	+	+	+	+	+	+
Williams S.E. 2021	+	+	+	+	!	+	!
Zimet G. 2017	+	+	+	+	+	+	+
Zimmerman R.K. 2017	+	+	+	+	+	+	+

Legend:

- \+ Low risk
- ! Some concerns
- \- High risk

D1a	Randomisation process
D1b	Timing of identification or recruitment of participants
D2	Deviations from the intended interventions
D3	Missing outcome data
D4	Measurement of the outcome
D5	Selection of the reported result

Table S8. Risk of bias for randomized controlled studies: RoB 2 assessment

First author and date	D1	D2	D3	D4	D5	Overall
Giduthuri J.G. 2019	!	+	+	+	+	!
Wilkinson T.A. 2019	!	+	+	+	+	!

+ Low risk

! Some concerns

\- High risk

D1 Randomisation process

D2 Deviations from the intended interventions

D3 Missing outcome data

D4 Measurement of the outcome

D5 Selection of the reported result

Extended results

Single-component interventions

A total of 41 papers described single-component interventions. Details are shown in Table S9 and S10. Considering the interventions, the works of Patel et al. (Patel et al., 2017) and Kim et al. (Kim et al., 2018) referred to the same intervention, as well as the works of Tchoualeu et al (Tchoualeu et al., 2021) and Traicoff et al. (Traicoff et al., 2021) and the works of Berenson et al. published in 2020 (Berenson et al., 2020) and in 2021 (Berenson et al., 2021). In addition, the paper of Real et al. (Real et al., 2021) described the usability of one component of the two-component intervention evaluated in the work of Bishop and colleagues (Bishop et al., 2021), thus it will be considered in the next section along with Bishop et al. paper (Bishop et al., 2021). Therefore, this section will take into account a total of 37 unique interventions. A total of 27 interventions were targeted at HCPs (including one intervention targeted at both HCPs and students (Torabizadeh et al., 2020)), 10 interventions were targeted exclusively at students.

Interventions targeted at healthcare professionals

The identified intervention involving HCPs were in-person educational sessions (n=9), asynchronous learning modalities (n=5), online game/apps (n=1), interventions on EHRs (n=8), and few others (training-the-trainer workshops and toolkit with information) (n=4).

In-person educational sessions' main topics were about scientific update (Abdulla et al., 2020; O'Donnell et al., 2018; Serino et al., 2020; Torabizadeh et al., 2020), communication strategies (Gagneur et al., 2019), or both (Brewer et al., 2017; Dybsand et al., 2020; Lin et al., 2018; Srirangan and Lavenue, 2021). Concerning communication, some interventions focused on specific methods, such as motivational interviewing (Gagneur et al., 2019), announcement training (Brewer et al., 2017) or both presumptive and conversational approach (Dybsand et al., 2020). These interventions lasted from 1 hour (Brewer et al., 2017) up to 16 hours (Dybsand et al., 2020). Most papers analyzed the impact of these interventions on knowledge, attitudes, and concerning confidence/comfort of HCPs in managing patients, showing significant improvements (Gagneur et al., 2019; Torabizadeh et al., 2020), encouraging results without analysing statistical significance (Dybsand et al., 2020; O'Donnell et al., 2018; Serino et al., 2020), or contrasting results (Abdulla et al., 2020). Vaccination rates were taken into account for two interventions; however, results were conflicting (Brewer et al., 2017) or not further analysed (Lin et al., 2018). Last, Srirangan K. et al (Srirangan and Lavenue, 2021) showed good results in participants' satisfaction.

Asynchronous learning interventions were mostly focused both on scientific updating and communication (Blake et al., 2022; Cates et al., 2020; McFadden et al., 2021; Szilagyi et al., 2021), while the intervention by Chamberlain et al. (Chamberlain et al., 2019) was mainly based on communication. Cates et al. included also gamification elements, e.g. points for logging in or participating in each learning activity (Cates et al., 2020). These interventions lasted from 1 hour (Chamberlain et al., 2019) up to 12 hours (Cates et al., 2020). The most frequently assessed outcome was confidence of the HCPs showing promising results (Blake et al., 2022; Chamberlain et al., 2019; Szilagyi et al., 2021) and significant improvements (McFadden et al., 2021). Similar results were reported for knowledge and attitudes (Blake et al., 2022; Chamberlain et al., 2019; McFadden et al., 2021). Szilagyi and colleagues reported a significant increase in patients' vaccination rates (Szilagyi et al., 2021). When investigated, acceptability was good (Blake et al., 2022; Cates et al., 2020; Chamberlain et al., 2019).

Similarly to the asynchronous learning, Real and colleagues proposed a virtual reality game with scenario simulations for medical residents (Real et al., 2017), which lasted about 20 minutes. The authors measured the refusal of vaccination among participants' patients and showed a significant reduction.

Interventions addressed at HCPs through modifications of the EHR were frequent (n=8) and, overall, included alerts, prompts, and standing orders. These changes in the EHR were long-term interventions, going from 6 months (Cieslowski et al., 2020) to 3 years (Persell et al., 2020). The effect on vaccination rates was mixed, going from significant improvements (Kim et al., 2018; Krishnaswamy et al., 2018; Patel et al., 2017; Persell et al., 2020; Zimet et al., 2018) to non-significant results (Heaton et al., 2022; Wilkinson et al., 2019). Only two studies assessed the satisfaction of participants through qualitative methods and reported positive results (Cieslowski et al., 2020; Frederick et al., 2020).

Last, three interventions were the training of trainers (Amare et al., 2021; Arogundade et al., 2019; Tchoualeu et al., 2021; Traicoff et al., 2021), mainly with positive qualitative results about the experience of participants, and one work assessed the revision of a toolkit with immunization information for obstetrics and gynecology HCPs (Jones et al., 2016), which significantly improved attitudes and perceptions of participants.

Interventions targeted at students

The identified intervention involving exclusively students were in-person educational sessions (n=7), online game/apps (n=2), and scenario simulation (n=1).

In-person lectures included both scientific updating and communication strategies (Bechini et al., 2019; Bradley and Vance, 2021; Cotter et al., 2020; Marotta et al., 2017; Wiley et al., 2019) and, in two cases, only scientific updating (Berenson et al., 2021, 2020; Visalli et al., 2021). The interventions lasted from 45 minutes (Berenson et al., 2021, 2020) to a one-week course (Wiley et al., 2019). Overall, good results were reported in knowledge, attitudes, confidence and acceptability (Bechini et al., 2019; Berenson et al., 2021, 2020; Bradley and Vance, 2021; Cotter et al., 2020; Visalli et al., 2021; Wiley et al., 2019). However, Marotta and colleagues reported mixed results in improvement of knowledge among students (Marotta et al., 2017).

The digital tools were a serious game (Mitchell et al., 2021) and a chatbot-based learning approach (Chang et al., 2022). The intervention by Mitchell et al. was a web application in which participants answered random questions, showing a significant improvement in knowledge and encouraging results in attitudes (Mitchell et al., 2021). Chang et al. reported a significant improvement in knowledge and self-efficacy for their intervention that included chatting and asking questions with a bot (Chang et al., 2022).

Last, Nold and colleagues evaluated a scenario simulation intervention, showing good feedback by students (Nold and Deem, 2020).

Multiple-component interventions addressed exclusively at HCPs or students

A total of 60 papers described multicomponent interventions exclusively addressed to HCPs. Details are reported in Table S11 and S12. Considering the interventions, the works of Pampena et al. (Pampena et al., 2020) and Shukla et al. (Shukla et al., 2019) referred to the same intervention, as well as the works of Rand et al. published in 2018 (Rand et al., 2018a, 2018b). In addition, some papers (Drainoni et al., 2021; Perkins et al., 2020a; Reno et al., 2018a) will be considered in the next section because they referred to secondary analysis of main interventions that included actions not involving HCPs/students. Therefore, this section will

take into account a total of 55 unique interventions. A total of 46 interventions were targeted at HCPs (including two interventions targeted at both HCPs and students (Evans et al., 2020; Fiorito et al., 2021)), 9 papers interventions targeted exclusively at students.

Interventions targeted at healthcare professionals

Overall, the majority of interventions (n=35) included at least one action among interventions on the EHR, assessment of vaccination performance (of the participant or of the service) and feedback, and role play or scenario simulation combined with an educational session (in-person or online, which could be synchronous or asynchronous learning) or educational material.

As for interventions that included changes in the EHR (n=9), 5 were combined with actions based on assessment of vaccination performance and feedback (Gilkey et al., 2019; Rand et al., 2018a, 2018b; Rao et al., 2020; Sandokji et al., 2022; Werk et al., 2019). These actions were accompanied by in-person educational sessions and educational material (Gilkey et al., 2019; Sandokji et al., 2022), synchronous learning (Rand et al., 2018a, 2018b), or asynchronous learning (Rao et al., 2020; Werk et al., 2019). Gilkey et al. also used video vignettes to demonstrate strategies for communication (Gilkey et al., 2019). Overall, these interventions were assessed through an evaluation of the increase in vaccination rates or of the reduction of missed opportunities, showing mixed results (Gilkey et al., 2019; Rand et al., 2018a, 2018b; Werk et al., 2019) except the work by Rao et al (Rao et al., 2020) that reported significant improvements. Sandokji and colleagues (Sandokji et al., 2022) measured the vaccination counselling rate revealing an increase after the intervention.
The remaining four interventions on EHRs (Bratic et al., 2019; Buenger and Webber, 2020; Steiner et al., 2021; Stetson et al., 2019) were combined with in-person educational sessions (Bratic et al., 2019; Buenger and Webber, 2020) or educational materials (Steiner et al., 2021; Stetson et al., 2019). Steiner et al. (Steiner et al., 2021) also included training-of-trainers. Also in this case, improvements in vaccination rates or missed opportunities were not consistent: going from significant changes (Stetson et al., 2019) to non-significant results (Steiner et al., 2021).
Beyond the interventions on EHR, other 12 interventions included assessment of performance and feedback, matched with in-person educational sessions (Bonville et al., 2019; Bradley-Ewing et al., 2022; Giduthuri et al., 2019; Irving et al., 2018; Whitaker et al., 2018), online learning (Fiks et al., 2016; Hastings et al., 2020; Kawczak et al., 2020; Oliver et al., 2021; Wallace-Brodeur et al., 2020), educational material (Loiacono et al., 2021) or incentives (Spina et al., 2020). Wallace-Brodeur et al. specifically focused on motivational interviewing (Wallace-Brodeur et al., 2020). Four evaluations reported significant improvements in vaccination rates/missed opportunities (Bonville et al., 2019; Giduthuri et al., 2019; Spina et al., 2020; Wallace-Brodeur et al., 2020), while in most cases the results collected for this outcome domain were mixed (Fiks et al., 2016; Kawczak et al., 2020; Loiacono et al., 2021; Whitaker et al., 2018) or non-significant (Bradley-Ewing et al., 2022; Oliver et al., 2021).
Interventions involving changes in the EHR and/or assessment and feedback on performance, usually lasted several months, from 3 months (Steiner et al., 2021) to 15 months (Bratic et al., 2019).

Considering role play or scenario simulation (n=14), 11 interventions were combined with in-person educational sessions (Austin et al., 2020; Brewer et al., 2021; H. Chen et al., 2021; Dawson et al., 2018; Evans et al., 2020; Fiorito et al., 2021; Glanternik et al., 2020; Malo et al., 2018; Maurici et al., 2019; Morhardt et al., 2016; Rosen et al., 2022), which generally included communication strategies as main topic. Some interventions focused on specific strategies, such as presumptive/announcement approach (Brewer et al., 2021; Glanternik et al., 2020), conversational approach (Morhardt et al., 2016), or both (Malo et al., 2018). In

addition, Maurici and colleagues worked on HCPs empathy along with communication (Maurici et al., 2019). Few works analysed the impact of these interventions on vaccination rates, showing mixed results (Brewer et al., 2021; Dawson et al., 2018; Glanternik et al., 2020). Overall, the evaluations reported significant improvement in knowledge, attitudes/perceptions, or confidence in counselling (Brewer et al., 2021; H. Chen et al., 2021; Dawson et al., 2018; Evans et al., 2020; Fiorito et al., 2021; Malo et al., 2018) and qualitative analysis or satisfaction surveys reported good feedback (Austin et al., 2020; Morhardt et al., 2016; Rosen et al., 2022). Maurici et al. also reported a significant improvement on HCPs empathy (Maurici et al., 2019). As for the other role play/simulation interventions, they were combined with asynchronous learning (Gatwood et al., 2021; Kumar et al., 2019; Pahud et al., 2020). However, in this case, results in knowledge, attitudes/perceptions, and confidence domains were mainly conflicting or non-significant.
Interventions including role play or scenario simulations lasted from to 20 minutes (Kumar et al., 2019) to 18 hours (Maurici et al., 2019).

Overall, interventions that did not include changes on EHR, assessment and feedback, or role play/simulation, were mainly a combination of in-person educational sessions, synchronous and asynchronous learning and sharing of educational materials and reminders (Abdalla et al., 2020; Barton et al., 2022; Brodie and McPeak, 2018; Ciemins et al., 2020; Pampena et al., 2020; Percy et al., 2020; Salous et al., 2020; Shukla et al., 2019; Skoy et al., 2020; Wermers et al., 2021; Williams et al., 2021), ranging from intervention of 35 minutes (Percy et al., 2020) up to 1-year interventions (Brodie and McPeak, 2018; Williams et al., 2021). Results on vaccination rates were not consistent across these studies, ranging from significant improvements (Brodie and McPeak, 2018; Ciemins et al., 2020; Skoy et al., 2020) to non-significant results (Percy et al., 2020). Similarly, findings on knowledge, attitudes, and comfort in recommending vaccinations were mixed (Abdalla et al., 2020; Barton et al., 2022; Pampena et al., 2020; Percy et al., 2020; Salous et al., 2020; Shukla et al., 2019).

Last, one intervention combined online modules on an asynchronous learning platform with a through a smartphone app based on evidence-based recommendation practices (Bishop et al., 2021; Real et al., 2021), reporting significant improvement in knowledge, attitudes, and self-efficacy.

Interventions targeted at students

Most interventions were based on role play and scenario simulations (n=6) combined with in person educational sessions (Chase et al., 2020; Onello et al., 2020; Schnaith et al., 2018; Vyas et al., 2018) and asynchronous learning (Chidume et al., 2020; Coleman and Lehman, 2017; Vyas et al., 2018), often with both scientific updating and communication as main topics. These interventions lasted from one hour (Chidume et al., 2020) up to a course of 2 weeks (Vyas et al., 2018). Overall, there were positive results concerning knowledge, attitudes, and confidence in interacting with patients (Chidume et al., 2020; Onello et al., 2020; Schnaith et al., 2018; Vyas et al., 2018) and good feedbacks from participants (Chase et al., 2020; Coleman and Lehman, 2017; Schnaith et al., 2018; Vyas et al., 2018).

Last, three interventions did not include simulations. Chen et al. combined in-person educational sessions with learning and practicing muscular injections (G. Chen et al., 2021), reporting significant improvement in knowledge, attitudes, and confidence. Lepiller et al. combined asynchronous learning with a practical exercise in which students prepared and managed a primary prevention intervention (Lepiller et al., 2020), showing significant improvement in attitudes and perceptions. Koski and colleagues (Koski et al., 2018) combined videos showing interactions with patients and a writing exercise during which students were instructed to answer from their role as a physician, receiving good feedbacks from participants.

Multiple-component interventions addressed not exclusively at HCPs or students

A total of 38 papers described interventions composed of multiple actions, with at least one action addressed to HCPs. Details are shown in Table S13 and S14. Considering the interventions, the works of Nowalk et al. (Nowalk et al., 2017), and Zimmerman et al. (Zimmerman et al., 2017), and Lin et al. (Lin et al., 2016) referred to the same intervention, as well as the works of Dempsey et al. (Dempsey et al., 2018) and Reno et al (Reno et al., 2018b). Therefore, this section will take into account a total of 35 unique interventions. All interventions were targeted at HCPs.

Overall, the main components that were not addressed to HCPs were targeted at patients or parents (e.g. education, posters and brochures, reminders and recalls, implementation of more convenient services, communication campaigns) or included changes of the workflow or organizational interventions to make vaccines available. Most interventions had long duration, ranging from 3 months (Marchand-Ciriello et al., 2020) to 5 years (Olshefski et al., 2018).

A total of 6 interventions combined changes on the EHR, assessment of performance and feedback, in-person HCP education and actions addressed to patients or parents (Lin et al., 2016; Marchand-Ciriello et al., 2020; Mazzoni et al., 2016; Nissen et al., 2019; Nowalk et al., 2017; Olshefski et al., 2018; Vinci et al., 2022; Zimmerman et al., 2017). Mazzoni and colleagues also used vaccination champions to give direct provider feedback (Mazzoni et al., 2016). Similarly, 2 interventions matched these actions addressed to HCPs with changes in the workflow or organizational intervention to make vaccines available (Farmar et al., 2016; McGaffey et al., 2019). The intervention by McGaffey et al. specifically focused on using a presumptive approach as communication strategies (McGaffey et al., 2019). The impact of these multicomponent interventions on vaccination rates was mixed, going from significant improvements (Mazzoni et al., 2016) to non-significant changes (Marchand-Ciriello et al., 2020).

A total of 5 interventions were mainly composed of changes on the EHR and in-person HCP education, combined with patients/parents actions (Dehlinger et al., 2021; Deshmukh et al., 2018; Jina et al., 2019; Orefice and Quinlivan, 2021) or organizational interventions on vaccines availability (Jina et al., 2019; O’Leary et al., 2019). Deshmukh et al. and Orefice et al. also included the presence of vaccination champions (Deshmukh et al., 2018; Orefice and Quinlivan, 2021). All interventions reported a significant increase in vaccination rates (Dehlinger et al., 2021; Deshmukh et al., 2018; Jina et al., 2019; Orefice and Quinlivan, 2021) except for the intervention that did not include a component addressed to patients (O’Leary et al., 2019).

A total of 8 interventions included assessment and feedback actions along with in-person HCP education (Boey et al., 2021; Cates et al., 2018; Drainoni et al., 2021; Jacobs-Wingo et al., 2017; McLean et al., 2017; Perkins et al., 2020a, 2020b), educational material (Malone et al., 2016) or asynchronous learning (Gingold et al., 2016), all combined with actions for patients/parents. Specifically, one intervention focused on training in motivational interviewing as communication strategies (Drainoni et al., 2021; Perkins et al., 2020a, 2020b). Moreover, Choi et al. included financial incentives (Choi et al., 2018), Cates et al. sharing of best practices and discussions among participants (Cates et al., 2018), and Gingold et al. quality improvement training (Gingold et al., 2016). Overall, vaccination rates were reported as improved. One intervention was also assessed for changes in attitudes and comfort in counselling (Drainoni et al., 2021; Perkins et al., 2020a, 2020b), finding significant improvement after the intervention.

Overall, the remaining interventions were a combination of educational sessions (in-person or online) or educational materials with actions addressed to patients/parents (Abou Leila et al., 2021; Chin et al., 2021; Dempsey et al., 2018; Fisher-Borne et al., 2018; Garbutt et al., 2018; Giles et al., 2021; Kaufman et al., 2020; Kepka et al., 2021; Reno et al., 2018a, 2018b; Sanderson et al., 2017; Spelman et al., 2022; Suryadevara et al., 2019; Zaidi et al., 2020), changes in the workflow (Chin et al., 2021) or organizational interventions to increase vaccines availability (Casalino et al., 2018; Costello, 2019). As for communication strategies, one intervention specifically focused on motivational interviewing (Dempsey et al., 2018; Reno et al., 2018a, 2018b). Moreover, three interventions also considered the presence of vaccination champions (Kaufman et al., 2020; Spelman et al., 2022; Suryadevara et al., 2019). Overall, the results on vaccination rates were positive, except in two cases (Fisher-Borne et al., 2018; Sanderson et al., 2017). Some interventions were not evaluated through the measurement of vaccination rates. Leila et al. measured the percentage of physicians' vaccine advice and of hesitancy with promising results (Abou Leila et al., 2021). Garbutt et al. (Garbutt et al., 2018) and Kaufman et al. (Kaufman et al., 2020) found good feedback for the implementation of their interventions.

Last, Zaidi and colleagues (Zaidi et al., 2020) combined an app that supported HCPs in identification of children who missed vaccinations and organizing the scheduling with actions addressed to parents, finding encouraging qualitative results.

Table S9. Single component interventions: main characteristics of the interventions

Author & Year	In-person educational session		Asynchronous learning		Online game, apps	Interventions on EHR: alerts, prompts, standing orders	Role play or scenario simulation	Training-of-trainers	Educational material	Main results of the evaluation
	SU	CS	SU	CS						
**Interventions targeted at healthcare professionals**										
Srirangan K. 2021°	X	X	.	.	.	.	.	.	.	+
Serino L. 2020***	X	.	.	.	.	.	.	.	.	+
Abdulla E. 2020*	X	.	.	.	.	.	.	.	.	+ -
Torabizadeh C. 2020*	X	.	.	.	.	.	.	.	.	+
Gagneur A. 2019*	.	X	.	.	.	.	.	.	.	+
Lin J.L 2018**	X	X	.	.	.	.	.	.	.	+
Brewer N.T. 2017****	X	X	.	.	.	.	.	.	.	+ -
Dybsand L.L. 2019°	X	X	.	.	.	.	.	.	.	+
O'Donnell M. 2018*	X	.	.	.	.	.	.	.	.	+
Blake H. 2022°	.	.	X	X	.	.	.	.	.	+
Szilagyi P.G. 2021****	.	.	X	X	.	.	.	.	.	+ -
McFadden S.M. 2021*	.	.	X	X	.	.	.	.	.	+
Cates J.R. 2020***	.	.	X	X	.	.	.	.	.	+
Chamberlain A.T. 2019***	.	.	.	X	.	.	.	.	.	+
Real F.J. 2017**	.	.	.	.	X	.	.	.	.	+
Heaton P.C. 2022****	.	.	.	.	.	X	.	.	.	+
Frederick K.D. 2020°	.	.	.	.	.	X	.	.	.	+

Cieslowski B. 2020°	.	.	.	.	.	X	.	.	.	+ -
Persell S.D. 2020*	.	.	.	.	.	X	.	.	.	+
Wilkinson T.A. 2019****	.	.	.	.	.	X	.	.	.	-
Kim R.H. 2018**; Patel M.S. 2017**	.	.	.	.	.	X	.	.	.	+
Krishnaswamy S. 2018**	.	.	.	.	.	X	.	.	.	+
Zimet G. 2017****	.	.	.	.	.	X	.	.	.	+ -
Amare A.T. 2021°	.	.	.	.	.	.	.	X	.	+
Tchoualeu D.D. 2021*; Traicoff D. 2021***	.	.	.	.	.	.	.	X	.	+
Arogundade L. 2019°	.	.	.	.	.	.	.	X	.	+
Jones K.M. 2016*	.	.	.	.	.	.	.	.	X	+
**Interventions targeted at students**										
Visalli G. 2021*	X	.	.	.	.	.	.	.	.	+
Bradley C.L. 2021*	X	X	.	.	.	.	.	.	.	+
Berenson A.B. 2021*; Berenson A.B. 2020*	X	.	.	.	.	.	.	.	.	+
Bechini A. 2019*	X	X	.	.	.	.	.	.	.	+
Cotter J.C. 2019*	X	X	.	.	.	.	.	.	.	+
Marotta C. 2017*	X	X	.	.	.	.	.	.	.	+ -
Wiley R. 2017*	X	X	.	.	.	.	.	.	.	+
Mitchell G. 2021*	.	.	.	.	X	.	.	.	.	+
Chang C.Y. 2021°	.	.	.	.	X	.	.	.	.	+
Nold L. 2020°	.	.	.	.	.	.	X	.	.	+

Abbreviations: CS; Communication strategies; EHR electronic health record; SU Scientific Updating. Notes: Torabizadeh C. 2020: targeted at both healthcare professionals and students
*uncontrolled pre-post study; **non-randomized controlled trial; ***only post intervention evaluation; **** (cluster) randomized controlled trial; °qualitative or mixed method design.
+ : encouraging results in favour of the intervention
+ - : conflicting results
- : non-significant results

Table S10. Single component interventions: main results of the intervention evaluation

Author & Year	Vaccination rate	Knowledge	Attitudes and perceptions	Confidence/comfort and self-efficacy	Acceptability/Reaction of users	Qualitative results (any outcome)
**Interventions targeted at healthcare professionals**						
Srirangan K. 2021°	.	.	.	.	+	+
Serino L. 2020***	.	+	+	.	.	.
Abdulla E. 2020*	.	+ -	.	.	.	.
Torabizadeh C. 2020*	.	+ +	+ +	.	.	.
Gagneur A. 2019*	.	+ +	+ +	+ +	.	.
Lin J.L 2018**	+	.	.	+	.	.
Brewer N.T. 2017****	+ -	.	.	.	.	.

Dybsand L.L. 2019°	.	.	.	+	.	+
O'Donnell M. 2018*	.	+	.	.	.	.
Blake H. 2022°	.	+	.	+	+	+
Szilagyi P.G. 2021****	++	.	.	+	.	.
McFadden S.M. 2021*	.	+ +	.	+ +	.	.
Cates J.R. 2020***	.	.	.	.	+	.
Chamberlain A.T. 2019***	.	.	+	+	+	.
Real F.J. 2017**	.	.	.	.	.	.
Heaton P.C. 2022****	- -	.	.	.	.	.
Frederick K.D. 2020°	.	.	.	.	.	+
Cieslowski B. 2020°	.	.	.	.	.	+
Persell S.D. 2020*	+ +	.	.	.	.	.
Wilkinson T.A. 2019****	- -	.	.	.	.	.
Kim R.H. 2018**; Patel M.S. 2017**	+ +	.	.	.	.	.
Krishnaswamy S. 2018**	+ +	.	.	.	.	.
Zimet G. 2017****	+ -	.	.	.	.	.
Amare A.T. 2021°	.	.	.	.	.	+
Tchoualeu D.D. 2021*; Traicoff D. 2021***	.	+	.	.	+	+
Arogundade L. 2019°	.	.	.	.	.	+
Jones K.M. 2016*	.	.	+ +	.	.	.
**Interventions targeted at students**						
Visalli G. 2021*	.	+ +	.	.	.	.
Bradley C.L. 2021*	.	.	.	+	+	.
Berenson A.B. 2021*; Berenson A.B. 2020*	.	+	+	+ +	.	.
Bechini A. 2019*	.	++	.	.	.	.
Cotter J.C. 2019*	.	+ +	+ +	+ +	.	.
Marotta C. 2017*	.	+ -	.	.	.	.
Wiley R. 2017*	.	+ +	+	.	+ +	.
Mitchell G. 2021*	.	+ +	+	.	.	.
Chang C.Y. 2021°	.	+ +	.	+ +	.	+
Nold L. 2020°	.	.	.	.	.	+

*uncontrolled pre-post study; **non-randomized controlled trial; ***only post intervention evaluation; **** (cluster) randomized controlled trial; °qualitative or mixed method design.
++ all outcomes of this domain had significant results.
+ no analysis on statistical significance in this domain, but encouraging results in favor of the intervention.
+ - some outcomes of this domain had significant results, other outcomes had non-significant results.
- - all outcomes of this domain had non-significant results.

Table S11. Multiple-component interventions addressed exclusively at HCPs or students: main characteristics of the interventions

Author and Year	Interventions on EHR: alerts, prompts, standing orders	Assessment and feedback	Role play or scenario simulation	In-person educational session		Synchronous online learning		Asynchronous learning		Educational materials and reminders	Online game, apps	Arts based tools	Training-of-trainers	Other	Main results of the evaluation
				SU	CS	SU	CS	SU	CS						
**Interventions targeted at healthcare professionals**															
Gilkey M.B. 2019****	X	X	.	X	X	.	.	.	.	X	.	X	.	.	- +
Sandokji I. 2021*	X	X	.	X	.	.	.	.	.	X	.	.	.	.	+
Rand C.M. 2018a*; Rand C.M. 2018b*	X	X	.	.	.	.	X	.	.	.	.	.	.	Telephone calls; practice teams	- +
Rao S. 2020*	X	X	.	.	.	.	.	X	.	.	.	.	.	.	+
Werk L.N. 2019****	X	X	.	.	.	.	.	X	.	.	.	.	.	.	- +
Bratic J. S. 2019*	X	.	.	X	.	.	.	.	.	.	.	.	.	.	- +
Buenger L.E. 2020*	X	.	.	X	.	.	.	.	.	.	.	.	.	.	+
Stetson R.C. 2019*	X	.	.	.	.	.	.	.	.	X	.	.	.	.	+
Steiner C.R. 2021*	X	.	.	.	.	.	.	.	.	X	.	.	X	.	-
Giduthuri J.G. 2019****	.	X	.	X	.	.	.	.	.	.	.	.	.	.	++
Bonville C.A. 2019*	.	X	.	X	.	.	.	.	.	.	.	.	.	Quality improvement training	+
Irving S.A. 2018**	.	X	.	X	X	.	.	.	.	.	.	.	.	.	-
Whitaker J.A. 2018****	.	X	.	X	X	.	.	.	.	.	.	.	.	.	- +
Bradley-Ewing A. 2021****	.	X	.	.	X	.	.	.	.	.	.	.	.	Behavioral nudges	-
Hastings T.J. 2019****	.	X	.	.	.	X	.	X	.	.	.	.	.	.	- +
Oliver K. 2020*	.	X	.	.	.	X	X	.	.	.	.	.	.	Quality improvement training	- +
Fiks A.G. 2016**	.	X	.	.	.	X	.	.	.	.	.	.	.	.	- +
Wallace-Brodeur R. 2020*	.	X	.	.	.	X	X	.	.	.	.	.	.	Quality improvement training	+
Kawczak S. 2020**	.	X	.	.	.	.	.	X	.	.	.	.	.	.	- +
Loiacono M.M. 2021****	.	X	.	.	.	.	.	.	.	X	.	.	.	.	- +
Spina C.I. 2020*	.	X	.	.	.	.	.	.	.	.	.	.	.	Incentives	+
Malo T.L. 2018****	.	.	X	X	X	.	.	.	.	.	.	.	.	.	+
Dawson R. 2018*	.	.	X	X	X	.	.	.	.	.	.	.	.	.	- +

Rosen B.L. 2021°	.	.	X	X	X	.	.	.	.	.	.	.	.	.	+
Morhardt T. 2016°	.	.	X	X	X	.	.	.	.	.	.	.	.	.	+
Evans L. 2019*	.	.	X	X	X	.	.	.	.	.	.	.	.	.	+
Fiorito T.M. 2021*	.	.	X	X	X	.	.	.	.	.	.	.	.	.	+
Austin J. D. 2020°	.	.	X	X	X	.	.	X	X	X	.	.	.	.	+
Chen H. 2020*	.	.	X	X	.	.	.	.	.	.	.	.	.	.	+
Glanternik J.R. 2020*	.	.	X	.	X	.	.	.	.	.	.	.	.	.	- +
Brewer N.T. 2021**	.	.	X	.	X	.	.	.	.	.	.	.	X	.	- +
Maurici M. 2019*	.	.	X	.	X	.	.	.	.	.	.	.	.	.	+
Kumar M.M. 2019*	.	.	X	.	.	.	.	X	X	.	.	.	.	.	- +
Gatwood J. 2021****	.	.	X	.	.	.	.	X	X	.	.	.	.	.	-
Pahud B. 2020****	.	.	X	.	.	.	.	X	X	.	.	.	.	.	- +
Salous M.H. 2020*	.	.	.	X	X	.	.	.	.	X	.	.	.	.	- +
Shukla A. 2018***; Pampena E.	.	.	.	X	X	.	.	.	.	X	.	.	.	.	+
Wermers R. 2021*	.	.	.	X	X	.	.	.	.	X	.	.	.	.	-+
Brodie N. 2018*	.	.	.	X	X	.	.	.	.	X	.	.	.	.	+
Barton S.M. 2022**	.	.	.	.	X	X	.	.	.	.	.	.	.	.	- +
Abdalla A. 2021*	.	.	.	X	.	.	.	.	.	X	.	.	.	.	- +
Percy J.N 2019°	.	.	.	.	.	X	.	.	.	.	.	.	X	.	-
Skoy E. 2020*	.	.	.	.	.	.	.	X	.	X	.	.	.	.	+
Williams S.E. 2021****	.	.	.	.	.	.	.	X	X	X	.	.	.	Quality improvement training	- +
Ciemins E.L. 2020°	.	.	.	X	.	X	.	.	.	.	.	.	.	Learning collaborative model	+
Bishop J.M. 2021*; Real F.J. 2021°	.	.	.	.	.	.	.	X	.	.	X	.	.	.	+
**Interventions targeted at students**															
Schnaith A.M. 2018*	.	.	X	X	.	.	.	.	.	.	.	X	.	.	+
Onello E. 2020*	.	.	X	X	X	.	.	.	.	.	.	.	.	.	+
Vyas D. 2018*	.	.	X	X	X	.	.	X	.	.	.	.	.	.	+
Chase A.J. 2020°	.	.	X	.	X	.	.	.	.	.	.	.	.	.	+
Coleman A. 2017***	.	.	X	.	.	.	.	X	X	.	.	.	.	Podcast	+
Chidume T. 2020***	.	.	X	.	.	.	.	X	X	.	.	.	.	.	+
Chen G. 2021*	.	.	.	X	.	.	.	.	.	.	.	.	.	Learning and practicing intramuscular injections	+

Lepiller Q. 2020*	.	.	.	.	.	.	.	X	.	.	.	.	.	Preparing and managing a primary prevention intervention	+
Koski K. 2018°	.	.	.	.	.	.	.	.	.	.	.	X	.	Writing exercise	+

Abbreviations: CS; Communication strategies; EHR electronic health record; SU Scientific Updating.
*uncontrolled pre-post study; **non-randomized controlled trial; ***only post intervention evaluation; **** (cluster) randomized controlled trial; °qualitative or mixed method design.
+ : encouraging results in favour of the intervention
+ - : conflicting results
- : non-significant results

Table S12. Multiple-component interventions addressed exclusively at HCPs or students main results of the intervention evaluation

**Author and Year**	**Increasing Vaccination rate/Reducing missing opportunities of vaccination**	**Knowledge**	**Attitudes and perceptions and skills**	**Confidence/comfort and self-efficacy**	**Acceptability/Reaction of users**	**Qualitative results (any outcome)**
**Interventions targeted at healthcare professionals**						
Gilkey M.B. 2019****	- +	.	.	.	.	.
Sandokji I. 2021*	.	.	.	.	.	.
Rand C.M. 2018a*; Rand C.M. 2018b*	- +	.	.	.	.	.
Rao S. 2020*	+ +	.	.	.	.	.
Werk L.N. 2019****	- +	.	.	.	.	.
Bratic J. S. 2019*	- +	.	.	.	.	.
Buenger L.E. 2020*	+	.	.	.	.	.
Stetson R.C. 2019*	+ +	.	.	.	.	.
Steiner C.R. 2021*	- -	+	.	.	.	.
Giduthuri J.G. 2019****	++	.	.	.	.	.
Bonville C.A. 2019*	+ +	.	.	.	.	.
Irving S.A. 2018**	- -	.	.	.	.	.
Whitaker J.A. 2018****	- +	- +	+ +	.	.	.
Bradley-Ewing A. 2021****	- -	.	.	.	.	.

Hastings T.J. 2019****	.	.	+ +	- -	.	.
Oliver K. 2020*	-	+	.	+	.	.
Fiks A.G. 2016**	- +	.	.	.	+	.
Wallace-Brodeur R. 2020*	+ +	.	.	.	.	.
Kawczak S. 2020**	- +	.	.	.	.	.
Loiacono M.M. 2021****	- +	.	.	.	.	.
Spina C.I. 2020*	+ +	.	.	.	.	.
Malo T.L. 2018****	.	.	+ +	.	.	.
Dawson R. 2018*	- +	+ +	.	+ +	.	.
Rosen B.L. 2021°	.	.	.	.	.	+
Morhardt T. 2016°	.	.	.	.	.	+
Evans L. 2019*	.	+ +	.	.	.	.
Fiorito T.M. 2021*	.	+ +	.	+ +	.	.
Austin J. D. 2020°	.	.	.	.	+	+
Chen H. 2020*	.	+ +	+ +	.	.	.
Glanternik J.R. 2020*	- +	.	.	+	.	.
Brewer N.T. 2021**	- +	.	+ +	+ +	+	.
Maurici M. 2019*	.	.	.	.	.	.
Kumar M.M. 2019*	.	- +	- +	+ +	.	.
Gatwood J. 2021****	- -	.	.	- -	.	.
Pahud B. 2020****	.	- +	- -	+ +	.	.
Salous M.H. 2020*	.	- +	.	+ +	.	.
Shukla A. 2018***; Pampena E. 2019*	.	+ +	.	+ +	.	.
Wermers R. 2021*	- +	+ +	.	.	.	.
Brodie N. 2018*	+ +	.	.	.	.	.
Barton S.M. 2022**	.	.	+ +	- -	.	.
Abdalla A. 2021*	.	+ +	- +	.	.	.
Percy J.N 2019°	- -	.	- -	.	.	+
Skoy E. 2020*	+ +	.	.	.	.	.
Williams S.E. 2021****	- +	.	.	.	.	.

Ciemins E.L. 2020°	+ +	.	.	.	.	+
Bishop J.M. 2021*; Real F.J. 2021°	.	+ +	+ +	+ +	.	+
**Interventions targeted at students**						
Schnaith A.M. 2018*	.	.	+ +	+ +	+	.
Onello E. 2020*	.	.	+ +	+	.	.
Vyas D. 2018*	.	+ +	.	.	+	.
Chase A.J. 2020°	.	.	.	.	.	+
Coleman A. 2017***	.	.	.	+	+	.
Chidume T. 2020***	.	.	.	+	.	.
Chen G. 2021*	.	+ +	+ +	+ +	.	.
Lepiller Q. 2020*	.	.	+ +	.	.	.
Koski K. 2018°	.	.	.	.	.	+

*uncontrolled pre-post study; **non-randomized controlled trial; ***only post intervention evaluation; **** (cluster) randomized controlled trial; °qualitative or mixed method design.
++ all outcomes of this domain had significant results.
+ no analysis on statistical significance in this domain, but encouraging results in favor of the intervention.
+ - some outcomes of this domain had significant results, other outcomes had non-significant results.
- - all outcomes of this domain had non-significant results.

Table S13. Multiple-component interventions addressed not exclusively at HCPs: main characteristics of the interventions

Author and date	Interventions on EHR: alerts, prompts, standing orders	Assessment and feedback	In-person educational session		Synchronous online learning		Asynchronous learning		Educational material and reminders	Online game, apps	Other	Target of other components	Main results of the evaluation
			**SU**	**CS**	**SU**	**CS**	**SU**	**CS**					
Nissen M. 2019*	X	X	X	.	.	.	.	.	.	.	.	Patients/parents	+
Marchand-Ciriello L. 2020*	X	X	X	X	.	.	.	.	.	.	.	Patients/parents	-
Olshefski R.S. 2018*	X	X	X	X	.	.	.	.	X	.	.	Patients/parents	+
Vinci D.M. 2021*	X	X	X	.	.	.	.	.	.	.	.	Patients/parents	- +
Mazzoni S.E. 2016*	X	X	X	.	.	.	.	.	.	.	Vaccination champions	Patients/parents	+
Lin C. 2016****;Nowalk M.P. 2017*; Zimmerman R.K. 2017****	X	X	.	X	.	.	.	.	.	.	.	Patients/parents	- +
McGaffey A. 2019*	X	X	X	X	.	.	.	.	X	.	.	Patients/parents Change of the workflow	- +

Farmar A.M. 2016***	X	X	X	X	.	.	.	.	.	.	.	Change of the workflow Organizational intervention on the availability of vaccines	+
Deshmukh U. 2018*	X	.	X	.	.	.	.	.	.	.	Vaccination champions	Patients/parents	+
Orefice R. 2019*	X	.	X	.	.	.	.	.	.	.	Vaccination champions	Patients/parents	+
Dehlinger C. 2021*	X	.	X	X	.	.	.	.	X	.	.	Patients/parents	+
Jina a. 2019*	X	.	X	.	.	.	.	.	X	.	.	Parents/patients Organizational intervention on the availability of vaccines	+
O'Leary S.T. 2019****	X	.	X	.	.	.	.	.	.	.	.	Organizational intervention on the availability of vaccines	-
Perkins R.B. 2020a****;Perkins R.B. 2020b°; Drainoni M.L. 2021°	.	X	X	X	.	.	.	.	.	.	.	Patients/parents	+
McLean H.Q. 2017**	.	X	X	X	.	.	.	.	.	.	.	Patients/parents	-+
Boey L. 2021*	.	X	X	.	.	.	.	.	X	.	.	Patients/parents	- +
Choi N. 2017*	.	X	X	.	X	.	.	.	.	.	Incentives	Patients/parents	+
Jacobs-Wingo J.L. 2017*	.	X	X	.	.	.	.	.	.	.	.	Patients/parents	+
Cates J.R. 2018**	.	X	X	.	.	.	.	.	.	.	Sharing practices between participants	Patients/parents	+
Malone K. 2016*	.	X	.	.	.	.	.	.	X	.	.	Patients/parents	+
Gingold J.A. 2016°	.	X	.	.	.	.	X	.	.	.	Quality improvement training	Patients/parents	+
Fisher-Borne M. 2018****	.	.	X	X	.	.	.	.	.	.	.	Patients/parents	- +
Sanderson M. 2017**	.	.	X	.	.	.	.	.	.	.	.	Patients/parents	- -
Spelman J. F. 2022***	.	.	X	.	.	.	.	.	.	.	Vaccination champions	Patients/parents	+
Giles M. L. 2021*	.	.	X	.	.	.	.	.	X	.	.	Patients/parents	+
Suryadevara M. 2019*	.	.	X	X	.	.	.	.	.	.	Vaccination champions	Patients/parents	+
Leila R.A. 2021*	.	.	.	X	.	.	.	.	.	.	.	Patients/parents	+

Dempsey A.F. 2018****;Reno J.E. 2018*; Reno J.E. 2018*	.	.	.	X	.	.	.	X	.	.	.	Patients/parents	+
Chin J. 2021*	.	.	X	.	.	.	.	.	.	.	.	Patients/parents Change of the workflow	+
Casalino E. 2018*	.	.	X	X	.	.	.	.	X	.	.	Organizational intervention on the availability of vaccines	+
Kepka D. 2021*	.	.	.	.	X	X	.	.	X	.	.	Patients/parents	+
Costello J. 2019*	.	.	.	.	.	.	.	.	X	.	.	Organizational intervention on the availability of vaccines	+
Garbutt J.M 2018°	.	.	.	.	.	.	.	.	X	.	.	Patients/parents	+
Kaufman J. 2020***	.	.	.	.	.	.	.	X	X	.	Vaccination champions	Patients/parents	+
Zaidi S. 2020°	.	.	.	.	.	.	.	.	.	X	.	Patients/parents	+

Abbreviations: CS; Communication strategies; EHR electronic health record; SU Scientific Updating.
No intervention was addressed to students.
*uncontrolled pre-post study; **non-randomized controlled trial; ***only post intervention evaluation; **** (cluster) randomized controlled trial; °qualitative or mixed method design.
+ : encouraging results in favour of the intervention
+ - : conflicting results
- : non-significant results

Table S14. Multiple-component interventions addressed not exclusively at HCPs: main results of the intervention evaluation

Author and date	Increasing Vaccination rate/Reducing missing opportunities of vaccination	Attitudes and perceptions and skills	Confidence/comfort and self-efficacy	Acceptability/Reaction of users	Qualitative results (any outcome)
Nissen M. 2019*	+	.	.	.	.
Marchand-Ciriello L. 2020*	- -	.	.	+	.
Olshefski R.S. 2018*	+	.	.	.	.
Vinci D.M. 2021*	- +	.	.	.	.
Mazzoni S.E. 2016*	++	.	.	.	.
Lin C. 2016****;Nowalk M.P. 2017*; Zimmerman R.K. 2017****	- +	.	.	.	.
McGaffey A. 2019*	- +	.	.	.	.
Farmar A.M. 2016***	+	.	.	.	.
Deshmukh U. 2018*	++	.	.	.	.
Orefice R. 2019*	++	.	.	.	.
Dehlinger C. 2021*	++	.	.	.	.
Jina a. 2019*	++	.	.	.	.

O'Leary S.T. 2019****	- -	.	.	.	.
Perkins R.B. 2020a****;Perkins R.B. 2020b°; Drainoni M.L. 2021°	++	++	++	.	+
McLean H.Q. 2017**	-+	.	.	.	.
Boey L. 2021*	++	- +	.	.	.
Choi N. 2017*	++	.	.	.	.
Jacobs-Wingo J.L. 2017*	+	.	.	.	.
Cates J.R. 2018**	++	.	.	.	.
Malone K. 2016*	+	.	.	.	.
Gingold J.A. 2016°	+	.	.	.	+
Fisher-Borne M. 2018****	- +	.	.	.	.
Sanderson M. 2017**	- -	.	.	.	.
Spelman J. F. 2022***	++	.	.	.	.
Giles M. L. 2021*	+	.	.	.	.
Suryadevara M. 2019*	+	.	.	.	.
Leila R.A. 2021*	.	.	.	.	.
Dempsey A.F. 2018****;Reno J.E. 2018*; Reno J.E. 2018*	++	.	.	+	.
Chin J. 2021*	+	.	.	.	.
Casalino E. 2018*	++	.	.	.	.
Kepka D. 2021*	++	.	.	.	.
Costello J. 2019*	+	.	.	.	.
Garbutt J.M 2018°	.	.	.	.	+
Kaufman J. 2020***	.	.	.	+	.
Zaidi S. 2020°	.	.	.	.	+

*uncontrolled pre-post study; **non-randomized controlled trial; ***only post intervention evaluation; **** (cluster) randomized controlled trial; °qualitative or mixed method design.
++ all outcomes of this domain had significant results.
+ no analysis on statistical significance in this domain, but encouraging results in favor of the intervention.
-+ some outcomes of this domain had significant results, other outcomes had non-significant results.
- - all outcomes of this domain had non-significant results.

Details on the main results of the intervention evaluation

Table S15. Single component interventions: details on the main results of the intervention evaluation:

Author & Year	Vaccination rate	Knowledge	Attitudes and perceptions	Confidence/comfort and self-efficacy	Acceptability/Reaction of users	Qualitative results (any outcome)
**Interventions targeted at healthcare professionals**						
Srirangan K. 2021°	.	.	.	.	Overall mean satisfaction: 4.69/5<br>Mean perceived professional development: 4.63/5<br>Mean perception that learning objectives were clear, relevant, and complete: 4.57/5<br>Mean perception that clinical cases and scenarios were applicable, relevant, and diverse: 4.70/5	Pharmacists generally felt well-prepared.
Serino L. 2020***	.	Knowledge questions post-intervention: correct answers ranged from 15.8% to 93%	Attitude questions post-intervention: 96.5% did actively recommend vaccines; 34.2% had difficulty in proposing HPV vaccine to sceptical parents	.	.	.
Abdulla E. 2020*	.	Knowledge questionnaire: a significant difference ($p \leq 0.05$) in knowledge shown for only part of the questionnaire.	.	.	.	.
Torabizadeh C. 2020*	.	The mean total score of the participants' knowledge before the study was 10.47 ± 4.95, immediately after the intervention was 14.64 ± 1.94, and 3 months after the study was 12.78 ± 2.5, showing an increase ($p < 0.001$).	The mean score of perceived behaviour among the participants raised from 39.17 ± 7.30 before, to 40.85 ± 7.33 immediately after the intervention, and decreased slightly to 40.14 ± 6.39 after 3 months. The total increase in the score was significant ($p<0.001$).	.	.	.

Gagneur A. 2019*	.	MI-knowledge acquisition: difference between scores of the day-1 pre-intervention and of the day-2 post-intervention (49.31 ± 16.33 vs 76.39 ± 6.80; p<0.001).	MI-skills application: difference between scores of the day-1 pre-intervention and of the day-2 post-intervention (4.35 ± 3.44 vs 9.70 ± 4.19; p<0.001). MI-skills participant's self-rated use: difference between scores of the day-1 pre-intervention and of the day-2 post-intervention (58.23 ± 23.16 vs 36.88 ± 18.64; p=0.001).	Self-confidence in applying MI in daily vaccination clinic practise: Difference between scores of the day-1 pre-intervention and of the day-2 post-intervention (69.33 ± 15.09 vs 80.80 ± 7.90; p=0.001).	.	.
Lin J.L 2018**	There was a 12.6% and 15.2% average increase in total influenza, pneumococcal, herpes zoster, and pertussis vaccines administered in the whole-staff training group and the train-the-trainer group, respectively.	.	.	The percentage of participants who felt extremely comfortable in recommending vaccinations increased both in the whole-staff training group (from 56% to 67%) and the train-the-trainer group (from 70% to 86%)	.	.
Brewer N.T. 2017****	Clinics receiving announcement training had a rise in HPV vaccine initiation coverage at 6 months for 11- or 12-year-olds (5.4% difference with control, 95%CI 1.1%-9.7%). Clinics that received conversation training did not differ from the control arm among adolescents ages 11 or 12 (p-value> 0.05). Intervention arms did not differ from the control arm considering other ages or other vaccinations, including HPV series completion, Tdap, and meningococcal.	.	.	.	.	.
Dybsand L.L. 2019°	.	.	.	Providers indicated they were more confident (from 6 to 9, on a scale of 1 to 10)	.	Providers noted that MI had a greater impact on

						their relationship with parents.
O’Donnell M. 2018*	.	Difference between pre-intervention scores (mean=3.4, SD=1.2) and post-intervention scores (mean=4.7, SD=1.3) (p<0.001).	.	.	.	.
Blake H. 2022°	.	Knowledge score >=8/10: from pre-intervention 35% to post-intervention 84.6% "Learned something new": 85.8%	.	Confidence score >=8/10: pre-intervention 44.5% to post-intervention 80.2%	Post-intervention: Easy to use: 98.8% Helpful/very helpful: 99.4% No problems with use: 93.8% Good/excellent satisfaction rating: 99.9% Would recommend to others: 98.8%	The intervention has high satisfaction, usability, and relevance to the target audience. Engagement with the intervention increased participants' knowledge and confidence relating to vaccine promotion and facilitated vaccine-promoting behaviours and vaccine uptake.
Szilagyi P.G. 2021****	HPV vaccine initiation was higher by 3.4 percentage points (95% CI, 0.6%-6.2%) in intervention vs control practices for adolescents with at least 1 HPV vaccine–eligible office visit during the intervention period	.	.	After the intervention, most participants reported feeling confident answering questions about the HPV vaccine (85.1%) or talking with hesitant parents (72.3%).	.	.
McFadden S.M. 2021*	.	Scores for all 3 knowledge questions increased significantly (p<0.01) in the post-intervention (correct responses increased between 6 and 12% points per question).	.	HCPs confidence to address common parental HPV vaccine concerns increased: safety: 54% pre-intervention, 92% post-intervention; fertility: 55% pre-intervention, 90% post-intervention; child too young: 68% pre-intervention, 92% post-intervention; and pork gelatine in vaccine manufacturing: 38% pre-intervention, 90% post-intervention (all changes with p<0.001).	.	.
Cates J.R. 2020***	.	.	.	.	96% of participants agreed the course will improve their	.

					practice. 47% said they were either ‘much more likely’ or ‘more likely’ to recommend the vaccine after course participation.	
Chamberlain A.T. 2019***	.	.	Post-intervention: 82% intended to change the way they approach conversations with vaccine-hesitant patients, 87% reported no barriers to preventing changes in how they approach conversations	Post-intervention: 90% said the intervention increased their knowledge of what to say to vaccine hesitant patients, increased their confidence in addressing vaccinations with their pregnant patients, and will help them improve their practice culture regarding maternal vaccine promotion	Post-intervention: 93% indicated the intervention was well organized, 92% felt it met its stated learning objectives	.
Real F.J. 2017**	.	.	.	.	.	.
Heaton P.C. 2022****	When RRs in intervention stores were compared with control stores, no significant differences were found: influenza for patients aged 19–64 years (adjusted RRR 0.99 [95% CI: 0.83–1.17]); influenza for patients aged ≥65 years (adjusted RRR 1.02 [0.86–1.22]); herpes zoster (adjusted RRR 1.07 [0.90–1.28]); and pneumococcal (adjusted RRR 0.95 [0.80–1.14]);). Td/Tdap vaccination rates did not differ between intervention and control stores (adjusted RRR 0.88 [95% CI: 0.73–1.05]).	.	.	.	.	.
Frederick K.D. 2020°	.	.	.	.	.	During the interviews, the pharmacists agreed that the new intervention helped patients live healthier lives by ensuring that the pharmacy kept better track of vaccination needs. The implementation of this intervention helped pharmacy teams

						achieve clinical goals established by the organizational leadership.
Cieslowski B. 2020°	.	.	.	.	.	A focus group confirmed that the redesign improved workflow, but some nurses thought they still triggered too often.
Persell S.D. 2020*	Patients were more likely to receive onsite vaccination in Season 3 compared with either of the previous seasons— adjusted OR for Season 3 versus Season 2 1.14 (95% CI, 1.12–1.16) or adjusted OR for Season 3 versus Season 1 1.07 (95% CI 1.05–1.09).	.	.	.	.	.
Wilkinson T.A. 2019****	No difference in receipt of 2nd or 3rd doses of HPV between the control and intervention groups (aOR 1.52, 95% CI 0.88–2.62).	.	.	.	.	.
Kim R.H. 2018**; Patel M.S. 2017**	There was a significant 9.5–percentage point increase (95% CI, 4.1-14.3; p<0.001) in influenza vaccination rates for the intervention group.	.	.	.	.	.
Krishnaswamy S. 2018**	The most significant change (39% to 91%, p<0.001) was noted at the hospital where standing orders were introduced.	.	.	.	.	.
Zimet G. 2017****	The elaborated prompt arm had a higher rate of HPV vaccination (62%) than the control arm (45%): adjusted odds ratio, 2.76; 95% confidence interval, 1.07 to 7.14. The simple prompt arm did not differ significantly from the control arm with respect to HPV vaccine initiation. MenACWY and	.	.	.	.	.

	Tdap rates did not vary across the 3 arms.					
Amare A.T. 2021°	.	.	.	.	.	All the key informants have reported that the intervention has brought overall change in improving the immunization program of the supervised health facilities.<br>The majority of the key informants mentioned that adopting the new intervention package in the routine immunization program was acceptable.
Tchoualeu D.D. 2021*; Traicoff D. 2021***	.	Reviewing individual questions, the largest average increase was 1.09 points for both the catch-up policy for missed immunization and the simultaneous injection policy. Knowledge of the policy on adverse events following immunization had an average increase of 1.05 points, followed by the decision-making algorithm for when to administer MCV2 or Men A (+0.85), and intervals between doses (+0.80).	.	.	82%of responses related to learning engagement fell into the acceptable range. For the question related to opportunities for practice, 80% fell into the acceptable range	Participants expressed high appreciation and reported knowledge improvement after the training and reported that they changed their immunization data management practices to improve their work.
Arogundade L. 2019°	.	.	.	.	.	Main themes about factors facilitating or limiting the application of reaching every ward: Instructor competence; training effectiveness; experience in applying strategies; training logistics; perception of new training approach.
Jones K.M. 2016*	.	.	Post-intervention survey participants were	.	.	.

			significantly more likely than preintervention survey participants to report that they routinely offer Tdap vaccinations to all patients during pregnancy (76.8% versus 59.3%).			
**Interventions targeted at students**						
Visalli G. 2021*	.	The Knowledge score increased from 2 of the pre-intervention to 19 of the post-intervention questionnaires (p< 0.001, OR: 0.05, 95% CI: 0.01-0.25).	.	.	.	.
Bradley C.L. 2021*	.	.	.	Student confidence increased significantly between the pre-intervention and post-intervention (all 12 individual skills significantly increased).	Students in both cohorts consistently evaluated the in-class live portion of the program positively with the majority of students agreeing with statements about the delivery method on the post-survey.	.
Berenson A.B. 2021*; Berenson A.B. 2020*	.	A high frequency of students improved their knowledge, with a 176% relative change to correct responses for contraindications, 479% relative change to correct responses about HPV testing for sexually active women before vaccination, and 146% relative change to correct responses for whether dosing needs to be restarted after a 6 month gap.	Students experienced a strong increase in improved beliefs and attitudes after the lecture, with an 18% increase in those who agreed that the vaccine provides more benefit than harm	50% change in the frequency of students who felt more comfortable with counselling patients who came into the clinic with other problems; 91% of students felt comfortable with offering the HPV vaccine to 13–17 year olds after the intervention compared with 63% before the intervention<br>Total comfort with counselling increased significantly among medical students by a mean of 1.43 points post-intervention as compared with pre-intervention (p<0.05)	.	.
Bechini A. 2019*	.	Median score of knowledge: from 74.2/100 to 88.8/100; Self-reported	.	.	.	.

		knowledge: from 2/4 to 3/4 Differences between scores before and after the interventions were all statistically significant ($p < 0.001$)				
Cotter J.C. 2019*	.	Comparison of pre-intervention and post-intervention scores showed a significant increase ($p<0.001$) in knowledge, with mean scores increasing from 8.75 on the pre-intervention to 13.32 on the post-intervention	Increase in positive attitudes about providing HPV immunization counselling ($p\leq0.05$).	Increase in confidence and comfort in providing HPV immunization counselling ($p\leq0.05$)	.	.
Marotta C. 2017*	.	Percentage of correct answers significantly improved after the seminar relating to historical, immunological aspects and vaccination strategy ($p<0.001$), and for questions on communication on vaccination between health personnel and general population ($p<0.05$ and $p<0.01$, respectively). Questions on specific immunological and pathogenetic mechanisms showed a not significant decrease of proper responses percentage ($p=0.80$).	.	.	.	.
Wiley R. 2017*	.	Significant improvements from baseline pre-intervention scores (post-test=87.6%, $p<0.001$), general knowledge (post-test=85.3%, $p<0.001$), and vaccine-related knowledge (post-test=91.7%, $p<0.001$).	Post-intervention 100% of participants reported that they would recommend the vaccine compared with 58% in the pre-intervention	.	Post-intervention satisfaction was significantly improved over pre-intervention for general knowledge ($p<0.001$), vaccine education ($p<0.001$), and comfort in patient care ($p<0.001$)	.

Mitchell G. 2021*	.	Nursing students scored an average of 68.6% pre-intervention and 85.2% post-intervention (p<0.001)	Student perception about the importance of promoting the influenza vaccination to their patients and public: 44.0% felt this was very important pre-intervention and this increased to 83.3% post-intervention	.	.	.
Chang C.Y. 2021°	.	About knowledge, the results of the two groups indicated that the intervention (Mean=88.58; SD=11.02) had better results than the control (Mean= 60.51; SD=15.01) (p<0.001).	.	About self-efficacy, the results of the two groups indicated that the intervention (Mean=4.54; SD=0.53) had better results than the control (Mean=3.31; SD=0.45)(p < 0.001)	.	The interview results showed that the intervention group generally believed that the intervention had three advantages, that is, a “user-friendly interface,” “promoting learning engagement,” and “enhancing self-efficacy.”
Nold L. 2020°	.	.	.	.	.	Students reported feeling better prepared for addressing vaccine hesitancy, having greater awareness of their own biases toward vaccine-refusing families, and becoming newly acquainted with their potential role in enforcing practices' dismissal policies for vaccine-refusing families

*uncontrolled pre-post study; **non-randomized controlled trial; ***only post intervention evaluation; **** (cluster) randomized controlled trial; °qualitative or mixed method design.

Table S16. Multiple-component interventions addressed exclusively at HCPs or students: details on the main results of the intervention evaluation:

Author and Year	Increasing Vaccination rate	Knowledge	Attitudes and perceptions and skills	Confidence/comfort and self-efficacy	Acceptability/Reaction of users	Qualitative results (any outcome)
**Interventions targeted at healthcare professionals**						
Gilkey M.B. 2019****	HPV vaccination coverage (≥1 dose) increased by 8.6 percentage points in the intervention group and 6.4 percentage points in the control group (p=0.210).<br>In the subsample of physicians, HPV vaccination coverage increased 10.2 percentage points in the intervention group and 6.9 percentage points in the control group (p=0.030)	.	.	.	.	.
Sandokji I. 2021*	.	.	.	.	.	.
Rand C.M. 2018a*; Rand C.M. 2018b*	In community practices, HPV vaccine initiation rates improved significantly for female adolescents (from 66% to 74%, p<0.01), male adolescents (57% to 65%; p<0.01), and overall (62% to 70%; p<0.01). In continuity clinics, initiation rates increased significantly only for male adolescents (from 68% to 75%; p=0.05) and overall (71% to 77%; p<0.01). Completion rates for the HPV vaccination series improved overall for community practices (from 39% to 43%; p=0.04) and for male adolescents in continuity clinics (36% to 44%; p=0.03)	.	.	.	.	.
Rao S. 2020*	In multiple regression models, adjusting for demographic and other hospital factors, the intervention was associated with 1.23 (95% CI 1.11-1.35) times higher odds of appropriate vaccination screening on admission, 2.27 (95% CI 2.01-2.56) times higher odds of a vaccination being ordered, and 1.39 (95% CI 1.27-1.53) times higher odds of a child being vaccinated against influenza at discharge (all p<0.001).<br><br>Secondary outcome: Among those with known vaccination status, the overall	.	.	.	.	.

	influenza vaccination rate at discharge (taking into account vaccination prior to hospital admission as well as vaccine administered during hospitalization) was 61.2% during the pre-intervention period, which increased to 69.9% during the intervention period (p = 0.003)					
Werk L.N. 2019****	There was a significantly higher likelihood of patient encounters in the WBT intervention arm with adherence to vaccination recommendations during the intervention period compared with that in the preintervention period (OR=1.26, p<0.05). The mean OR were 1.04, 1.00, and 0.81 in the CCDSS, BOTH, and Control arms, respectively. In pairwise comparisons, mean log (OR +1) in the WBT arm was statistically different than in the Control arm, p=0.03, and although nonsignificant, trended higher than in the CCDSS (p=0.22) and BOTH (p=0.15) arms. Comparing the intervention arms over time, there was a significantly increasing trend in mean log (OR +1) in the WBT arm (p<0.05), and, even though not significant, there was a trend toward an increase in mean log (OR +1) over time in the CCDSS arm (p=0.27).	.	.	.	.	.
Bratic J. S. 2019*	Influenza vaccine uptake declined from the pre-BPA year (47.2%; 95%CI: 47.0, 47.4) to the last study year (45.1%; 95%CI: 44.9, 45.2). BPA displays were increasingly ignored by clinical staff throughout the study years from 59.6% in 2014–2015 to 72.5% in 2016–2017.<br>Influenza vaccine uptake for paediatric outpatient services overall declined from the pre-BPA year compared with subsequent study years (p<0.001).<br>Within primary care practices, the BPA significantly reduced missed vaccination opportunities in both sick and well-child visits (p<0.001).	.	.	.	.	.
Buenger L.E. 2020*	After implementation of the intervention for the 2014 to 2015 influenza season, the	.	.	.	.	.

	ED administered 1320 doses of influenza vaccine (20 times higher than the vaccination rate the previous year)					
Stetson R.C. 2019*	During the 6-month intervention phase, the percentage of infants up to date on their immunizations increased to 93.5%. The rate was similar during the first 13 months of the after-intervention phase with 92.5% of infants up to date. The combined rate of fully immunized infants during the intervention and after-intervention phases was 92.8% (significantly improved from the baseline p=0.001)	.	.	.	.	.
Steiner C.R. 2021*	For all 11–14 year-old patients, acceptance of the vaccine increased (p=0.183)	All respondents reported comfort with making same way/same day vaccine recommendations, although only 57% indicated this was because of the class.	.	.	.	.
Giduthuri J.G. 2019****	Median vaccination rates increased from 2.6% in SP1 to 12.2% in SP2 (OR=5.2, 95%CI: 2.4–11.0) among intervention group, but rates remained stable among control group (0.2% in SP1 and 0.1% in SP2). Among intervention group, the median rate increased further from SP2 to SP3 (OR=4.4, 95% CI: 2.4–7.9). After the second interaction (SP3), intervention group clinicians were vaccinating at a higher rate of 37.8%, while the rate in control group remained unchanged (0.2%). After the first and second interventions, the differences in rates between intervention and control group were significant (p<0.05).<br>SP: study period (SP1 = before first interaction, SP2 = between 1st and 2nd interaction, SP3 = after 2nd interaction).	.	.	.	.	.
Bonville C.A. 2019*	HPV vaccine completion rates among eligible 11–12-year-old patients increased by 19% after the intervention (p<0.05). HPV vaccine initiation rates increased by 15% (p<0.05).	.	.	.	.	.

Irving S.A. 2018**	The change in trend was significantly greater at control clinics, compared with intervention clinics (p=0.002). No significant differences in HPV vaccine coverage were identified at intervention clinics.	.	.	.	.	.
Whitaker J.A. 2018****	No interaction between the study group type and the pre-study to post-study vaccination rates was observed. The odds ratios were calculated for vaccination, post-study relative to pre-study for the entire study cohort. Odds Ratio (95%) Post-relative to Pre-study immunization:<br>Influenza 4.61 (4.18, 5.08) (p<0.001)<br>Pneumococcal 0.96 (0.81, 1.14) (p=0.650)<br>Tetanus: 1.07 (0.97, 1.17) (p=0.160)<br>Pertussis: 1.17 (1.08, 1.28) (p<0.001)<br>Zoster: .09 (0.98, 1.21) (p=0.060)	There was a significant increase in the scores in both the groups over the study. The control group had an immunization knowledge pre-study score of 32.7% that improved to 40.5% post-study (p=0.01). The intervention group had an immunization knowledge pre-study score of 35.8% that improved to 42.4% post-study (p=0.03). There was no improvement in the cognitive styles' knowledge score over the course of the study in the control group. There was an improvement in the cognitive styles scores for the intervention group pre-study to post-study (p=0.03)	The intervention group had significant improvements in confidence in answering fact-based questions and concerns about immunization side-effects (p=0.02), confidence in addressing patients' fears about immunizations (p=0.002), confidence in increasing patients' self-efficacy in the vaccine decision making process (p=0.01), and confidence in using social norming approaches to address vaccine misperceptions and refusal (p=0.002).	.	.	.
Bradley-Ewinga A. 2021****	Three of the four practices evidenced an increase in HPV vaccination rates and there was a statistically significant difference by practice; however, there was no significant difference by study arm.	.	.	.	.	.
Hastings T.J. 2019****	.	.	Perceived influence on immunization services (p<0.001) significantly improved from baseline to post intervention within the intervention group. No change was observed within the control group. When compared with control group, intervention	Considering confidence, when compared with control group, intervention group showed no changes in all measures (p>0.05)	.	.

			group showed a higher degree of change in perceived influence on immunization services (p=0.005)			
Oliver K. 2020*	Rates of missed opportunities to vaccinate at all visit types fell by 13 percentage points (38%–25%).	Providers reported increased knowledge on HPV vaccine communication with hesitant families.	.	Providers reported increased confidence on HPV vaccine communication with hesitant families.	.	.
Fiks A.G. 2016**	Participants in the intervention group had a significant relative increase in captured opportunities compared with nonparticipants for HPV dose 1 at preventive visits (5.8 percentage points [95% CI 3.8 to 7.7]) and doses 1 and 2 at acute visits (0.7 [0.1 to 1.2] and 5.5 [0.7 to 10.3] percentage points, respectively). Intervention participants had relative increases that did not reach significance for dose 2 at preventive visits and dose 3 at acute visits (3.2 [−2.3 to 7.9] and 5.4 [−0.6 to 11.5], respectively)	.	.	.	Nearly all providers felt each component of the intervention (educational presentation, data summarizing vaccination rates, and team meetings to discuss the project) was important. Participants felt that the project was relevant to patient care, benefited from collaboration and group discussion, found peer comparison to be a motivating factor, and did not find the program burdensome. Most participants reported that they would not change anything about the program. Some found it difficult to attend group calls.	.
Wallace-Brodeur R. 2020*	HPV vaccination rates: Participants had a 13.1 percentage point increase in initiation (p=0.03) and 27 percentage point increase in completion rates (p<0.01).	.	.	.	.	.
Kawczak S. 2020**	The difference between intervention versus control groups was 3.4% higher for influenza ≥ 65 years (p<0.001), 2.1% for influenza high-risk (p<0.001), 0.6% for pneumococcal ≥ 65 years (p<0.001), and	.	.	.	.	.

	1.4% for pneumococcal high-risk (p=0.059)					
Loiacono M.M. 2021****	Across nearly all strata, the mean and median numbers of doses administered among the intervention pharmacies were greater than those of the control pharmacies. Stratifying by historical performance among large- and small-format pharmacies, SIV uptake varied substantially.<br>Overall, the intervention pharmacies administered an average of 3.7% (95% CI, –0.3% to 7.9%) additional doses compared with the control pharmacies	.	.	.	.	.
Spina C.I. 2020*	Baseline documented influenza vaccine receipt was 56% and increased to 65% following the intervention (p<0.01) across both states. Baseline Tdap vaccine receipt among all practices was 77% and increased to 84% following the intervention (p<0.02)	.	.	.	.	.
Malo T.L. 2018****	.	.	Providers had increases in positive attitudes toward HPV vaccination (p<0.001), in subjective norms (p<0.001) and perceived behavioural control to recommend the vaccine (p<0.001)	.	.	.
Dawson R. 2018*	There was no significant difference in the overall number of vaccines given at all clinics after the intervention (p=0.324)	The intervention significant increased knowledge about the HPV vaccine information, such as the side effects, differences in recommendations for male and female patients, and time-interval between each vaccine (p<0.001)	.	A significant increase in provider comfort level in answering patients' and parents' questions about the HPV vaccine was observed (p=0.003)	.	.
Rosen B.L. 2021°	.	.	.	.	.	The major strength identified specific to the activity included opportunity to practice evidence-based recommendation skills (n=10). Weaknesses of the activity identified

						included<br>lack of diversity (n=4) and complexity within the scenarios (n=2)
Morhardt T. 2016°	.	.	.	.	.	Participants found that the curriculum improved self-perceived confidence and competence in speaking to vaccine-hesitant families. Pre- and postintervention simulated encounter evaluations showed resident improvement in seven of eight domains, which were using open-ended questions, listening and eliciting concerns, counselling about risks, and their comfort, knowledge, and ability to explain about vaccines
Evans L. 2019*	.	Medical students improved in knowledge of HPV-associated malignancies (mean score improved from 3.53 to 4.67), symptoms (1.76 to 2.63), transmission (3.05 to 3.87), and vaccination schedule (1.21 to 1.68) (p<0.001)	.	.	.	.
Fiorito T.M. 2021*	.	100% of participants answered the knowledge questions correctly following the intervention. All pre-post differences were statistically significant (p<0.0001)	.	There was an increase in confidence in promoting the HPV vaccine following the activity (from mean score 3.7 pre-intervention to 4.5 post-intervention, p<0.0001)	.	.
Austin J. D. 2020°	.	.	.	.	Providers and staff participating in the surveys and interviews	Providers and staff participating in the surveys and interviews

					requested revisions to the training to improve program delivery by decreasing barriers to participation be creating flexible time options for completing the training and to better tailor the content	requested revisions to the training to improve program delivery by decreasing barriers to participation be creating flexible time options for completing the training and to better tailor the content
Chen H. 2020*	.	Knowledge questionnaire: When questioned about prevention, in the pre-intervention questionnaire, 81.9% thought that HPV vaccination was the most effective way to prevent HPV, compared with 91.9% in the post-questionnaire (p<0.001). 88.3% and 81.6% of the participants believed that HPV vaccine was safe and effective before intervention, and the proportion increased significantly to 97.2% and 93.6% following the educational intervention (p<0.001). When the side effects of HPV vaccination were mentioned, 73.2% responded correctly before intervention compared with 81.2% after the intervention (p<0.001). Only 53.7% knew that "HPV vaccination was not recommended during pregnancy and lactation" pre-intervention compared to 65.4% post-intervention (p<0.001).	Attitudes questionnaire: When questioned about their intention to recommend HPV vaccination to public, a significant increase (71.9% vs. 82.3%, p<0.001) in participants' willingness was observed.	.	.	.

		Less than half (44.3%) of the participants provided the correct answer that “HPV testing was not required before vaccination” before the intervention and 57.7% chose the right answer after the intervention (p<0.001)				
Glanternik J.R. 2020*	In the post-training period, the adjusted odds of missing at least 1 of the visit's recommended vaccines were 1.14, 1.17, and 1.18 at the 2-, 4-, and 6-month visits, respectively. Overall (after further adjusting for age) they increased—from the pretraining period to the post-training period—by 15% (95% CI, 6%-25%). The increase in the odds of missing a vaccine was consistent with the increase in the proportion of infants receiving no vaccines, and the decrease in the proportion of infants receiving all the recommended vaccines by the 6-month visit, although neither of these findings was statistically significant. The average number of days undervaccinated increased from 65.9 days (6.2% of total possible undervaccinated days) in the pretraining period to 75.5 days (7.1% of total possible undervaccinated days) in the post-training period. After adjusting for the age at visit and clustering among patients of the same physician, the adjusted estimate of the increase in days undervaccinated from the pre-to post-training period was 8.8 days (95% CI, −2.2 to 15.2).	.	.	Most participants responded that the intervention helped them feel to much more or more comfortable discussing vaccination with parents who were unsure about (72.3%), want to delay (73.9%), or who refuse (63.5%) vaccinations	.	.
Brewer N.T. 2021**	Overall, initiation of HPV vaccine for patients ages 11 through 12 increased (p<0.001). Across the two time periods, initiation was higher in the intervention than in the control (p<0.001). The increase for the intervention was 1.9 percentage points larger than that of the control.	.	Positive attitudes toward HPV vaccination increased from pre- to post-intervention (mean=4.31, SD=0.94 vs. mean=4.49, SD=0.86, p<0.001)	The training was also associated with increases in recommendation self-efficacy (p<0.001).	Most attendees agreed that the announcement approach would fit into their clinic’s workflow (90%) and well-child visits (92%). Most participants also agreed	.

	For patients ages 13 through 17, the increase in vaccine initiation over time was 1.5 percentage points larger for the intervention than that of the control. The main effect of intervention condition was not statistically significant (p= 0.083).				they would recommend the training (93%) and that the teacher was effective (94%).	
Maurici M. 2019*	.	.	.	.	.	.
Kumar M.M. 2019*	.	There were significant postintervention increases in the proportion of providers correctly answering most of the questions (4 out of 6 questions)	There were significant postintervention increases in the proportion of providers with positive attitudes (4 out of 9 questions)	There were significant postintervention increases in the proportion of providers who were comfortable in addressing concerns by vaccine hesitant parents (8 out of 8 questions)	.	.
Gatwood J. 2021****	Doses of pneumococcal vaccines were lower (-11.3%) across all stores that underwent the intervention, versus changes of -22.0% (p=0.084) and -9.4% (p=0.0199) in control and online-only training comparison, respectively	.	.	Completing the full intervention led to changes in pharmacist self-efficacy across the measured items (p>0.050).	.	.
Pahud B. 2020****	.	Average knowledge scores increased from pre-intervention (control 53%; intervention 53%) to post-intervention (control 58%; intervention 60%). Increases in vaccine knowledge among Family Medicine residents were greater for intervention compared to controls (p=0.041). (not significant for paediatrics and med-peds)	Among residents who completed both the pre- and post-intervention survey, 13% residents were categorized as "vaccine hesitant" in the pre-intervention survey. 61% moved to the "confident" category in the post- survey (there was no difference between intervention and control groups)	From the pre-intervention to post-intervention period:<br>- self-reported vaccine expertise increased in both the control group (from 49% to 56%) and the intervention group (from 46% to 61%). The increase was greater in the intervention group (p<0.001).<br>- self-confidence to discuss vaccines: intervention (score from 53 to 69), control (score from 56 to 63) (p<0.001)<br>- feeling well prepared: intervention (from 55 to 69), control (from 57 to 64) (p<0.001)	.	.
Salous M.H. 2020*	.	Knowledge questionnaire:<br>- HPV infection prevalence: correct in 83.3% pre-intervention, 92.3% post (p<0.01);<br>- HPV is a sexually	.	When asked about comfortability discussing the HPV vaccine with patients: 24.4% pre vs. 29.6% post identified as "very comfortable" and 34.5% vs.	.	.

		transmitted infection: correct in 77.3% pre, 91.8% post (p<0.01);<br>- correctly selected oropharyngeal cancer: 74.8% pre, 94.7% post (p<0.01);<br>-correctly selected cervical cancer: 93.3% pre, 99.1% post (p>0.01); [..]<br>- When asked to "select all that apply" among diseases that are reduced or prevented by the HPV vaccine: 63.2% pre, 91.3% post correctly selected head and neck cancers (p<0.01); 93.2% vs. 97.4% correctly selected cervical cancers (p>0.05); 48.7% vs. 85.2% correctly selected genital warts (p<0.01); 10.3% vs. 11.3% incorrectly selected chlamydia (p>0.05); and 6.8% vs. 7.0% incorrectly selected HIV (p>0.05)<br><br>A secondary assessment of the post-Q and follow-Q seems to show an increased percentage of correct responses for most of the questions and suggests a retained knowledge of HPV over time.		47.8% identified as "A little comfortable" (p<0.01)		
Shukla A. 2018***; Pampena E. 2019*	.	- 91.1% pre thought that it was possible to prevent HPV compared with 97.7% post (p<0.05)<br>- 89.9% pre thought that HPV vaccine could be	.	28.9% pre were very comfortable in talking with patients about HPV prevention vs 44.7% post (p<0.001)	.	.

		administered to boys, vs. 99.2% post (p<0.001)<br>- 65.6% pre thought that HPV vaccine could prevent genital warts, compared with 89.8% post (p < 0.001)<br>- 66.7% pre thought that HPV vaccine could have prevented oro-phar. cancers, vs. 88.3% post (p<0.001)<br>- When asked about the correlation between HPV vaccine and HIV, 88.9% pre thought that there was no correlation, vs. 93.9% post (p<0.05)				
Wermers R. 2021*	The number of influenza vaccines given increased by 9.71% from the fall of 2017 to the fall of 2018. The number of HPV vaccines decreased by 2.84%. Meningitis B vaccines decreased by 67.23%.	There was a significant increase in the scores for the pre-intervention (mean=20.57, SD=2.6) and post-intervention (mean=22.52, SD=2.67) (p=.004)	.	.	.	.
Brodie N. 2018*	There was a significant rise in vaccine initiation rates (56–84%) in patients aged 9–10 years	.	.	.	.	.
Barton S.M. 2022**	.	.	Increase in post-intervention scores (maximum = 30) was detected in the intervention group, but not in control group (median, 21.3 [IQR, 19.8-24.8] vs 18.8 [IQR, 16.9-20.9]; p<0.001)	Self-confidence increased equally in both intervention and control group	.	.
Abdalla A. 2021*	.	The intervention post-intervention was significantly better that the pre-intervention	Significant increase in the score of beliefs related to knowledge from an average of 3.9 before to 4.5 after the intervention with a mean difference of 0.64. The other 3 aspects of beliefs did not show any significant difference	.	.	.

Percy J.N 2019°	8% increase in pneumococcal vaccination rates after intervention, no difference with before the intervention (p>0.05)	.	Non-significant differences in vaccine perceptions before and after the intervention	.	.	Key facilitators: readiness for program implementation; leadership engagement facilitated technician involvement; pharmacist-extender vaccination program implementation must be compatible with current pharmacy workflow processes; implementation strategy should involve a multimodal plan for engaging participants; champions vital to implementation<br>Key barriers: the intervention was overly complex
Skoy E. 2020*	Annual immunizations delivered by pharmacists augmented by nearly 4000 doses. The number of pharmacist-delivered adult immunization doses of PPSV23 increased (p<0.001) after controlling for seasonality	.	.	.	.	.
Williams S.E. 2021****	The prespecified primary outcome was Combination 10 (Combo10) vaccination status (binary: fully immunized versus not fully immunized). The secondary outcome was Combo10 The intervention effect was not significant on the primary outcome (OR = 1.01; 95% CI [0.76, 1.34]; p>0.9), however there were positive intervention effects in secondary and exploratory models analysing Combination10 rates without flu, including models adjusting for variation over time (p= .01) and practice type p=0.03)	.	.	.	.	.
Ciemins E.L. 2020°	Intervention providers demonstrated greater improvement than their matched providers in PV for patients aged 65 and older (treatment effect: 4.3%, p<0.05) and PV for high-risk patients (2.7%, p<0.001)	.	.	.	.	Most frequently reported facilitators included shared learning with other organizations in the collaborative, use of

						care gap reports, collaboration with specialists, standing orders for vaccine administration, EHR-integrated algorithms, patient and provider/staff education, social media and patient portals, seasonal flu clinics, access to Medicare annual wellness visits for vaccination, and an organizational priority around adult vaccinations. Most frequently reported barriers included documentation issues (eg, EHR limitations, poor documentation of high-risk conditions, provider dictation of notes), and patients receiving vaccinations outside the system.
Bishop J.M. 2021*; Real F.J. 2021°	.	Knowledge score: significant increase from T1 (mean 12.9, SD 1.5) to T2 (15.0, SD 0.00) (p=0.002) and from T1 to T3 (14.7, SD 0.6) (p=0.003).	Attitude score: recommendation for female patients: significant increase from T1 (mean 29.5, SD 4.0) to T2 (32.2, SD 3.3) (p=0.005) and from T1 to T3 (32.2, SD 3.3) (p=0.005). recommendation for male patients: significant increase from T1 (mean 21.3, SD 2.6) to T2 (23.2, SD 2.0) (p=0.012) and from T1 to T3 (23.0, SD 1.9) (p=0.006).	Self-efficacy score: significant increase from T1 (mean 40.8, SD 5.8) to T2 (51.5, SD 4.6) (p=0.001) and from T1 to T3 (51.4, SD 5.3) (p=0.001).	.	Residents described the app as interactive, easy to use, succinct, informative, engaging, and practical.
**Interventions targeted at students**						
Schnaith A.M. 2018*	.	.	Students increased their awareness of the benefits of the HPV vaccine by an average of 0.82 points (95% CI 0.66–0.97, p<0.01)	Students increased their comfort level in talking to vaccine hesitant parents by an average of 1.37 points (95% CI 1.20–1.54, p<0.01).	More than 90% of participants found the intervention approach useful also to discuss other medical issues	.

Onello E. 2020*	.	.	Student participants demonstrated a significant shift toward a more pro-vaccine stance following the intervention	Students demonstrated a perceived increase in comfort talking to vaccine hesitant patients (78.9% vs 97.8%)	.	.
Vyas D. 2018*	.	Significant improvement. The largest changes were seen in knowledge about the use of thimerosal as a preservative and knowledge about vaccinations not overwhelming a child’s immune system	.	.	78.5% of the students were “satisfied or very satisfied” with the offerings	.
Chase A.J. 2020°	.	.	.	.	.	Students experienced the assignment as an engagement in creative play which led to innovative insights
Coleman A. 2017***	.	.	.	98% reported an increase in their confidence in discussing vaccines with patients	93% enjoyed learning from the intervention and 89% would recommend this approach	.
Chidume T. 2020***	.	.	.	95.92% of students strongly agreed with feeling more confident in communicating with patients; 93.9% strongly agreed with feeling more confident in teaching patients	.	.
Chen G. 2021*	.	Knowledge items: participants answered on average 2.96 (standard deviation [SD]=1.88) correctly pre-intervention, while they answered 7.96 (SD =2.09) correctly post-intervention (p<0.0004)	Attitude items: participants on average rated a 6.80 (SD=0.40) for the importance of knowledge of fu vaccines and vaccinations for their future practice as a physician in the post-intervention, higher than the average rating of 6.49 (standard deviation [SD]=0.92) in the pre-intervention (p=0.02).	Self-rated proficiency items: participants on average rated their preparedness significantly higher in the post-intervention (p<0.0004)	.	.
Lepiller Q. 2020*	.	.	Misconceptions and hesitancy concerning vaccines were significantly improved after the intervention	.	.	.

Koski K. 2018°	.	.	.	.	.	The exercise simulates a face-to-face patient encounter, inviting the student to imagine the parent is sitting in the consultation room with them. Several students elicited the parents' concerns through counter questions, which is considered a helpful strategy for addressing vaccine hesitancy. However, according to the criteria used in the study, which is the engagement in a humane 'I-You' health care dialogue and the acknowledgement of the parents' concerns in their lifeworld, 3 out of the 9 students failed

*uncontrolled pre-post study; **non-randomized controlled trial; ***only post intervention evaluation; **** (cluster) randomized controlled trial; °qualitative or mixed method design.

Table S17. Multiple-component interventions addressed not exclusively at HCPs: details on the main results of the intervention evaluation:

	Increasing Vaccination rate/Reducing missing opportunities of vaccination	Attitudes and perceptions and skills	Confidence/comfort and self-efficacy	Acceptability/Reaction of users	Qualitative results (any outcome)
Nissen M. 2019*	HPV vaccination series completion increased by nearly 13%	.	.	.	.
Marchand-Ciriello L. 2020*	Although there was improvement in vaccine initiation, there was no significant difference in HPV vaccine initiation rates pre- and postintervention (p=0.09)	.	.	Out of 12 participants who completed the survey, 9 identified the EMR prompt as the most effective, 7 identified the educational intervention as moderately effective, and 8 identified the monthly e-mail updates as least effective to assist the provider to improve HPV vaccine initiation rates	.
Olshefski R.S. 2018*	During year 1 of the project, 19.8% of the on-therapy oncology patients were not offered the vaccine. This percentage dropped to 2.1% in year 2 and was sustained under 4% for the duration of the multiyear project	.	.	.	.
Vinci D.M. 2021*	After the intervention, 67.42% of patients received the first dose. Patients were more likely to receive a first dose of the HPV vaccination after the intervention (p<0.001).<br>A total of 79.69% received the second dose after the intervention. Patients were less likely to receive the second dose of the HPV vaccination after the intervention (p<0.001)	.	.	.	.
Mazzoni S.E. 2016*	The rate of influenza vaccination increased from 35.4% to 46.0%. After adjustments, patients remained significantly more likely to have been vaccinated during the intervention period (p<0.001).<br>The rate of Tdap vaccination increased from 87.6% to 94.5% (p<0.001).<br>The rate of HPV vaccination increased from 7.1% to 23.7% (p<0.001).	.	.	.	.
Lin C. 2016****; Nowalk M.P. 2017*;Zimmerman R.K. 2017****	After 9 months, the intervention group increased HPV initiation 10.2 PP compared with 7.3 PP in control group (p<0.001). HPV series completion rates did not significantly differ between groups.	.	.	.	.
McGaffey A. 2019*	HPV vaccine initiation for all patients 9–26 years increased from 69.7% to 81.0% (p<0.001)<br>Overall HPV vaccine completion increased from 49.5% to 62.0% (p=0.002). Among adolescents	.	.	.	.

	(11–17 years), changes in HPV completion for females (17.4 PP, p=0.867) and males (23.4 PP, p=0.125) were not statistically significant.				
Farmar A.M. 2016***	After the intervention, HPV coverage with more than 1 dose was 89.8% (females) and 89.3% (males), compared with national rates of 57.3% and 34.6%	.	.	.	.
Deshmukh U. 2018*	After the intervention, there was a significant change in the trend of women who had both completed (p<0.01) and initiated (p=0.01) the series, with a monthly rate of rise that was 2.71 and 3.76 times higher than the pre-intervention trend (respectively)	.	.	.	.
Orefice R. 2019*	After the intervention, significant increase in women who were vaccinated for influenza (from 35.0% to 79.8%, RR 2.27 95% CI 1.92−2.64; p<0.001)	.	.	.	.
Dehlinger C. 2021*	The rate of records of pregnant women associated with a documented influenza vaccination were higher in the intervention season (58.7% vs 63.2%, p=0.01). The number of records without a vaccination code was significantly less in the intervention season (13.9% vs 22.9%; p<0.001).	.	.	.	.
Jina a. 2019*	Before the intervention, 56.9% women received Tdap during their current pregnancy; after the intervention, 64.5% (absolute difference of 7.6%, p<0.01)	.	.	.	.
O'Leary S.T. 2019****	There were not significant differences between intervention and control arms for uptake of influenza vaccine among pregnant women, with both study arms increasing their uptake. After the intervention, 29% of pregnant women in intervention group received influenza vaccine versus 41% in control group (p= 0.15).Tdap vaccines were similar to influenza vaccines.	.	.	.	.
Perkins R.B. 2020a****;Perkins R.B. 2020b°;Drainoni M.L. 2021°	Initiation coverage increased from 75% (preintervention) to 84% (intervention) to 90% (postintervention), and series completion increased from 60% (preintervention) to 63% (intervention) to 69% (postintervention) (p<0.001 for all comparisons).	The proportion of participants reporting that the "HPV vaccine is one of the most important adolescent vaccines" augmented from 71% pre to 100% post-intervention (p=0.03)	Participants felt more comfortable offering the HPV vaccine to adolescents under the age of 13 post-intervention compared with pre-intervention (71.4% pre vs. 100% post; p=0.02)	.	Participants reported that the intervention led to communication changes by increasing their knowledge, reframing the HPV vaccine as a routine vaccination, and providing tools for engaging with parents.
McLean H.Q. 2017**	HPV (first-dose) coverage:<br>Adolescents aged 11—12 years: Intervention	.	.	.	.

	group: from 40.6% to 59.3%, control group: from 31.9% to 44.5% (p=0.0002).<br>Adolescents aged 13—17 years: Intervention group: from 53.0% to 61.7%, control group: from 48.4% to 55.4% (p=0.001).<br><br>HPV vaccine series completion:<br>Adolescents aged 11—12 years: Intervention group: from 32.0% to 52.7%, control group: from 31.6% to 52.3% (p=1.0).<br>Adolescents aged 13—17 years: Intervention group: from 59.4% to 71.9%, control group: from 55.5% to 66.9%(p=0.08).				
Boey L. 2021*	After the intervention, the mean vaccination coverage significantly increased from 54% (range: 35–72%) in 2016 to 68% (range: 45–81%)	37.1% of participants were not vaccinated in 2016 and they were subsequently vaccinated in 2017.<br>Participants were less likely to expect side effects after influenza vaccination (p=0.03), to underestimate the danger of influenza (p=0.001), or to oppose vaccination in general (p=0.04).<br>On the other hand, participants were less likely to consider themselves as a risk group for influenza (p=0.01).<br>Although most participants find it important not to infect residents, they were less likely to agree with this after the campaign (p=0.02).	.	.	.
Choi N. 2017*	In 2014 vaccine coverage rates among 13- to 17-year-old female adolescents significantly increased to 78.1% for ≥1 dose and 52.6% for ≥3 doses (2013: 57.6% and 36.5%, respectively).<br>Among male adolescents and all adolescents, there were significant increases in ≥1-dose and ≥2-dose coverage estimates.	.	.	.	.
Jacobs-Wingo J.L. 2017*	In the intervention group: HPV vaccine initiation increased from 47% to 71% (mean, 24%; range,	.	.	.	.

	8%–38%) and completion increased from 20% to 42% (mean, 22%; range, 4%–35%)				
Cates J.R. 2018**	The estimated hazard ratio (HR) for initiation was 1.17 (p=0.004) during the intervention and 1.11 (p=0.005) post-intervention.<br>In the intervention group, completion was 17% higher during the intervention and 30% higher post-intervention (p=0.03)	.	.	.	.
Malone K. 2016*	Percentage of visits where the vaccine was given increased from 4% to 33% (first 6 months) and 8.7% (after one year).<br>Percentage of transplant patients receiving the PCV13 and PPSV23 from 6% to 52% (after one year)	.	.	.	.
Gingold J.A. 2016°	The mean difference between baseline and postintervention age-adjusted percent up-to-date was 3 percentage points. Seven (44%) practices gained more than 6 percentage points. Two practices met or exceeded the 90% coverage benchmark.	.	.	.	Obstacles: difficulties with electronic record systems, rigid management structures, competing priorities, and parental resistance. Facilitators: linkage with regional immunization registries, positive social interactions, and performance feedback.
Fisher-Borne M. 2018****	Among 11- to 12-year-olds, HPV vaccine series initiation increased by 14.6 percentage points (range: −18.7 to +66.4). Although HPV second dose rates increased too, the increase was not statistically significant. HPV series completion rates decreased slightly, but the change was not statistically significant. Considered separately, only the $90,000 group's HPV series initiation and meningococcal vaccination increase was significant.	.	.	.	.
Sanderson M. 2017**	Receiving HPV vaccine during the initial visit: intervention group=45.4%, control group=32.9% (adjusted RR = 1.18, CI 0.87-1.60).<br>Completion of the three-dose vaccine series (12-month follow-up): significantly lower in the intervention group vs the control group (12.4% versus 18.0%; adjusted RR=0.50, CI=0.29-0.88).	.	.	.	.
Spelman J. F. 2022***	After the intervention, 24.1% received subsequent vaccination (compared with 13.6% before intervention) (p=0.036)	.	.	.	.
Giles M. L. 2021*	Influenza vaccine coverage increased between 50 and 196% from baseline.	.	.	.	.

Suryadevara M. 2019*	After the intervention, the percent change in vaccine series completion rates ranged from 12% to 20% for 11-12-year-olds, and from 7% to 23% for 13-18-year-olds	.	.	.	.
Leila R.A. 2021*	.	.	.	.	.
Dempsey A.F. 2018****;Reno J.E. 2018*;Reno J.E. 2018*	Proportion of eligible adolescents initiating the vaccine series: 1.8% increase control vs 11.3% increase intervention; 9.5–absolute PP difference, (p<0.001).<br>Considering adjusted values, adolescents in the intervention group had higher odds of HPV vaccine series initiation (aOR 1.46; 95% CI, 1.31-1.62) and completion (aOR 1.56; 95% CI, 1.27-1.92) than the control group	.	.	Of all the various intervention components, the fact sheet was the most often used (30.5%−52.7%).<br>The disease images were the least used (0.7%−6.1%).<br>Across all of the intervention components, providers reported that the fact sheet and motivational interviewing were most useful.	.
Chin J. 2021*	The mean vaccination rate before the intervention was 9.25-13.60%. The mean vaccination rate after the intervention 91.34%	.	.	.	.
Casalino E. 2018*	Before the intervention, influenza vaccination coverage was 33.2%. At the end of the intervention, influenza vaccination coverage was 65.9% (p<0.001)	.	.	.	.
Kepka D. 2021*	HPV vaccination: missed opportunities decreased significantly from the pre-intervention to the post-intervention (21.6 vs. 8.1%, p=0.002)	.	.	.	.
Costello J. 2019*	All 38 paediatric patients found for the intervention were brought up-to-date on the needed pneumococcal vaccines	.	.	.	.
Garbutt J.M 2018°	.	.	.	.	Facilitators and barriers were identified across the domains: most distinguishing factors related to provider characteristics, their perception of the intervention, and their process to deliver the vaccine.
Kaufman J. 2020***	.	.	.	Training completion: it completed by 76% of participants.<br>Acceptability of the intervention: all participants who completed the survey were 'somewhat' or 'very' satisfied with the intervention	.
Zaidi S. 2020°	.	.	.	.	Out of 26, 25 participants reported that the app was easy to use and 23 reported a high likelihood of error

					with manual documentation. Vaccinators also reported that the GPS tracking improved the reliability of vaccination encounter reports. All vaccinators reported they mostly used the digital data to identify children overdue for vaccinations.

*uncontrolled pre-post study; **non-randomized controlled trial; ***only post intervention evaluation; **** (cluster) randomized controlled trial; °qualitative or mixed method design.

References

Abdalla A, Thomas D, McKeirnan KC, Khalifa S. Fitness to Administer Influenza Vaccine by Pharmacists in the UAE. Infect Dis Clin Pract. 2020;29:e165–8.

Abdulla E, Johnson J, Munir S, O'Dwyer R. Assessing primary health care nurses' knowledge toward immunizations: A quantitative study. J Public health Res. 2020;9:1716.

Abou Leila R, Salamah M, El-Nigoumi S. Reducing COVID-19 Vaccine Hesitancy by Implementing Organizational Intervention in a Primary Care Setting in Bahrain. Cureus. 2021;13:e19282.

Amare AT, Toni AT, Mekonnen ZA, Endehabtu BF, Tilahun BC. Effectiveness and Feasibility of Using Local Medical Universities for Capacity Building to Improve the Immunization Program in Ethiopia: Quasi-Experimental Study. J Multidiscip Healthc. 2021;14:9–19.

Arogundade L, Akinwumi T, Molemodile S, Nwaononiwu E, Ezika J, Yau I, et al. Lessons from a training needs assessment to strengthen the capacity of routine immunization service providers in Nigeria. BMC Health Serv Res. 2019;19:664.

Austin JD, Rodriguez SA, Savas LS, Megdal T, Ramondetta L, Fernandez ME. Using Intervention Mapping to Develop a Provider Intervention to Increase HPV Vaccination in a Federally Qualified Health Center. Front public Heal. 2020;8:530596.

Barton SM, Calhoun AW, Bohnert CA, Multerer SM, Statler VA, Bryant KA, et al. Standardized Vaccine-Hesitant Patients in the Assessment of the Effectiveness of Vaccine Communication Training. J Pediatr. 2022;241:203–211.e1.

Bechini A, Moscadelli A, Sartor G, Shtylla J, Guelfi MR, Bonanni P, et al. Impact assessment of an educational course on vaccinations in a population of medical students. J Prev Med Hyg. 2019;60:E171–7.

Berenson AB, Hirth JM, Chang M, Kuo Y-F, Richard P, Jones DL. A brief educational intervention can improve nursing students' knowledge of the human papillomavirus vaccine and readiness to counsel. Hum Vaccin Immunother. 2021;17:1952–60.

Berenson AB, Hirth JM, Fuchs EL, Chang M, Rupp RE. An educational intervention to improve attitudes regarding HPV vaccination and comfort with counseling among US medical students. Hum Vaccin Immunother. 2020;16:1139–44.

Bishop JM, Real FJ, McDonald SL, Klein M, DeBlasio D, Kahn JA, et al. Evaluation of HPV Vaccine: Same Way, Same Day TM : A Pilot Study. J Health Commun. 2021;26:839–45.

Blake H, Fecowycz A, Starbuck H, Jones W. COVID-19 Vaccine Education (CoVE) for Health and Care Workers to Facilitate Global Promotion of the COVID-19 Vaccines. Int J Environ Res Public Health. 2022;19:653.

Boey L, Roelants M, Vandermeulen C. Increased vaccine uptake and less perceived barriers toward vaccination in long-term care facilities that use multi-intervention manual for influenza campaigns. Hum Vaccin Immunother. 2021;17:673–80.

Bonville CA, Domachowske JB, Suryadevara M. A quality improvement education initiative to increase adolescent human papillomavirus (HPV) vaccine completion rates. Hum Vaccin Immunother. 2019;15:1570–6.

Bradley CL, Vance E. Comparison of a single day versus a multi-day immunization certificate scheduling for student pharmacists. Curr Pharm Teach Learn. 2021;13:868–74.

Bradley-Ewing A, Lee BR, Doctor JN, Meredith G, Goggin K, Myers A. A pilot intervention combining assessment and feedback with communication training and behavioral nudges to increase HPV vaccine uptake. Hum Vaccin Immunother. 2022;18.

Bratic JS, Cunningham RM, Belleza-Bascon B, Watson SK, Guffey D, Boom JA. Longitudinal Evaluation of Clinical Decision Support to Improve Influenza Vaccine Uptake in an Integrated Pediatric Health Care Delivery System, Houston, Texas. Appl Clin Inform. 2019;10:944–51.

Brewer NT, Hall ME, Malo TL, Gilkey MB, Quinn B, Lathren C. Announcements Versus Conversations to Improve HPV Vaccination Coverage: A Randomized Trial. Pediatrics. 2017;139.

Brewer NT, Mitchell CG, Alton Dailey S, Hora L, Fisher-Borne M, Tichy K, et al. HPV vaccine communication training in healthcare systems: Evaluating a train-the-trainer model. Vaccine. 2021;39:3731–6.

Brodie N, McPeak KE. Improving Human Papilloma Virus Vaccination Rates at an Urban Pediatric Primary Care Center. Pediatr Qual Saf. 2018;3:e098.

Buenger LE, Webber EC. Clinical Decision Support in the Electronic Medical Record to Increase Rates of Influenza Vaccination in a Pediatric Emergency Department. Pediatr Emerg Care. 2020;36:e641–5.

Casalino E, Ghazali A, Bouzid D, Antoniol S, Kenway P, Pereira L, et al. Emergency Department influenza vaccination campaign allows increasing influenza vaccination coverage without disrupting time interval quality indicators. Intern Emerg Med. 2018;13:673–8.

Cates JR, Crandell JL, Diehl SJ, Coyne-Beasley T. Immunization effects of a communication intervention to promote preteen HPV vaccination in primary care practices. Vaccine. 2018;36:122–7.

Cates JR, Diehl SJ, Fuemmeler BF, North SW, Chung RJ, Hill JF, et al. Toward Optimal Communication About HPV Vaccination for Preteens and Their Parents: Evaluation of an Online Training for Pediatric and Family Medicine Health Care Providers. J Public Heal Manag Pract. 2020;26:159–67.

Chamberlain AT, Limaye RJ, O'Leary ST, Frew PM, Brewer SE, Spina CI, et al. Development and acceptability of a video-based vaccine promotion tutorial for obstetric care providers. Vaccine. 2019;37:2532–6.

Chang C-Y, Hwang G-J, Gau M-L. Promoting students' learning achievement and self-efficacy: A mobile chatbot approach for nursing training. Br J Educ Technol. 2022;53:171–88.

Chase AJ, Clark MA, Rogalska A, Musselman M. Cultivating Patient-Physician Communication About Vaccination Through Vaccine Metaphors. Med Sci Educ. 2020;30:1015–7.

Chen G, Kazmi M, Chen D, Phillips J. Improving Medical Student Clinical Knowledge and Skills Through Influenza Education. Med Sci Educ. 2021;31:1645–51.

Chen H, Zhang X, Wang W, Zhang R, Du M, Shan L, et al. Effect of an educational intervention on human papillomavirus (HPV) knowledge and attitudes towards HPV vaccines among healthcare workers (HCWs) in Western China. Hum Vaccin Immunother. 2021;17:443–50.

Chidume T, Jones MC, Lambert AW, Yordy M. Preparing Students for Difficult Conversations with Patients and Families. Clin Simul Nurs. 2020;46:62–5.

Chin J, Zhou Y, Chen CL, Lomiguen CM, McClelland S, Lee-Wong M. Influenza Vaccination Quality Improvement as a Model for COVID-19 Prophylaxis. Cureus. 2021. https://doi.org/10.7759/cureus.12549.

Choi N, Curtis CR, Loharikar A, Fricchione M, Jones E, Balzer E, et al. Successful Use of Interventions in Combination to Improve Human Papillomavirus Vaccination Coverage Rates Among Adolescents—Chicago, 2013 to 2015. Acad Pediatr. 2018;18:S93–100.

Ciemins EL, Jerry M, Powelson J, Leaver-Schmidt E, Joshi V, Casanova D, et al. Impact of a Learning Collaborative Approach on Influenza and Pneumococcal Immunization Rates in US Adults: A Mixed Methods Approach. Popul Health Manag. 2020;23:29–37.

Cieslowski B, Brock L, Richesson RL, Silva S, Kim H. Optimization of Nursing-Specific Flu Alerts. CIN Comput Informatics, Nurs. 2020;38:433–40.

Coleman A, Lehman D. A Flipped Classroom and Case-Based Curriculum to Prepare Medical Students for Vaccine-Related Conversations with Parents. MedEdPORTAL. 2017. https://doi.org/10.15766/mep_2374-8265.10582.

Costello J. Immunization Recommendations for Pediatric Patients with Chronic Kidney Disease, Nephrotic Syndrome, and Renal Transplants: A Literature Review and Quality Improvement Project. Nephrol Nurs J J Am Nephrol Nurses' Assoc. 2019;46:413–45.

Cotter JC, Wilson KJ, Mallonee LF. Impact of HPV Immunization Training on Dental Hygiene Students' Attitudes and Confidence Regarding HPV Preventive Education. J Dent Educ. 2020;84:88–93.

Dawson R, Lemmon K, Trivedi NJ, Hansen S. Improving human papilloma virus vaccination rates throughout military treatment facilities. Vaccine. 2018;36:1361–7.

Dehlinger C, Nypaver C, Whiteside J. Use of an Evidence-Based Approach to Improve Influenza Vaccination Uptake in Pregnancy. J Midwifery Womens Health. 2021;66:360–5.

Dempsey AF, Pyrznawoski J, Lockhart S, Barnard J, Campagna EJ, Garrett K, et al. Effect of a Health Care Professional Communication Training Intervention on Adolescent Human Papillomavirus Vaccination. JAMA Pediatr. 2018;172:e180016.

Deshmukh U, Oliveira CR, Griggs S, Coleman E, Avni-Singer L, Pathy S, et al. Impact of a clinical interventions bundle on uptake of HPV vaccine at an OB/GYN clinic. Vaccine. 2018;36:3599–605.

Drainoni M-L, Biancarelli D, Jansen E, Bernstein J, Joseph N, Eun TJ, et al. Provider and Practice Experience Integrating the Dose-HPV Intervention into Clinical Practice. J Contin Educ Health Prof. 2021;Publish Ah.

Dybsand LL, Hall KJ, Ulven JC, Carson PJ. Improving Provider Confidence in Addressing the Vaccine-Hesitant Parent: A Pilot Project of 2 Contrasting Communication Strategies. Clin Pediatr (Phila). 2020;59:87–91.

Evans L, Matley E, Oberbillig M, Margetts E, Darrow L. HPV Knowledge and Attitudes Among Medical and Professional Students at a Nevada University: A Focus on Oropharyngeal Cancer and Mandating the Vaccine. J Cancer Educ. 2020;35:774–81.

Farmar A-LM, Love-Osborne K, Chichester K, Breslin K, Bronkan K, Hambidge SJ. Achieving High Adolescent HPV Vaccination Coverage. Pediatrics. 2016;138.

Fiks AG, Luan X, Mayne SL. Improving HPV Vaccination Rates Using Maintenance-of-Certification Requirements. Pediatrics. 2016;137.

Fiorito TM, Krilov LR, Nonaillada J. Human Papillomavirus Knowledge and Communication Skills: A Role-Play Activity for Providers. MedEdPORTAL. 2021. https://doi.org/10.15766/mep_2374-8265.11150.

Fisher-Borne M, Preiss AJ, Black M, Roberts K, Saslow D. Early Outcomes of a Multilevel Human Papillomavirus Vaccination Pilot Intervention in Federally Qualified Health Centers. Acad Pediatr. 2018;18:S79–84.

Frederick KD, Gatwood JD, Atchley DR, Rein LJ, Ali SG, Brookhart AL, et al. Exploring the early phase of implementation of a vaccine-based clinical decision support system in the community pharmacy. J Am Pharm Assoc. 2020;60:e292–300.

Gagneur A, Bergeron J, Gosselin V, Farrands A, Baron G. A complementary approach to the vaccination promotion continuum: An immunization-specific motivational-interview training for nurses. Vaccine. 2019;37:2748–56.

Garbutt JM, Dodd S, Walling E, Lee AA, Kulka K, Lobb R. Theory-based development of an implementation intervention to increase HPV vaccination in pediatric primary care practices. Implement Sci. 2018;13:45.

Gatwood J, Renfro C, Hagemann T, Chiu C-Y, Kapan S, Frederick K, et al. Facilitating pneumococcal vaccination among high-risk adults: Impact of an assertive communication training program for community pharmacists. J Am Pharm Assoc. 2021;61:572–580.e1.

Giduthuri JG, Purohit V, Maire N, Kudale A, Utzinger J, Schindler C, et al. Influenza vaccination of pregnant women: Engaging clinicians to reduce missed opportunities for vaccination. Vaccine. 2019;37:1910–7.

Giles ML, Khai K, Krishnaswamy S, Bellamy K, Angliss M, Smith C, et al. An evaluation of strategies to achieve greater than 90% coverage of maternal influenza and pertussis vaccines including an economic evaluation. BMC Pregnancy Childbirth. 2021;21:771.

Gilkey MB, Parks MJ, Margolis MA, McRee A-L, Terk J V. Implementing Evidence-Based Strategies to Improve HPV Vaccine Delivery. Pediatrics. 2019;144.

Gingold JA, Briccetti C, Zook K, Gillespie CW, Gubernick RS, Moon RY, et al. Context Matters. Clin Pediatr (Phila). 2016;55:825–37.

Glanternik JR, McDonald JC, Yee AH, Howell BA A, Saba KN, Mellor RG, et al. Evaluation of a Vaccine-Communication Tool for Physicians. J Pediatr. 2020;224:72–78.e1.

Hastings TJ, Hohmann LA, Huston SA, Ha D, Westrick SC, Garza KB. Enhancing pharmacy personnel immunization-related confidence, perceived barriers, and perceived influence: The We Immunize program. J Am Pharm Assoc. 2020;60:344–351.e2.

Heaton PC, Altstadter B, Hogea C, Poston S, Ghaswalla P. The impact of community pharmacy utilization of immunization information systems on vaccination rates: Results of a clustered randomized controlled trial. J Am Pharm Assoc. 2022;62:95–103.e2.

Irving SA, Groom HC, Stokley S, McNeil MM, Gee J, Smith N, et al. Human Papillomavirus Vaccine Coverage and Prevalence of Missed Opportunities for Vaccination in an Integrated Healthcare System. Acad Pediatr. 2018;18:S85–92.

Jacobs-Wingo JL, Jim CC, Groom A V. Human Papillomavirus Vaccine Uptake: Increase for American Indian Adolescents, 2013–2015. Am J Prev Med. 2017;53:162–8.

Jina A, Wang TL, Seyferth ER, Cohen A, Bernstein HH. Increasing antepartum Tdap vaccine administration: A quality improvement initiative. Vaccine. 2019;37:3654–9.

Jones KM, Carroll S, Hawks D, McElwain C-A, Schulkin J. Efforts to Improve Immunization Coverage during Pregnancy among Ob-Gyns. Infect Dis Obstet Gynecol. 2016;2016:1–9.

Kaufman J, Attwell K, Tuckerman J, O'Sullivan J, Omer SB, Leask J, et al. Feasibility and acceptability of the multi-component P3-MumBubVax antenatal intervention to promote maternal and childhood vaccination: A pilot study. Vaccine. 2020;38:4024–31.

Kawczak S, Mooney M, Mitchner N, Senatore V, Stoller JK. The impact of a quality improvement continuing medical education intervention on physicians' vaccination practice: a controlled study. Hum Vaccin Immunother. 2020;16:2809–15.

Kepka D, Christini K, McGough E, Wagner A, Del Fiol G, Gibson B, et al. Successful Multi-Level HPV Vaccination Intervention at a Rural Healthcare Center in the Era of COVID-19. Front Digit Heal. 2021;3.

Kim RH, Day SC, Small DS, Snider CK, Rareshide CAL, Patel MS. Variations in Influenza Vaccination by Clinic Appointment Time and an Active Choice Intervention in the Electronic Health Record to Increase Influenza Vaccination. JAMA Netw Open. 2018;1:e181770.

Koski K, Lehto JT, Hakkarainen K. Simulated Encounters With Vaccine-Hesitant Parents: Arts-Based Video Scenario and a Writing Exercise. J Med Educ Curric Dev. 2018;5:238212051879025.

Krishnaswamy S, Wallace EM, Buttery J, Giles ML. Strategies to implement maternal vaccination: A comparison between standing orders for midwife delivery, a hospital based maternal immunisation service and primary care. Vaccine. 2018;36:1796–800.

Kumar MM, Boies EG, Sawyer MH, Kennedy M, Williams C, Rhee KE. A Brief Provider Training Video Improves Comfort With Recommending the Human Papillomavirus Vaccine. Clin Pediatr (Phila). 2019;58:17–23.

Lepiller Q, Bouiller K, Slekovec C, Millot D, Mazué N, Pourchet V, et al. Perceptions of French healthcare students of vaccines and the impact of conducting an intervention in health promotion. Vaccine. 2020;38:6794–9.

Lin CJ, Nowalk MP, Pavlik VN, Brown AE, Zhang S, Raviotta JM, et al. Using the 4 pillarsTM practice transformation program to increase adult influenza vaccination and reduce missed opportunities in a randomized cluster trial. BMC Infect Dis. 2016;16:623.

Lin JL, Bacci JL, Reynolds MJ, Li Y, Firebaugh RG, Odegard PS. Comparison of two training methods in community pharmacy: Project VACCINATE. J Am Pharm Assoc. 2018;58:S94–S100.e3.

Loiacono MM, Nelson CB, Grootendorst P, Webb MD, Lee Hall L, Kwong JC, et al. Impact of a peer comparison intervention on seasonal influenza vaccine uptake in community pharmacy: A national cluster randomized study. J Am Pharm Assoc. 2021;61:539–546.e5.

Malo TL, Hall ME, Brewer NT, Lathren CR, Gilkey MB. Why is announcement training more effective than conversation training for introducing HPV vaccination? A theory-based investigation. Implement Sci. 2018;13:57.

Malone K, Clark S, Palmer JA, Lopez S, Pradhan M, Furth S, et al. A quality improvement initiative to increase pneumococcal vaccination coverage among children after kidney transplant. Pediatr Transplant. 2016;20:783–9.

Marchand-Ciriello L, Foustoukos A, Collins Fantasia H. Intervention to Increase Human Papillomavirus Vaccine Initiation Rates in Adolescent Males. J Nurse Pract. 2020;16:79–82.

Marotta C, Raia DD, Ventura G, Casuccio N, Dieli F, D’Angelo C, et al. Improvement in vaccination knowledge among health students following an integrated extra curricular intervention, an explorative study in the University of Palermo. J Prev Med Hyg. 2017;58:E93–8.

Maurici M, Arigliani M, Dugo V, Leo C, Pettinicchio V, Arigliani R, et al. Empathy in vaccination counselling: a survey on the impact of a three-day residential course. Hum Vaccin Immunother. 2019;15:631–6.

Mazzoni SE, Brewer SE, Pyrzanowski JL, Durfee MJ, Dickinson LM, Barnard JG, et al. Effect of a multi-modal intervention on immunization rates in obstetrics and gynecology clinics. Am J Obstet Gynecol. 2016;214:617.e1-617.e7.

McFadden SM, Ko LK, Shankar M, Ibrahim A, Berliner D, Lin J, et al. Development and evaluation of an online continuing education course to increase healthcare provider self-efficacy to make strong HPV vaccine recommendations to East African immigrant families. Tumour Virus Res. 2021;11:200214.

McGaffey A, Lombardo NP, Lamberton N, Klatt P, Siegel J, Middleton DB, et al. A "Sense"-ational HPV Vaccination Quality Improvement Project in a Family Medicine Residency Practice. J Natl Med Assoc. 2019;111:588–99.

McLean HQ, VanWormer JJ, Chow BDW, Birchmeier B, Vickers E, DeVries E, et al. Improving Human Papillomavirus Vaccine Use in an Integrated Health System: Impact of a Provider and Staff Intervention. J Adolesc Heal. 2017;61:252–8.

Mitchell G, Leonard L, Carter G, Santin O, Brown Wilson C. Evaluation of a "serious game" on nursing student knowledge and uptake of influenza vaccination. PLoS One. 2021;16:e0245389.

Morhardt T, McCormack K, Cardenas V, Zank J, Wolff M, Burrows H. Vaccine Curriculum to Engage Vaccine-Hesitant Families: Didactics and Communication Techniques With Simulated Patient Encounter. MedEdPORTAL. 2016. https://doi.org/10.15766/mep_2374-8265.10400.

Nissen M, Kerkvliet JL, Polkinghorn A, Pugsley L. Increasing Rates of Human Pipillomavirus Vaccination in Family Practice: A Quality Improvement Project. South Dakota medicine : the journal of the South Dakota State Medical Association. 2019;72:354–60.

Nold L, Deem MJ. A Simulation Experience for Preparing Nurses to Address Refusal of Childhood Vaccines. J Nurs Educ. 2020;59:222–6.

Nowalk MP, Moehling KK, Zhang S, Raviotta JM, Zimmerman RK, Lin CJ. Using the 4 Pillars to increase vaccination among high-risk adults: who benefits? Am J Manag Care. 2017;23:651–5.

O'Donnell M, Shurpin K, Janotha B. Improving herpes zoster vaccine rates: The impact of a targeted educational program. J Am Assoc Nurse Pract. 2018;30:435–40.

O'Leary ST, Pyrzanowski J, Brewer SE, Sevick C, Miriam Dickinson L, Dempsey AF. Effectiveness of a multimodal intervention to increase vaccination in obstetrics/gynecology settings. Vaccine. 2019;37:3409–18.

Oliver K, Beskin K, Noonan L, Shah A, Perkins R, Humiston S. A Quality Improvement Learning Collaborative for Human Papillomavirus Vaccination. Pediatr Qual Saf. 2021;6:e377.

Olshefski RS, Bibart M, Frost R, Wood E, Hampl J, Mangum R, et al. A multiyear quality improvement project to increase influenza vaccination in a pediatric oncology population undergoing active therapy. Pediatr Blood Cancer. 2018;65:e27268.

Onello E, Friedrichsen S, Krafts K, Simmons G, Diebel K. First year allopathic medical student attitudes about vaccination and vaccine hesitancy. Vaccine. 2020;38:808–14.

Orefice R, Quinlivan JA. Improving vaccination of pregnant women against seasonal influenza through use of a mandatory field in the obstetric electronic medical record. J Public Health (Bangkok). 2021;43:420–4.

Pahud B, Elizabeth Williams S, Lee BR, Lewis KO, Middleton DB, Clark S, et al. A randomized controlled trial of an online immunization curriculum. Vaccine. 2020;38:7299–307.

Pampena E, Vanucci R, Johnson LB, Bind MA, Tamayo I, Welch K, et al. Educational Interventions on Human Papillomavirus for Oral Health Providers. J Cancer Educ. 2020;35:689–95.

Patel MS, Volpp KG, Small DS, Wynne C, Zhu J, Yang L, et al. Using Active Choice Within the Electronic Health Record to Increase Influenza Vaccination Rates. J Gen Intern Med. 2017;32:790–5.

Percy JN, Crain J, Rein L, Hohmeier KC. The impact of a pharmacist-extender training program to improve pneumococcal vaccination rates within a community chain pharmacy. J Am Pharm Assoc. 2020;60:39–46.

Perkins RB, Banigbe B, Fenton AT, O'Grady AK, Jansen EM, Bernstein JL, et al. Effect of a multi-component intervention on providers' HPV vaccine communication. Hum Vaccines Immunother. 2020;16:2736–43.

Perkins RB, Legler A, Jansen E, Bernstein J, Pierre-Joseph N, Eun TJ, et al. Improving HPV vaccination rates: A stepped-wedge randomized trial. Pediatrics. 2020;146.

Persell SD, Lewin N, Yagci B, Lee JY, Oberoi SK, Orelind E, et al. Measured Performance and Vaccine Administration After Decision Support and Office Workflow Changes for Influenza Vaccination. J Healthc Qual. 2020;42:333–40.

Rand CM, Schaffer SJ, Dhepyasuwan N, Blumkin A, Albertin C, Serwint JR, et al. Provider communication, prompts, and feedback to improve HPV vaccination rates in resident clinics. Pediatrics. 2018;141.

Rand CM, Tyrrell H, Wallace-Brodeur R, Goldstein NPN, Darden PM, Humiston SG, et al. A Learning Collaborative Model to Improve Human Papillomavirus Vaccination Rates in Primary Care. Acad Pediatr. 2018;18:S46–52.

Rao S, Ziniel SI, Khan I, Dempsey A. Be inFLUential: Evaluation of a multifaceted intervention to increase influenza vaccination rates among pediatric inpatients. Vaccine. 2020;38:1370–7.

Real FJ, DeBlasio D, Beck AF, Ollberding NJ, Davis D, Cruse B, et al. A Virtual Reality Curriculum for Pediatric Residents Decreases Rates of Influenza Vaccine Refusal. Acad Pediatr. 2017;17:431–5.

Real FJ, Rosen BL, Bishop JM, McDonald S, DeBlasio D, Kreps GL, et al. Usability Evaluation of the Novel Smartphone Application, HPV Vaccine: Same Way, Same Day, Among Pediatric Residents. Acad Pediatr. 2021;21:742–9.

Reno JE, O'Leary S, Garrett K, Pyrzanowski J, Lockhart S, Campagna E, et al. Improving Provider Communication about HPV Vaccines for Vaccine-Hesitant Parents Through the Use of Motivational Interviewing. J Health Commun. 2018;23:313–20.

Reno JE, O'Leary ST, Pyrzanowski J, Lockhart S, Thomas J, Dempsey AF. Evaluation of the Implementation of a Multicomponent Intervention to Improve Health Care Provider Communication About Human Papillomavirus Vaccination. Acad Pediatr. 2018;18:882–8.

Rosen BL, Real FJ, Bishop JM, McDonald SL, Klein M, Kahn JA, et al. School Health Service Provider Perceptions on Facilitated Interactive Role-Play Around HPV Vaccine Recommendation. J Cancer Educ. 2022;37:1286–95.

Salous MH, Bind MA, Granger L, Johnson LB, Welch K, Villa A. An educational intervention on HPV knowledge and comfortability discussing vaccination among oral health care professionals of the American Indian and Alaskan Native population. Hum Vaccines Immunother. 2020;16:3131–7.

Sanderson M, Canedo JR, Khabele D, Fadden MK, Harris C, Beard K, et al. Pragmatic trial of an intervention to increase human papillomavirus vaccination in safety-net clinics. BMC Public Health. 2017;17.

Sandokji I, Anderson LS, Warejko JK, Emerson BL, Greenberg JH. An initiative to improve pneumococcal immunization counseling in children with nephrotic syndrome. Pediatr Nephrol. 2022;37:1333–8.

Schnaith AM, Evans EM, Vogt C, Tinsay AM, Schmidt TE, Tessier KM, et al. An innovative medical school curriculum to address human papillomavirus vaccine hesitancy. Vaccine. 2018;36:3830–5.

Serino L, Maurici M, D'alò GL, Amadori F, Terracciano E, Zaratti L, et al. Healthcare workers training courses on vaccinations: A flexible format easily adaptable to different healthcare settings. Vaccines. 2020;8:1–12.

Shukla A, Nyambose J, Vanucci R, Johnson LB, Welch K, Lind E, et al. Evaluating the Effectiveness of Human Papillomavirus Educational Intervention among Oral Health Professionals. J Cancer Educ. 2019;34:890–6.

Skoy ET, Kelsch M, Hall K, Choi BJ, Carson P. Increasing adult immunization rates in a rural state through targeted pharmacist education. J Am Pharm Assoc. 2020;60:e301–6.

Spelman JF, Kravetz JD, Bastian L, Ruser C. Addressing COVID-19 Vaccine Acceptance Within a Large Healthcare System: a Population Health Model. J Gen Intern Med. 2022;37:954–7.

Spina CI, Brewer SE, Ellingson MK, Chamberlain AT, Limaye RJ, Orenstein WA, et al. Adapting Center for Disease Control and Prevention's immunization quality improvement program to improve maternal vaccination uptake in obstetrics. Vaccine. 2020;38:7963–9.

Srirangan K, Lavenue A. Helping Québec Pharmacists Seize the Vaccination Service Opportunity: The Pharmacy Best Practice Workshops. Pharmacy. 2021;9:51.

Steiner CR, Dechant J, Brungo L, Cassidy B. An Evidence-based Protocol to Improve HPV Vaccine Initiation Rates at a County Immunization Clinic. J Community Health Nurs. 2021;38:73–84.

Stetson RC, Fang JL, Colby CE, Jacobson RM. Improving infant vaccination status in a Level IV neonatal intensive care unit. Pediatrics. 2019;144.

Suryadevara M, Bonville CA, Cibula DA, Domachowske JB. Cancer Prevention Education for Providers, Staff, Parents, and Teens Improves Adolescent Human Papillomavirus Immunization Rates. J Pediatr. 2019;205:145–152.e2.

Szilagyi PG, Humiston SG, Stephens-Shields AJ, Localio R, Breck A, Kelly MK, et al. Effect of Training Pediatric Clinicians in Human Papillomavirus Communication Strategies on Human Papillomavirus Vaccination Rates: A Cluster Randomized Clinical Trial. JAMA Pediatr. 2021;175:901–10.

Tchoualeu DD, Harvey B, Nyaku M, Opare J, Traicoff D, Bonsu G, et al. Evaluation of the impact of immunization second year of life training interventions on health care workers in ghana. Glob Heal Sci Pract. 2021;9:498–507.

Torabizadeh C, Nick N, Vizeshfar F, Jamalimoghadam N, Bagheri S. Effectiveness of an Educational Intervention to Increase Human Papillomavirus Knowledge and Attitude in Staff and Nursing Students. J Community Health Nurs. 2020;37:214–21.

Traicoff D, Tchoualeu DD, Opare J, Wardle M, Quaye P, Sandhu HS, et al. Applying adult learning best practices to design immunization training for health careworkers in ghana. Glob Heal Sci Pract. 2021;9:487–97.

Vinci DM, Ryan J, Howard M, Snider D, Strahan B, Smith G, et al. Increasing Human Papillomavirus Vaccination in a Federally Qualified Health Center Organization Using a Systems-Based Intervention Integrating EHR and Statewide Immunization Information System. J Community Health. 2022;47:53–62.

Visalli G, Facciolà A, Mazzitelli F, Laganà P, Di Pietro A. Health education intervention to improve vaccination knowledge and attitudes in a cohort of obstetrics students. J Prev Med Hyg. 2021;62:E110–6.

Vyas D, Galal SM, Rogan EL, Boyce EG. Training students to address vaccine hesitancy and/or refusal. Am J Pharm Educ. 2018;82:944–53.

Wallace-Brodeur R, Li R, Davis W, Humiston S, Albertin C, Szilagyi PG, et al. A quality improvement collaborative to increase human papillomavirus vaccination rates in local health department clinics. Prev Med (Baltim). 2020;139.

Werk LN, Diaz MC, Cadilla A, Franciosi JP, Hossain MJ. Promoting Adherence to Influenza Vaccination Recommendations in Pediatric Practice. J Prim Care Community Heal. 2019;10.

Wermers R, Ostroski T, Hagler D. Health care provider use of motivational interviewing to address vaccine hesitancy in college students. J Am Assoc Nurse Pract. 2021;33:86–93.

Whitaker JA, Poland CM, Beckman TJ, Bundrick JB, Chaudhry R, Grill DE, et al. Immunization education for internal medicine residents: A cluster-randomized controlled trial. Vaccine. 2018;36:1823–9.

Wiley R, Shelal Z, Bernard C, Urbauer D, Toy E, Ramondetta L. Team-Based Learning Module for Undergraduate Medical Education: a Module Focused on the Human Papilloma Virus to Increase Willingness to Vaccinate. J Cancer Educ. 2019;34:357–62.

Wilkinson TA, Dixon BE, Xiao S, Tu W, Lindsay B, Sheley M, et al. Physician clinical decision support system prompts and administration of subsequent doses of HPV vaccine: A randomized clinical trial. Vaccine. 2019;37:4414–8.

Williams SE, Adams LE, Sommer EC. Improving Vaccination for Young Children (IVY): A Stepped-Wedge Cluster Randomized Trial. Acad Pediatr. 2021;21:1151–60.

Zaidi S, Shaikh SA, Sayani S, Kazi AM, Khoja A, Hussain SS, et al. Operability, acceptability, and usefulness of a mobile app to track routine immunization performance in rural Pakistan: Interview study among vaccinators and key informants. JMIR mHealth uHealth. 2020;8.

Zimet G, Dixon BE, Xiao S, Tu W, Kulkarni A, Dugan T, et al. Simple and Elaborated Clinician Reminder Prompts for Human Papillomavirus Vaccination: A Randomized Clinical Trial. Acad Pediatr. 2018;18:S66–71.

Zimmerman RK, Moehling KK, Lin CJ, Zhang S, Raviotta JM, Reis EC, et al. Improving adolescent HPV vaccination in a randomized controlled cluster trial using the 4 PillarsTM practice Transformation Program. Vaccine. 2017;35:109–17.
